# Quantifying CO_2_ Insertion Equilibria for Low-Pressure Propene Oxide and Carbon Dioxide Ring Opening Copolymerization Catalysts

**DOI:** 10.1021/jacs.3c13959

**Published:** 2024-04-08

**Authors:** Katharina H. S. Eisenhardt, Francesca Fiorentini, Wouter Lindeboom, Charlotte K. Williams

**Affiliations:** Department Chemistry, University of Oxford, Chemistry Research Laboratory, 12 Mansfield Road, Oxford OX1 3TA, U.K.

## Abstract

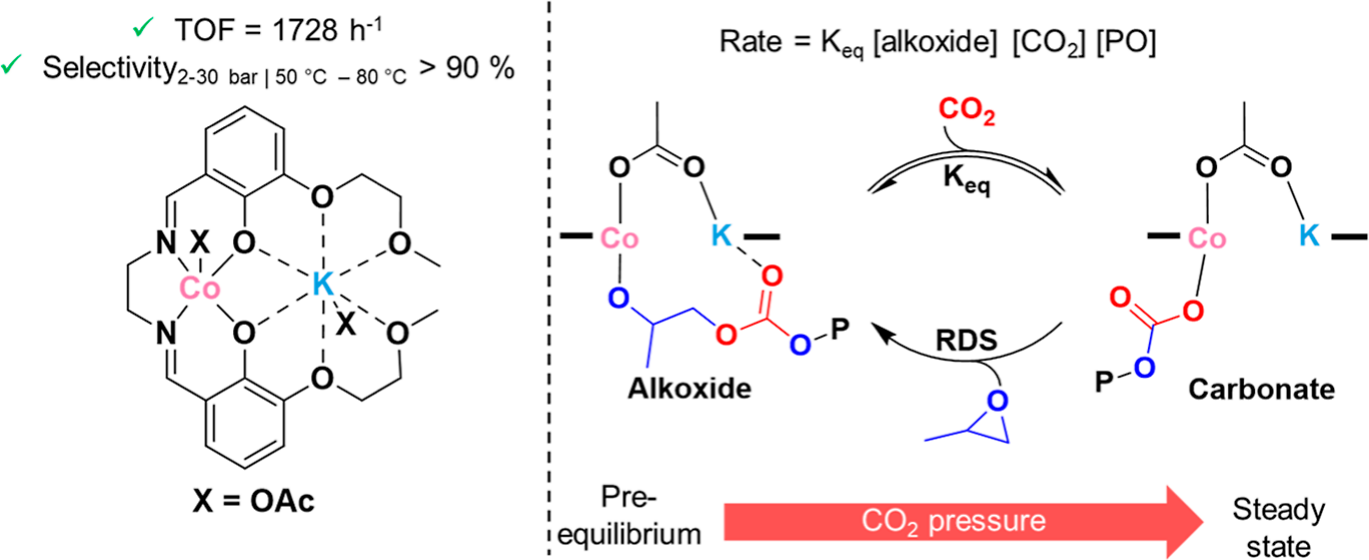

While outstanding catalysts are known for the ring-opening copolymerization (ROCOP) of CO_2_ and propene oxide (PO), few are reported at low CO_2_ pressure. Here, a new series of Co(III)M(I) heterodinuclear catalysts are compared. The Co(III)K(I) complex shows the best activity (TOF = 1728 h^–1^) and selectivity (>90% polymer, >99% CO_2_) and is highly effective at low pressures (<10 bar). CO_2_ insertion is a prerate determining chemical equilibrium step. At low pressures, the concentration of the active catalyst depends on CO_2_ pressure; above 12 bar, its concentration is saturated, and rates are independent of pressure, allowing the equilibrium constant to be quantified for the first time (*K*_eq_ = 1.27 M^–1^). A unified rate law, applicable under all operating conditions, is presented. As proof of potential, published data for leading literature catalysts are reinterpreted and the CO_2_ equilibrium constants estimated, showing that this unified rate law applies to other systems.

## Introduction

**Figure 1:**
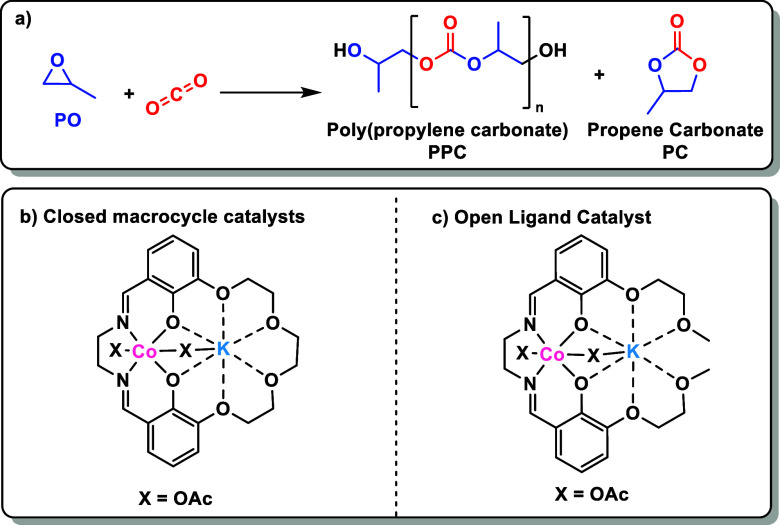
Ring opening copolymerization (ROCOP) of PO and carbon dioxide. (a) Polymerization reaction yielding PPC and, in some cases, byproduct propene carbonate (PC). (b) Previously reported heterodinuclear catalysts featuring a “closed” macrocyclic ligand^31^ and the novel Co(III)M(I) “open” ligand catalysts presented here.

Carbon dioxide utilization is an essential technology to valorize industrial wastes, diversify the range of renewable C_1_-feedstocks to replace virgin petrochemicals, and reduce chemical manufacturing pollution.^1−3^ Carbon dioxide and epoxide ring opening copolymerization (ROCOP) is a CCU frontrunner technology since it also produces valuable polycarbonates, i.e., it is both environmentally and economically attractive.^4−6^ The most advanced process, in terms of large scale manufacturing and product properties, is propene oxide (PO)/CO_2_ ROCOP (Figure 1). When applied with alcohols, it furnishes hydroxyl end-capped low-molar mass polypropene carbonate (PPC), which is useful as a surfactant in the production of polyurethane foams, as well as coatings, adhesives, sealants, and elastomers.^7−9^ PO/CO_2_ ROCOP is truly catalytic and applies large-scale commercial reagents since PO is manufactured on ∼12 Mt/annum scale and is already used to make polyether polyols;^10^ and PPC contains up to 43 wt % CO_2_, reducing the embedded emissions compared with existing polyols. The process’s viability depends both on high performance catalysts and on identifying low-energy operating conditions. Both features help to control selectivity since PO and carbon dioxide coupling yield either polymer [PPC or poly(ether carbonates)] or cyclic carbonate (PC), with the latter generally being the thermodynamic reaction product.^11−13^ There have been some tremendous advances in catalysis, particularly in homogeneous catalysis, with the lead catalysts reaching impressive TOFs (>1000 h^–1^) and polymer selectivity (>99%).^5,14−30^

Examining the optimum process conditions for the best catalysts reveals a “low pressure (*P*) and higher temperature (*T*) process conditions gap”. Almost all catalysts require high CO_2_ pressures of at least 20 bar for decent activity and selectivity, with only three catalysts being quantified below 20 bar. At 14 bar, a cobalt(III)salen(Cl)/PPNCl catalyst reported by Coates and co-workers showed a TOF of 620 h^–1^ and 99% selectivity for polymers.^22^ At 10 bar, a multinuclear CaCo_3_ catalyst, reported by Nozaki and Mashima, showed an activity of 115 h^–1^ and 99% PPC selectivity.^21^ At 5 bar CO_2_ pressure, we reported a heterodinuclear Co(III)K(I) catalyst with an activity of 258 h^–1^ and 87% PPC selectivity (Figure 1b).^31^ A trinuclear cobalt catalyst was claimed to be active at 1 bar CO_2_, but without any quantification of rates or selectivity.^32^

Additionally, even when applied under high carbon dioxide pressures, most catalysts experience a major decline in polymer selectivity with increasing temperatures.^5,12,29,31,33,34^ For example, both the chromium(III)salen(X)/PPNX catalysts and the di-Zn(II) catalysts formed mostly propene carbonate (PC) byproducts when used above 80 °C.^5,12,29,33^

The heterodinuclear Co(III)K(I) catalyst showed a decrease in PPC selectivity from >99 to 63% as temperature increased from 50 to 70 °C (at constant 20 bar CO_2_ pressure).^31^ A cobalt(III)(porphyrin-R_4_)^**4+**^·4X^**–**^ catalyst, with four ammonium substituents showed a PPC selectivity drop from 96% at 50 °C to 70% at 60 °C (40 bar CO_2_ pressure).^34^ Such a selectivity decline is detrimental since the removal of the byproduct PC is energy-intensive due to its high boiling point (242 °C).^7^ The detrimental impacts upon PPC selectivity of conducting polymerizations at low carbon dioxide pressures, particularly when combined with operable process temperatures (50–80 °C), are especially problematic when considering larger-scale operations. Polymerizations are best conducted at higher temperatures (50–80 °C) to reduce the polymer viscosity (which is lowest at high temperatures) and to maximize rates (activity) and conversions (productivity). Current polyether polyol production, by PO ring opening polymerization (ROP), occurs in plants operating at <10 bar pressure for safety reasons.^35,36^ One attractive possibility would be PO/CO_2_ ROCOP catalysts that operate effectively at <10 bar pressure; such processes may be suitable for use/retrofit in current manufacturing plants and should improve safety and energy efficiency, i.e., by reducing carbon dioxide compression emissions.^35,36^ To achieve this goal, new catalysts must bridge the “low *P*, high *T* process conditions gap”.

In the literature, most catalysts are optimized for high activity, with prior structure–activity relationships focused on the influences of the metals,^31,37,38^ ligands^14,24,25,39,40^ or initiators.^22^ Also, catalysts are generally described under individually selected (optimized) operating conditions, and there is no standard set of conditions for comparisons. There are fewer investigations of how catalyst performances vary with conditions, e.g., temperatures and pressures. There is also very little examination of catalyst structure-selectivity relationships, with most authors attributing selectivity declines to “thermodynamic” factors.^5^ However, last year, we hypothesized that the catalyst structure, as well as conditions, may be important in controlling process selectivity.^11^ A detailed experimental and computational (DFT) investigation using the closed Co(III)K(I) catalyst identified carbon dioxide insertion as the “selectivity limiting” step.^11^

## Results

### Co(III)M(I) Catalysts

**Figure 2:**
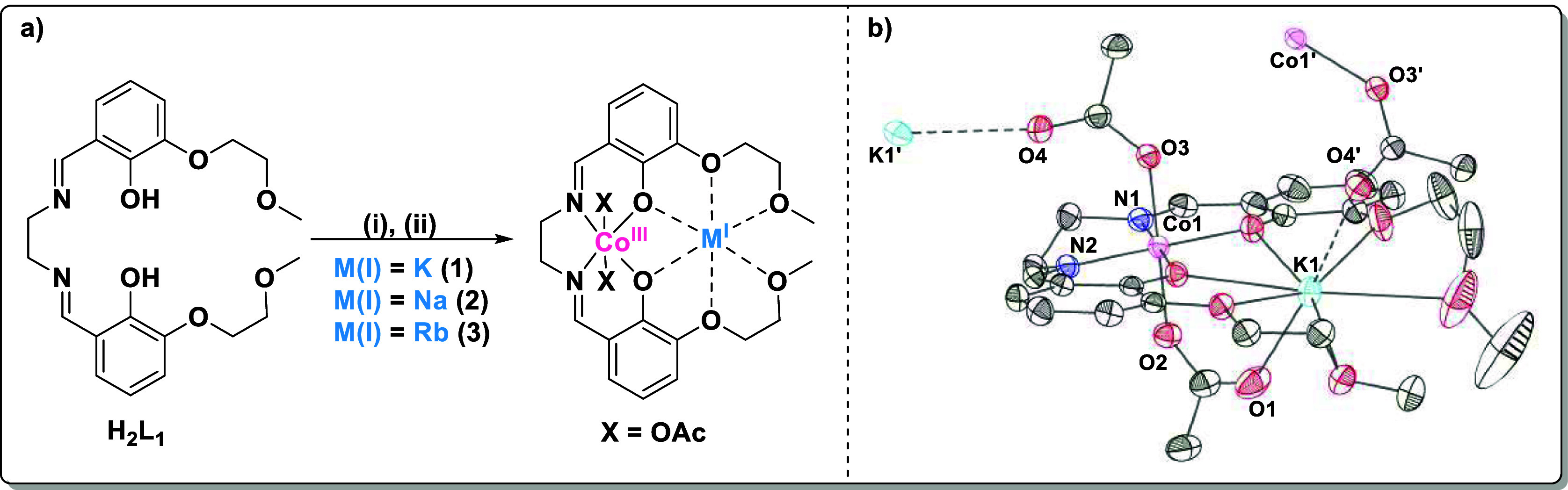
Synthesis of catalysts **1–3** from the “open” ancillary ligand H_2_L_1_. Reagents and conditions: (i) Co(II)(OAc)_2_, KOAc (1)/NaOAc (2)/RbOAc (3), MeCN, 16 h, under N_2_. (ii) 2 equiv of AcOH, air. (b) ORTEP representation of the molecular structure of catalyst 1, with hydrogen atoms and residual solvents omitted for clarity (see Figure S28a, Tables S15–S17 for complete structure since Co(III)K(I) is polymeric in the solid state).

In CO_2_/epoxide ROCOP catalysis, our goal was to replace the functionality of the expensive cocatalyst salt (PPNCl) with an earth-abundant s-block metal. As early as 2008, Lu and co-workers attempted exactly such an approach but discovered that direct cocatalyst replacement by group 1 salts resulted in mostly cyclic carbonate formation rather than the polymer.^41^ In 2020, we reported the first heterodinuclear Co(III)M(I) catalysts operating without any cocatalyst.^31^ In these catalysts, the s-block metal was coordinated by the ancillary ligand, close to (3–4 Å) the active Co(III) site.^31^ The macrocyclic ligand features with both a Schiff base coordination environment (pocket), for Co(III) coordination, and crown ether binding pocket, for s-block metal coordination.^11,31,40,42^ The ligand was used to make a series of Co(III)M(I) complexes, where M = Na(I), K(I), and Rb(I), which were successful PO/CO_2_ ROCOP catalysts (Figure 1b).^31^ Here, a new “open” ligand was targeted, which is close structurally to the macrocycle used previously but differs only by the removal of a single C–C bond connecting the “ether” units. The ligand design and metals selected were deliberately “close” to the prior work to allow for systematic evaluation of factors influencing rates and selectivity. The new ligand also features a Schiff base coordination environment for Co(III) and two separate ethers for s-block metal coordination (Figure 1c). The ligand was synthesized in high yield from commercial precursors by modifications to literature procedures (see Supporting Information for details).^31,43^ Catalysts **1**–**3** were synthesized by reacting them with Co(II)(OAc)_2_ and the appropriate MOAc precursor, where M = K(I) (**1**), Na(I) (**2**), and Rb(I) (**3**) (Figures 2 and S1–S28). The desired heterodinuclear Co(III)M(I) complexes were isolated after oxidation in air. They were all fully characterized, including by single crystal X-ray diffraction (Figures 2 and S28a, Tables S15–S17), infrared (IR) spectroscopy (Figure S11), NMR spectroscopy (Figures S1–S10 and S12–S28), and cyclic voltammetry (Figure S27), all of which confirm the dinuclear complex formation.

Single crystals suitable for X-ray diffraction experiments were obtained for all three complexes by the slow diffusion of diethyl ether into a saturated catalyst solution in chloroform. Structural elucidation confirmed the heterodinuclear complexes have phenolate oxygen atoms that bridge both metals, the Co(III) was coordinated by the Schiff base donors and the M(I) by the “open” ether donors (Figures 2 and S28a). In the solid state, the K(I) and Rb(I) catalysts, **1** and **3**, are polymers with two different acetate coordination modes, one bridging between the two metals in the same ligand, and the other bridging between different metals in adjacent ligands. Catalysts **1** and **3** have M(I) coordinated by all six oxygen atoms. In contrast, catalyst **2** (Co(III)Na(I)) is a monomer in the solid state and Na(I) is 7-coordinate rather than the 8-coordinate structures observed for K(I) and Rb(I) in catalysts **1** and **3**, respectively. The Na(I) atom, in catalyst **2**, is equatorially coordinated by five of the ligand oxygen atoms and the sixth, O6, coordinates in an axial position that is approximately perpendicular to the plane formed by Na(I) and Co(III) (Figures 2 and S28a). Within the series of complexes, the metal acetate bond length (M(I)–O(1)) increases with the M(I) ionic radius, from 2.3079(17) Å for **2**, to 2.725(2) Å for **1** and 2.844(2) Å for **3**.

### PO/CO_2_ ROCOP Catalysis

Catalysts **1**–**3** were tested for the PO/CO_2_ ROCOP at loadings of catalyst/diol/epoxide: 1:20:4000 under 20 bar of CO_2_ and 50 °C (Table 1). All catalysts yield the desirable polyols, as evidenced by *M*_n_ values which are <10 kg/mol and narrow, monomodal dispersity values (*D̵* < 1.10). The polymerizations were monitored in a Parr reactor fitted with a DiComp sentinel probe, attached to an ATR-IR spectrometer, allowing for in situ monitoring of conversion vs time data by changes to the intensity of signals assigned to PPC (1750 cm^–1^) and PC (1810 cm^–1^). The polymer conversion was independently calibrated using aliquots of the crude reaction mixture, which were analyzed by ^1^H NMR spectroscopy, with mesitylene as an internal standard.

**Table 1:** Data for the PO/CO_2_ ROCOP Using Co(III)M(I) Catalysts **1–3** and Compared with Analogous “Closed” Catalysts^31^

entry	catalyst^a^	*t*/h	productivity TON^c^	selectivity CO_2_ /%^d^	selectivity PPC /%^e^	activity TOF/h^–1^^f^	rate coefficient, *k*_obs_ 10^–5^/s^–1^^g^	PPC molar mass *M*_n_ [*D̵*]/g mol^–1^^h^
1	Co(III)Na(I) “open”	9	695	99	98	107	0.78	3500 [1.04]
2	Co(III)K(I) “open”	4	1413	>99	>99	398	3.98	8000 [1.03]
3	Co(III)Rb(I) “open”	5	867	>99	98	211	1.17	3800 [1.05]
4^31^	Co(III)Na(I) “closed”^b^	5	600	>99	79	120	0.75	2300 [1.08]
5^31^	Co(III)K(I) “closed”^b^	4	1369	>99	98	340	4.00	5900 [1.10]
6^31^	Co(III)Rb(I) “closed”^b^	23	1240	>99	91	54	0.63	6500 [1.07]

a Reaction conditions: catalyst (0.025 mol %, 3.6 mM), PO (6 mL, 14.3 M), and *trans*-1,2-cyclohexanediol (0.5 mol %, 71 mM), 20 bar CO_2_, 50 °C.

b Data from ref (31).

c Turnover number (TON) was determined by dividing the moles of epoxide consumed (PO: determined by comparison of the sum of integrals by ^1^H NMR spectroscopy of PPC (4.92 ppm, 1H), PC (4.77 ppm, 1H), and PPO (3.46–3.64 ppm, 3H) against mesitylene (0.25 mol %, 36 mM) as an internal standard.

d CO_2_ uptake was calculated by dividing the sum of integrals for polycarbonate and cyclic carbonate against the sum of integrals for polycarbonate, cyclic carbonate, and polyether.

e Polymer selectivity was determined by dividing the sum of integrals for polycarbonate and polyether against the sum of integrals for polycarbonate, cyclic carbonate, and polyether.

f Turnover frequency (TOF) was calculated by dividing the TON against time.

g *k*_obs_ determined as the gradient of the plot of ln[PO]_*t*_/[PO]_0_ vs time.

h Determined by GPC in THF using narrow dispersity polystyrene standards.

At 20 bar CO_2_, all the Co(III)M(I) catalysts should operate by the same rate law, i.e., rate = *k*_p_[Catalyst][PO] (*see later*), as was also previously established for the “closed” Co(III)K(I).^31^ Thus, for each polymerization, the conversion versus time data was converted into a semilogarithmic plot, ln([PO])/ln([PO]_0_) versus time (s). The linear fits (gradients) to these plots are the pseudo first order rate coefficients (*k*_obs_). To obtain the rate coefficients, initial rate data was applied (5–20% PO conversion) since prior research has demonstrated equivalent rate coefficients are obtained using integrated values (10–80%).^44^ The *k*_obs_ values allow for reliable comparisons between different catalysts and, in particular, between these “open” and “closed” Co(III)M(I) catalysts.

All the “open” Co(III)M(I) catalysts showed good activity and selectivity at 20 bar and 50 °C. The Co(III)K(I) catalyst (**1**) has the highest turnover frequency (TOF) of 398 h^–1^ and *k*_obs_ = 3.98 × 10^–5^ s^–1^ (Table 1, entry 2). Its performance, at this temperature, is very close to the “closed” Co(III)K(I) catalyst (TOF = 333 h^–1^, *k*_obs_ = 4.00 × 10^–5^ s^–1^, Figure 1b, Table 1, entry 5).^40^ Catalyst **1** was also highly selective, forming only PPC without any detectable cyclic carbonate byproduct (PC) (Table 1, entry 2). Comparing the influence of the s-block metal reveals that Co(III)Rb(I) (**3**) has a slightly lower, but good activity with a TOF = 211 h^–1^ (*k*_obs_ = 1.17 × 10^–5^ s^–1^) and shows 98% selectivity for PPC. In contrast, Co(III)Na(I) (**2**) is slower, with a TOF = 107 h^–1^ (*k*_obs_ = 0.78 × 10^–5^ s^–1^). The reduced performance for the Na(I) catalyst (**2** vs **1** or **3**) appears to correlate with the smaller radius of Na(I) and may be a consequence of coordinative saturation. Such a hypothesis is supported by the different ligand coordination in the solid state structures (Figures 2 and S28a). The Co(III)K(I) and Co(III)Rb(I) catalysts show similar performances and solid state structures. Also there is a difference in coordination number dependent upon ionic radius from seven coordinate Na(I) to eight coordinate K(I) being significantly larger (1.12 to 1.51 Å) than that from eight coordinate K(I) to eight coordinate Rb(I) (1.51 to 1.61 Å).^45^

Catalysts **1**–**3** show significantly greater PPC selectivity than the equivalent “closed” Co(III)M(I) catalysts (Table 1).^31^ It is possible that their increased ligand flexibility helps to reduce the transition state energies. In both series, the Co(III)K(I) combination of metals is the most active. Yet the relative activities of the Na(I) and Rb(I) catalysts are diametrically opposed in the two series. In the “open” series, the Co(III)Rb(I) catalyst, **3**, is more active than the Co(III)Na(I) catalyst **2**, whereas the opposite order applies to the “closed” catalyst series (Table 1).

These differences must arise from changes to the s-block metal coordination chemistry resulting from the different ligand structures. In the solid state, the “closed” ligand Co(III)Rb(I) catalyst shows the Rb(I) is coordinated out of plane with the crown ether pocket, as illustrated by a distance of 0.523 Å between the Rb(I) and crown ether plane (Figure S28b).^31^ In contrast, the solid state structure of the “open” Co(III)Rb(I) catalyst the Rb(I) is coplanar with the ether oxygens, as indicated by a significantly shorter distance (0.199 Å) between the ether oxygen plane and the Rb(I) center, perhaps indicating an improved ligand–metal match (Figure S28c). In the “closed” Co(III)Na(I) complex, all six ligand oxygen atoms are coordinated to the sodium in an equatorial plane, and the remaining axial coordination site is available for polymer binding. In contrast, in open complex (**2**), the axial coordination site is occupied by one of the ligand oxygen atoms, with the remainder being coordinated equatorially—i.e., polymer coordination is sterically inhibited.

### Process Conditions and the “Open” Co(III)K(I) Catalyst

Given the high activity and selectivity shown by catalyst **1**, further investigation into its performance under different conditions was conducted over the temperature range 50–80 °C (Tables 2 and S1) and pressure range 2–30 bar (Tables 2, S1, and S8). It showed excellent selectivity and high activity even at very low loadings, including at catalyst/epoxide ratios of 1:10,000 (0.001 mol %, Table 2, entry 10, Figure S29). As the reaction temperature was increased, its activity increased and reached a TOF of 1728 h^–1^ at 80 °C (*k*_obs_ = 2144 × 10^–7^ s^–1^), and, importantly, even at this temperature, its selectivity for PPC remained very high (>92%) (Table 2, entry 1–5, Table S1). These results confirm that significant rate enhancements can be achieved by conducting polymerizations at higher temperatures. Comparably, under these higher temperature conditions, the “closed” catalyst was slower, with a TOF of 833 h^–1^, and its selectivity was reduced to 63% (Table 2, entry 12).^31^ Catalyst **1** also performed exceptionally well at lower CO_2_ pressures (Table 2, entry 6–8, Tables S1 and S8). For example, even at just 5 bar of CO_2_ pressure, its activity was 273 h^–1^ and PPC selectivity was 96% (Table 2, entry 7). In comparison, the “closed” catalyst did not turnover below 5 bar.^31^ Thus, catalyst **1** performs better than the “closed” catalyst under both high-temperature and low-pressure conditions.

**Table 2:** Selected Data for the PO/CO_2_ PROCOP Using Catalyst **1** under Different Temperatures and Conditions, with “Closed” Co(III)K(I) Comparison (Entries 11–13)^a^

entry	*T*/°C	*P*/bar	*t*/h	select. CO_2_/%^b^	select. PPC/%^c^	activity TOF/h^–1^^d^	rate coefficient *k*_obs_ 10^–7^/s^e^	PPC molar mass *M*_n_[*D̵*]/g mol^–1^^f^
1	50	20	4	>99	>99 ± 0.4	328 ± 8	344 ± 0.7	8000 [1.03]
2	60	20	2	>99	99 ± 0.4	833 ± 25	833 ± 76	6900 [1.06]
3	65	20	1.5	>99	94 ± 1	821 ± 13	991 ± 80	6600 [1.11]
4	70	20	2	>99	97 ± 0.4	1045 ± 36	1388 ± 141	11,000 [1.03]
5	80	20	1	>99	92 ± 1	1728 ± 127	2144 ± 63	7100 [1.04]
6	50	2	4	>99	87 ± 0.7	183 ± 11	103 ± 12	2600 [1.09]
7	50	5	4	>99	96 ± 0.4	273 ± 49	213 ± 33	2700 [1.06]
8	50	10	3	>99	97 ± 0.7	353 ± 13	364 ± 46	5900 [1.07]
9	50	15	4	>99	99 ± 0.5	382 ± 31	322 ± 5	7200 [1.8]
10^g^	50	40	72	>99	98	73		29,700 [1.03]
11^31^ closed Co(III)K(I)	50	20	4	>99	98	340	400	5900 [1.10]
12^31^ closed Co(III)K(I)	70	20	1.4	>99	63	833	652	4100 [1.08]
13^31^ closed Co(III)K(I)	50	5	3.2	>99	87	258	190	

a Reaction conditions: catalyst (0.025 mol %, 3.6 mM), PO (6 mL, 14.3 M), and *trans*-1,2-cyclohexanediol (0.5 mol %, 71 mM) under static CO_2_ pressure.

b CO_2_ uptake was calculated by dividing the sum of integrals for polycarbonate and cyclic carbonate against the sum of integrals for polycarbonate, cyclic carbonate, and polyether.

c Polymer selectivity was determined by dividing the sum of integrals for polycarbonate and polyether against the sum of integrals for polycarbonate, cyclic carbonate, and polyether.

d Turnover frequency (TOF) was calculated by dividing the turnover number (TON) against time, where TON was determined by dividing the moles of epoxide consumed (PO: determined by comparison of the sum of integrals by ^1^H NMR spectroscopy of PPC (4.92 ppm, 1H), PC (4.77 ppm, 1H), and PPO (3.46–3.64 ppm, 3H) against mesitylene (0.25 mol %, 36 mM) as an internal standard.

e *k*_obs_ determined as the gradient of the plot of ln[PO]_*t*_/[PO]_0_ vs time.

f Determined by GPC in THF using narrow dispersity polystyrene standards. Representative values are shown.

g Catalyst (0.001 mol %, 0.71 mM), PO (20 mL, 7.15 M), 20 mL toluene, and *trans*-1,2-cyclohexanediol (0.002 mol %, 0.014 mM) under 40 bar static CO_2_ pressure. All values are reported as an average of *n* = 2 runs, with an error of ± Δ*x* = σ/√*n*.

### Kinetic Analysis of Co(III)K(I) (**1**)

Next, the polymerization rates for **1** under different CO_2_ pressures were investigated. All kinetic experiments were performed using experimental apparatus (valves and mass flow controllers), which delivered constant carbon dioxide pressure (i.e., with reactors refilling automatically upon carbon dioxide consumption). The formation of both PPC and PC was monitored in situ using the previously described ATR-IR spectrometer in the pressure vessel, allowing for the determination of the pseudo-first-order rate coefficient, *k*_obs_, from the linear fit to plots of ln([PO])/ln([PO]_0_) versus time (s) (e.g., Figure S35 and S36). All reactions were repeated, with errors generally being ±10%.

**Figure 3:**
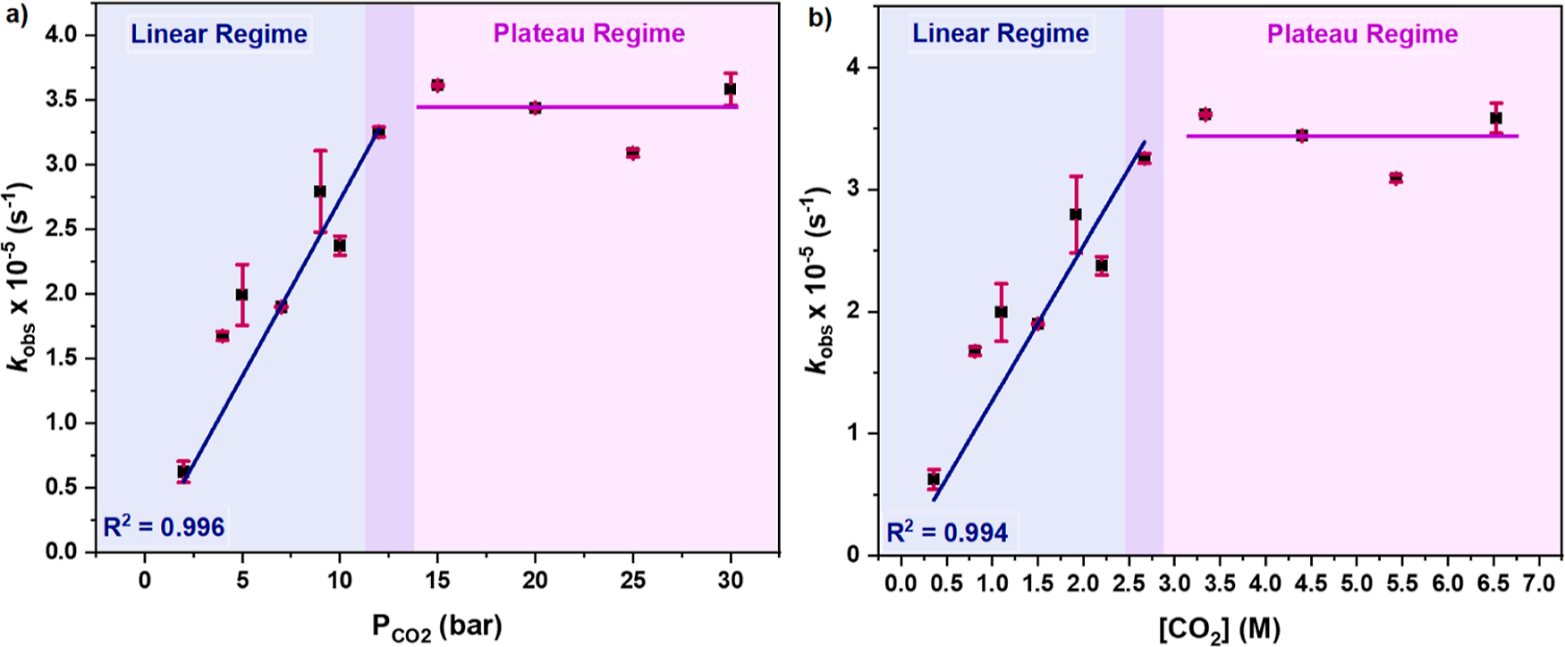
Data to determine the relationship between the polymerization rate and the CO_2_ pressure (a) or between the rate and the CO_2_ concentration (b). The pseudo rate coefficients, *k*_obs_ (s^–1^), were determined as the gradients of plots of ln([PO]/[PO]_0_) vs time (s). In each case, at lower pressures/concentrations, the rate increased linearly with pressure (linear regime). At higher pressures (>12 bar), the rate was independent of CO_2_ pressure/concentration (plateau regime) (Table S8). Polymerization conditions: 0.025 mol % catalyst, neat PO (6 mL) at 50 °C. The data are presented as averages of two runs, with errors determined from Δ*x* = σ/ (Table S8).

**Figure 4:**
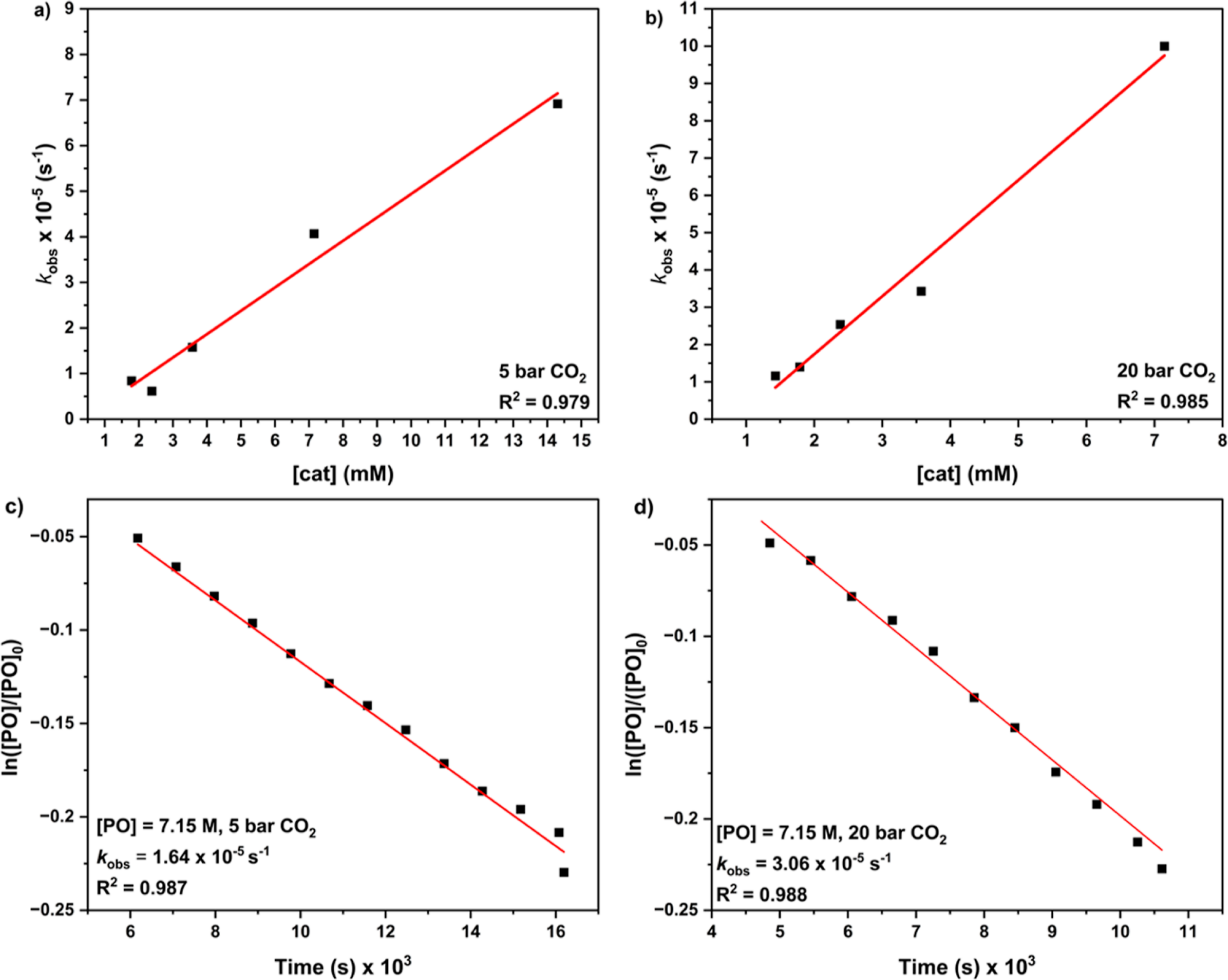
Plots were used to determine the rate dependence on catalyst and PO concentration. Rate dependences in catalyst concentration were determined from plots of *k*_obs_ vs [**1**] for polymerizations conducted at (a) 5 bar, where [**1**] = 1.79–14.3 mM, and (b) 20 bar, where [**1**] = 1.79–7.15 mM. The data presented is given in Tables S9 and S10. Rate dependence in PO concentration was determined from a linear, semilogarithmic plot of ln([PO]_*t*_/[PO]_0_) vs time for [PO] = 7.15 M. For polymerizations conducted at (b) 5 and (c) 20 bar. The data are given in Tables S11 and S12.

For polymerizations with CO_2_ pressures ranging from 2 to 12 bar, the rate coefficients (*k*_obs_) increased with pressure (Figure 3a, linear regime, Table S8). However, at pressures from 12 to 30 bar, the rate coefficients (*k*_obs_) were independent of pressure (Figure 3a, plateau regime, Table S8, entries 17–22). Such pressure dependent rates are rather unusual, and the phenomenon requires more investigation. A prior report from Cramail, Tassaing, and co-workers determined the relationship between carbon dioxide pressure and its solubility in PO; this data was used to quantify the polymerization rates versus [CO_2_] (Tables S3–S7, Figure 3b). It is important to note that even at the lowest pressure investigated (2 bar), its high solubility in PO leads to at least a 10-fold excess of CO_2_ (0.37 M) compared to the concentrations of catalyst used (3.75 mM). Any physical equilibria between dissolved and gas-phase CO_2_ should not affect the kinetic analysis.^46^ In the “low pressure” regime, plots of *k*_obs_ vs P and of *k*_obs_ vs [CO_2_] both showed linear fits to the data (Figure 3a,b). Further, plots of ln(*k*_obs_) vs ln([CO_2_]) and ln(*k*_obs_) vs ln(*P*) showed gradients of 0.9 and 1, respectively (Figure S33). These findings all indicate a first-order rate dependency in the CO_2_ concentration (or pressure). In the “higher pressure” regime, the rates plateau with increasing carbon dioxide pressure. Thus, at pressures >12 bar, plots of *k*_obs_ vs *P* and *k*_obs_ vs [CO_2_] were best fit with horizonal lines (Figure 3a,b). Further, plots of ln(*k*_obs_) vs ln([CO_2_]) and ln(*k*_obs_) vs ln(*P*) were also consistent with zero-order dependence on CO_2_ pressure (Figures 3 and S33). Next, the rate dependence on catalyst and epoxide concentrations was determined in each of the two pressure “regimes”. These measurements were conducted at both 5 and 20 bar using integrated rate treatments. On increasing the catalyst concentration, the rate increased linearly in both the 5 and 20 bar CO_2_ pressure regimes (Figure 4a,b, Tables S9 and S10, Figure S34). These data indicate a first-order rate dependence in catalyst concentration, regardless of the pressure applied. To determine the order in epoxide, at both 5 and 20 bar CO_2_ pressure, neat PO was diluted to concentrations between [PO] = 3.56–10.70 M using diethyl carbonate (DEC). DEC was selected as it shows similar catalyst and CO_2_ solubility to PO.^31^ Examining the polymerizations using integrated rate treatments showed linear fits to semilogarithmic plots of ln([PO]) vs time for all the epoxide concentrations (Figure 4c,d, Tables S11 and S12, Figures S35 and S36). These results are consistent with rates that are first order in PO concentration in both pressure regimes. Further, variable time normalized analyses (VTNA) are consistent with the proposed first order using data from both 5 and 20 bar of CO_2_ pressure experiments (Figures S37 and S38).

Overall, at lower pressures (*P* ≤ 12 bar), the rate law appears to be third order, while at higher pressures (*P* ≥ 12 bar), it is apparently second order
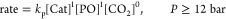

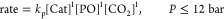


**Figure 5:**
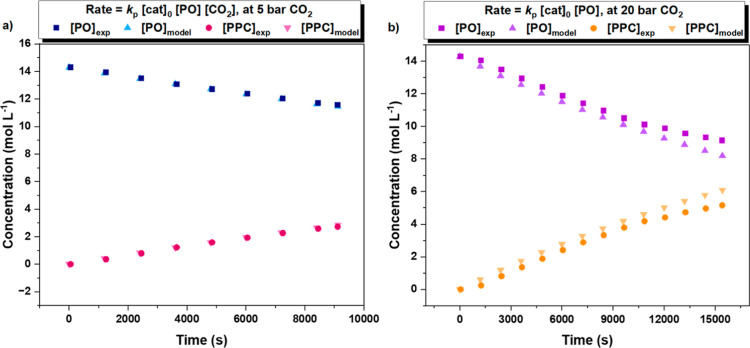
Experimental concentration vs time data (squares) and fits to the data using the rate laws (triangles) using COPASI kinetic analyses. (a) Experimental (squares) and modeled (triangles) data for experiments conducted at 5 bar CO_2_ pressure, where rate = *k*_p_[cat]_0_[PO]_0_[CO_2_], *k*_p_ = 7.57 × 10^–3^ M^–3^ s^–1^, [cat]_0_ = 3.57 mM, [PO]_0_ = 14.29 M, and [CO_2_] = 0.86 M (Table S14, entry 1). (b) Experimental (squares) and modeled data (triangles) at 20 bar CO_2_ pressure, where rate = *k*_p_[cat]_0_[PO]_0_, *k*_p_ = 9.61 × 10^–3^ M^–2^ s^–1^, [cat]_0_ = 3.57 mM, and [PO]_0_ = 14.29 M (Table S14, entry 2, Supporting Information for further details on use of COPASI).^47^

Using the kinetic modeling software COPASI, the polymerization data at both 5 and 20 bar CO_2_ pressure was modeled by the two different rate laws (Figure 5).^47^ The experimental and modeled monomer and polymer concentration vs time data showed very close agreement (Figure 5). In contrast, removing the dependence on the CO_2_ concentration from the rate law at 5 bar or introducing a CO_2_ dependence to the 20 bar rate law resulted in inferior models in both cases (Figure S39). These observations support the rate dependence on the CO_2_ concentration/pressure and reinforce it as a real kinetic effect.

### Determination of [Carbonate] and *K*_eq_

**Figure 6:**
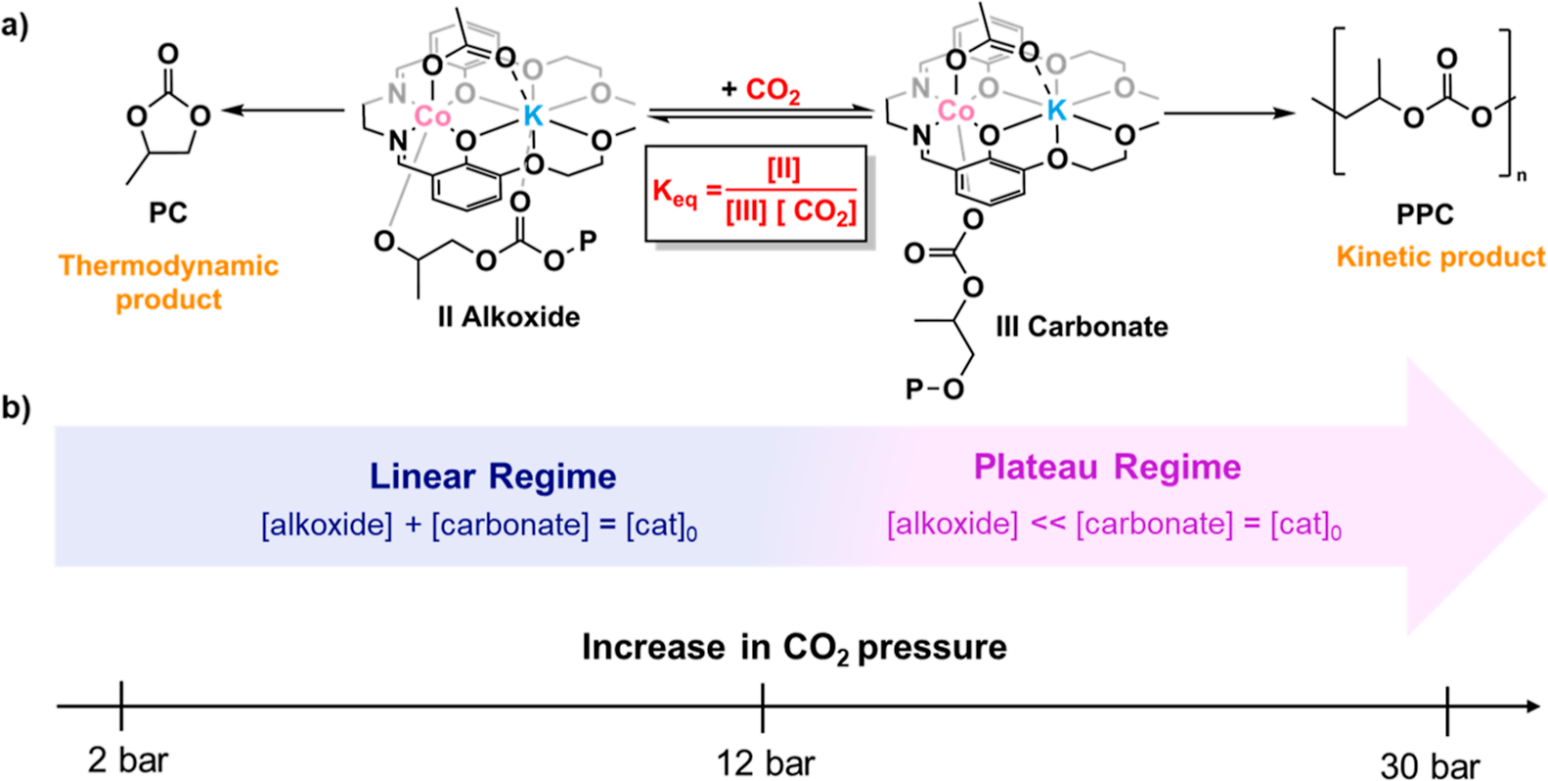
Schematic representation of (a) CO_2_ insertion equilibrium between the alkoxide and carbonate intermediate, including the formation of PPC from the carbonate intermediate. (b) Change in intermediate concentration with varying CO_2_ pressures. Calculations of the intermediate concentrations are detailed in Table S13 and the Supporting Information spreadsheet.

The previously proposed mechanism for epoxide/CO_2_ ROCOP involves the reactions of two key intermediates with the starting materials: alkoxide and carbonate species. The alkoxide intermediate reacts with CO_2_ to form the carbonate intermediate (Figure 6a).^11,31^ This step is generally proposed as the “fast” step in catalysis, and for catalyst **1** at pressures above 12 bar, it seems to be independent of the rate.

**Figure 7:**
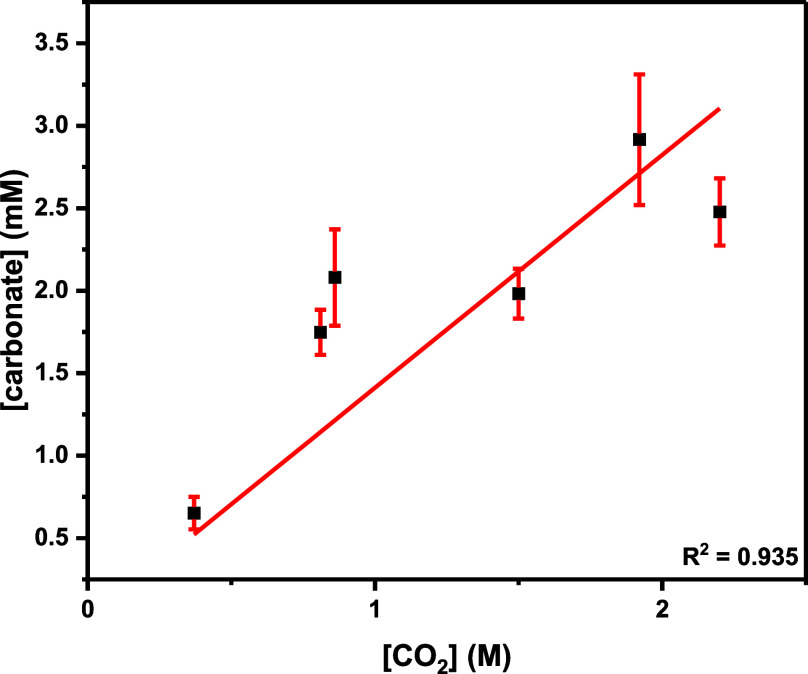
Plot of [carbonate] (mM) vs [CO_2_] (M), where [carbonate] was calculated using eq 3 (Table S13 and Supporting Information spreadsheet contains the raw data).

The carbonate intermediate reacts with the epoxide via a nucleophilic attack to form the alkoxide. At pressures above 12 bar, the process is proposed as rate limiting (Figure 6a). However, in the low pressure regime (2–12 bar), the situation is more complex, and to more accurately understand the catalyst behavior under all conditions, it warrants attention. The overall catalyst concentration must, under all pressures, be equal to the sum of the concentrations of the alkoxide and carbonate intermediates (Figure 6, eqs 1 and 2). Considering how the concentration of these species varies depending on the conditions, this suggests a prerate limiting equilibrium. Based on the different regimes observed in the activity vs [CO_2_] plot (linear and plateau regimes, Figure 3), it is proposed that at low pressures, the ratio of carbonate/alkoxide increases as the pressure increases (eq 1). At pressures >12 bar, the majority of the catalyst is present as the carbonate intermediate; hence, it becomes the effective catalytic species (eq 2, Figure 6)
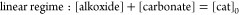
1
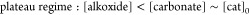
2Thus, at *P* > 12 bar, the rate observed at this pressure and above is the maximum observable for any particular set of conditions (e.g., [cat]_0_ = 3.57 mM, 50 °C). Given this assumption, it is feasible to relate any rate observed at any given pressure, *P*, to the carbonate intermediate concentration using the following expression
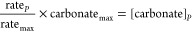
3As such, the concentration of the carbonate intermediate increases linearly with [CO_2_] (M) over the range of 2–12 bar of CO_2_ pressure (Figures 6 and 7, Table S13, Supporting Information spreadsheet), but at pressures above 12 bar, its concentration is constant, resulting in a plateau in catalytic activity (eq 2).

The change in the ratio of the intermediates when varying CO_2_ pressure in the low-pressure regime is strongly indicative of a CO_2_-insertion equilibrium (Figure 6a). Such a hypothesis is also consistent with a DFT investigation of the “closed” Co(III)K(I) catalyst, where CO_2_ insertion was proposed to be an equilibrium.^11^ In the prior work, it was not possible to estimate the equilibrium constant because the barrier to byproduct formation was lower than polymerization, preventing clean estimates of catalyst speciation.^31^ However, the new “open” Co(III)K(I) catalyst maintains very high PPC selectivity across the whole pressure range (selectivity >90%, Tables 2 and S8). Therefore, the carbon dioxide insertion equilibrium constant may be estimated at any given pressure, *P*, in the low pressure regime, by using the corresponding carbon dioxide concentration data and the estimated carbonate intermediate concentration (eqs 2 and 3) using the expression
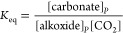
4As such, considering [carbonate]_*P*_ (eq 3), [CO_2_], and [alkoxide]_*P*_ (eq 1) at *P* = 2–10 bar (eq 4) allows for the equilibrium constant to be calculated as 1.27 ± 0.23 M^–1^ (see Supporting Information for details).

### Unified Polymerization Rate Law

With quantification and insight into the carbon dioxide insertion chemistry in hand, the two experimentally determined rate laws can be combined to give a single, unified rate law that is applicable under all conditions (pressures). To do this, the catalyst concentration considered in the experimental rate laws needs to be substituted by the carbonate intermediate concentration (or by the product of the equilibrium constant and the alkoxide intermediate concentration). The unified rate law accounts for the fact that, depending on the position of the equilibrium, only a fraction of the initially added catalyst may be active in the rate-limiting step of polymerization (i.e., PO ring opening)
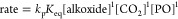
where pressure = 2–30 bar CO_2_.

**Figure 8:**
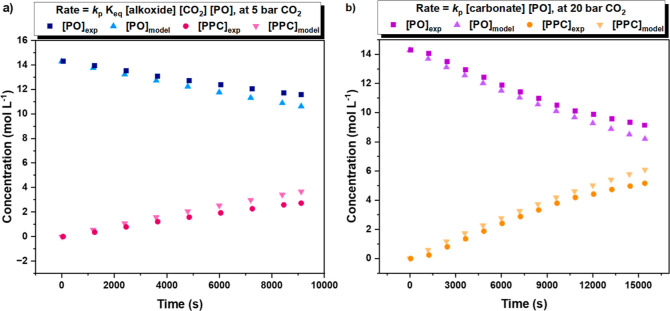
Plot showing concentration vs time data with experimental measurements (squares) and fits (triangles) using the unified rate law. (a) Experiment conducted at 5 bar CO_2_ pressure and using rate = *k*_p_*K*_eq_[alkoxide][CO_2_][PO], where *k*_p_ = 9.61 × 10^–3^ M^–2^ s^–1^, *K*_eq_ = 2.46 M^–1^, [CO_2_] = 0.86 M, and [PO] = 14.29 M. (b) Experiment conducted at 20 bar CO_2_ pressure and using rate = *k*_p_[carbonate][PO], where [carbonate] ∼ [cat]_0_ = 3.75 mM, *k*_p_ = 9.61 × 10^–3^ M^–2^ s^–1^, and [PO]_0_ = 14.29 M (Table S14 and Supporting Information for details).^47^

The unified rate law is certainly fully consistent with the experimentally observed orders in the reagents. Accordingly, at low pressures, the [alkoxide] is significant, resulting in an apparent first-order dependence on [CO_2_]. This means that *K*_eq_[alkoxide][CO_2_] < [cat]_0_, hence, rate = *k*_p_*K*_eq_[alkoxide][CO_2_][PO]. The catalyst speciation into both alkoxide and carbonate intermediates at low CO_2_ pressures is consistent with a classical pre-equilibrium assumption. The consumption of the carbonate intermediate in the rate-determining step (RDS) is too slow to affect the position of the pre-equilibrium, leading to a significant buildup of alkoxide concentration (Figure S47a).^48^ Once again, COPASI was used to model the experimental data by using the unified rate law with the associated intermediate concentrations and equilibrium constant. For polymerizations at 5 bar CO_2_ pressure, there was an excellent fit to the experimental data using the unified rate law (Figure 8a).^47^ At higher CO_2_ pressures (20 bar), the carbon dioxide insertion equilibrium sits toward the carbonate intermediate. Hence, its concentration should dominate, leading to an apparent zero order in [CO_2_]. According to *K*_eq_[alkoxide][CO_2_] = [carbonate] ∼ [cat]_0_, rate = *k*_p_[cat]_0_[PO]. This is consistent with a classical steady-state assumption: the alkoxide intermediate formed in the RDS (i.e., PO ring opening) is immediately consumed and  (Figure S47b).^48^ The COPASI modeling of the experimental data using the unified rate law and associated intermediate concentration and equilibrium constant also shows excellent fits (Figure 8b).

### Measurement of Catalyst Transition State Barriers

**Figure 9:**
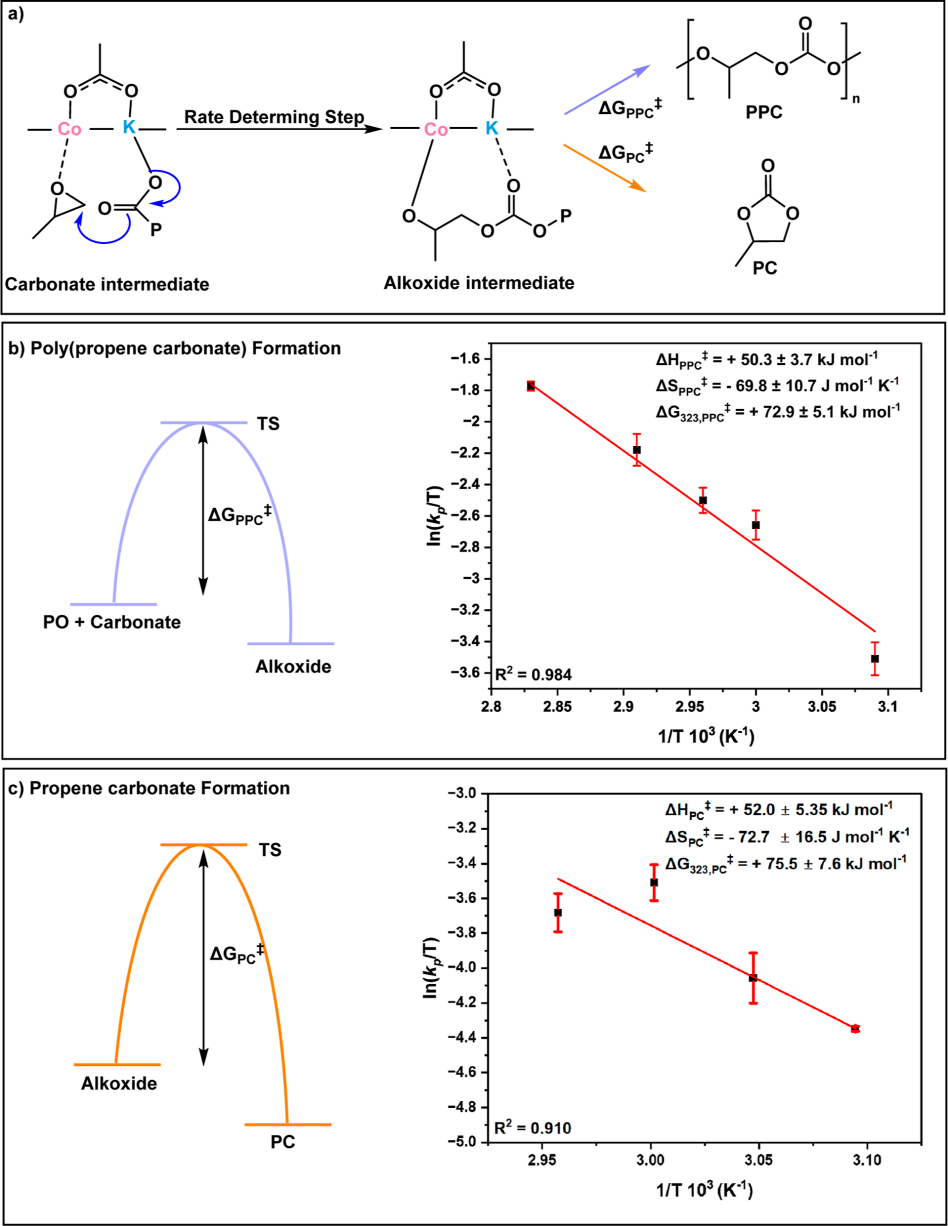
(a) Eyring analysis for the forward polymerization of PO/CO_2_ ROCOP and backbiting of PPC using catalyst **1**. (a) Schematic representation of the RDS, forming an alkoxide intermediate. (b) Schematic representation of the barrier to the forward reaction and plot of ln(*k*_p_/*T*) vs 1/*T* for the forward polymerization for 1 over the temperature range 50–80 °C (0.025 mol % catalyst, neat PO (6 mL), 1,2-cyclohexene diol (71 mM), and 20 bar CO_2_ pressure). (b) Schematic representation of the barrier to the backbiting plot of ln(*k*_p_/*T*) vs 1/*T* for backbiting for 1 over the temperature range of 45–65 °C (0.1 mol % catalyst and neat PO (6 mL)). All experimental values are reported as an average of *n* = 2 runs, with an error of ±Δ*x*. Straight lines were fitted using a weighted least-squares (WLS) method, and errors from the WLS were propagated for calculated values (Tables 2, S1, and S2).

As illustrated by the unified rate law, in the high-pressure (steady-state) regime, the CO_2_ insertion equilibrium should not influence the rate of reaction. Thus, these conditions are optimal to quantify the transition state barrier for PPC polymer formation (Δ*G*_PPC_^⧧^) (Figure 9). The previous DFT investigations using the “closed” Co(III)K(I) catalyst suggested that in the RDS, the K(I)-carbonate nucleophile, formed in the CO_2_ insertion equilibrium, attacks a Co(III)-epoxide intermediate (Figure 9). This reaction (re)forms an alkoxide intermediate, which can either take part in the CO_2_ insertion (an equilibrium process) or may “backbite” upon the chain to extrude propene carbonate (PC) and a shorter alkoxide intermediate.^11,31,38^ Thus, the carbon dioxide insertion equilibrium represents the “selectivity determining step” (Figures 6 and 9).

The transition state barrier for the formation of PPC using catalyst **1** was determined by assessing the relationship between polymerization rate and temperature in experiments conducted at 20 bar CO_2_. Using the Eyring equation, Δ*H*^⧧^ and Δ*S*^⧧^ were determined from the plot of *k*_p_/*T* vs 1/*T* (K^–1^). In line with its high activity, catalyst **1** shows a rate determining transition state barrier that is ∼20 kJ mol^–1^ lower than the analogous “closed” catalyst, i.e., for **1**, Δ*G*_PPC_^⧧^ = +72.9 ± 5.1 kJ mol^–1^ vs “closed” Co(III)K(I) catalyst, Δ*G*_PPC_^⧧^ = +92.6 kJ mol^–1^ (Figure 9b).^31,38^

In the selectivity-limiting step, the byproduct, PC, may also form, depending on the relative barriers and conditions. Hypothetically, backbiting can occur from either the carbonate or the alkoxide intermediate (see Supporting Information). An observed linear increase of the intermediate ratio of [alkoxide]/[carbonate] with an increasing PC/PPC product ratio strongly indicates that the rate of backbiting depends on the alkoxide concentration and backbiting only occurs from the alkoxide intermediate (Figure S42). This is in line with previous DFT calculations using the “closed” Co(III)K(I), which highlighted that the barrier to backbiting from the carbonate is significantly higher than that to backbiting from the alkoxide (Δ*G*_calc_^†^_(carbonate)_ = +31.9 kcal mol^–1^ vs Δ*G*_calc_^†^_(alkoxide)_ = 22.4 kcal mol^–1^).^49^ The transition state barrier for cyclic carbonate formation is conventionally determined by the rate of formation of PC byproducts during polymerization (i.e., rate vs temperature relationship). One challenge for such high-selectivity catalysts is that negligible quantities of PC form under optimal process conditions, and hence measuring concentration vs time data would be less reliable. To overcome this limitation, the PC formation barrier was approximated by the catalyzed reaction of pure PPC to form cyclic carbonate. The backbiting reaction of PPC proceeds through the reaction of the hydroxyl chain ends of the PPC, which react with the catalyst, thereby generating an alkoxide intermediate which is structurally similar to that observed in forward polymerization (Figure S41).

Thus, the rates of PC formation were measured from the reaction between PPC (hydroxyl end-capped) and the catalyst under variable temperatures (45–65 °C), with the transition state barrier then determined by Eyring analysis.^11^ The PC, cyclic carbonate, transition state energy is Δ*G*_PC_^⧧^ = +75.5 ± 7.6 kJ mol^–1^, which is lower than that observed for the “closed” catalyst (Δ*G*_PC_^⧧^ = +81.4 kJ mol^–1^).^2,3^ This could indicate that all transition states, including those relevant for PC formation from the alkoxide intermediate, have lower energy for the “open” vs “closed” catalyst (Figure 9b,c). Such an interpretation is completely consistent with the greater activity observed for **1** compared to the “closed” catalyst, particularly at higher temperatures (Tables 1 and 2).”

### Proposed Polymerization Mechanism

**Figure 10:**
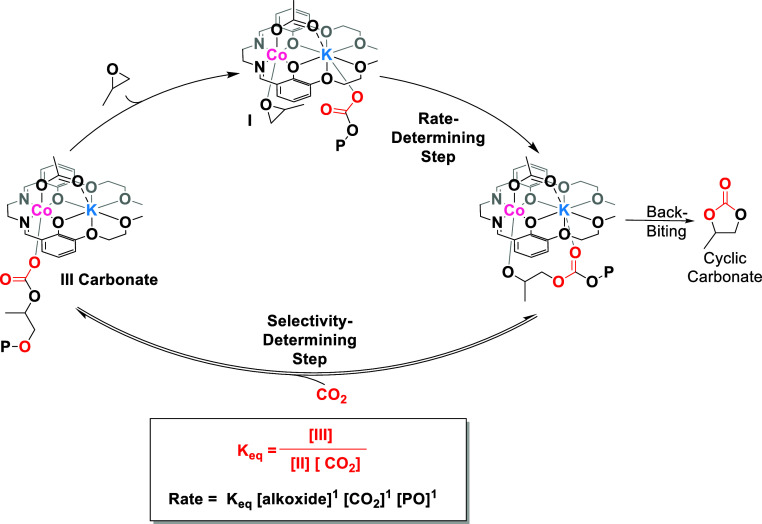
Illustration of the proposed reaction mechanism for PO/CO_2_ ROCOP using catalyst **1**, including the mechanism for PC formation, proceeding from the alkoxide intermediate. The selectivity determining the CO_2_ insertion step becomes a rate-limiting pre-equilibrium at CO_2_ pressures below 12 bar and can be described as a steady-state at high pressures.

The combined kinetic and thermodynamic data and unified rate law underpin a proposed polymerization mechanism featuring a CO_2_ insertion pre-equilibrium, which is particularly relevant at low pressures and reaches a steady-state above 12 bar (Figure 10). In the mechanism, the rate-determining step, under all conditions, remains PO ring-opening by the s-block metal carbonate intermediate. Also, under all conditions, the selectivity limiting step occurs during carbon dioxide insertion. At lower temperatures and carbon dioxide pressures, the selectivity for PPC formation remains >90%. As the pressure increases, the rate of polymerization increases since, under these conditions, a higher concentration of carbonate intermediate is present. For this catalyst, the equilibrium is driven fully to carbonate intermediate at pressures >12 bar, and the polymerization rate reaches its steady state with very high selectivity. On the other hand, at a fixed carbon dioxide pressure, increasing the polymerization temperature increases the rates, but at lower pressures, it may compromise the selectivity (since barriers to byproducts are accessible). Thus, attention should be paid at high temperatures and very low carbon dioxide pressures, as the alkoxide intermediate concentration is highest, and this may reduce both the rates and the PPC selectivity (Table 2).

## Discussion

Using the proposed mechanism, the “open” catalyst can be compared against the related “closed” Co(III)K(I) catalyst, taking into consideration the pre-equilibrium and rate law.

Catalyst **1** shows significantly higher selectivity for PPC formation at both low CO_2_ pressures and high temperatures compared with the “closed” catalyst. The data can be rationalized by the carbon dioxide insertion equilibrium, associated with **1**, being positioned further toward the carbonate intermediate than for the related closed catalyst. Such a difference in carbon dioxide insertion equilibrium is interesting since it demonstrates that even a very small change to the ancillary ligand framework can significantly influence behavior across many different conditions (becoming more apparent at low pressures and high temperatures). Catalyst **1** is the better catalyst since it has a significantly lower polymer formation barrier (∼20 kJ/mol) compared to the ’closed’ catalyst (Figure 9). Considering other low-pressure PO/CO_2_ ROCOP catalysts, catalyst **1** shows good rates, but its key performance benefit lies in its very high selectivity (i.e., favorable carbon dioxide insertion equilibrium). Its selectivity over a range of temperatures allows for the first quantification of the carbon dioxide insertion equilibrium constant and substantiates a new mechanistic hypothesis: the carbon dioxide insertion pre-equilibrium controls both rates and selectivity. Considering the polymerization energy consumption and, therefore, the GHG emissions, it favors low pressures and moderate temperatures. In addition, catalysts requiring additives, especially toxic PPNCl salts, may be best avoided. The [(salen[NBu^3+]^_4_)Co(OAc)](NO_2_)_4_ catalyst shows outstanding activity (TOF = 103,000 h^–1^) and very high selectivity (>99%, 25 bar, 80 °C).^23^ It is ∼60× more active than catalyst **1**, but does require higher pressures (25 bar). The cobalt(salen)(X)/PPNX catalyst system operates at lower temperatures and pressures, 25 °C and 14 bar; it shows excellent activity (TOF = 620 h^–1^) but requires the PPNCl cocatalyst when used without the additive, its activity drops to 80 h^–1^.^22^ Comparably, catalyst **1** exhibits a good, but lower, activity of 273 ± 49 h^–1^, with a high selectivity of 96 ± 0.4% at 5 bar and 50 °C. One benefit of catalyst **1**, compared to other literature catalysts, is that its high selectivity is retained over a range of temperatures and pressures (5–30 bar, Figure S40). No comparable performance data was reported for either of these literature benchmark PO/CO_2_ ROCOP catalysts (Figure S40).^22,23^ The carbon dioxide insertion equilibrium measured in this work is likely to be relevant to other literature catalysts. Nearly all catalysts show lower catalytic selectivity at higher temperatures and low pressures.^5,12,28,29,33,34^ The data from this work provide a better understanding since both the catalyst and process conditions influence the selectivity-limiting equilibria. The carbon dioxide insertion equilibrium hypothesis is supported by two prior reports of catalysts showing pressure dependent changes to rate laws.^28,50^ Rieger and co-workers reported [(BDI)Zn{N(SiMe_3_)_2_}] catalysts for cyclohexene oxide (CHO)/CO_2_ ROCOP, with the lead species showing “a shift in kinetic rate law with pressure”.

The authors reported a reaction order in CO_2_ pressure of one at pressures 5–10 bar, which changed to a zero order at pressures >10 bar (Figures S44 and S46). The orders of epoxide and catalyst concentrations were each measured as one over the whole pressure range. As such, the overall rate changed from third to second order with increasing CO_2_ pressures.^50^ With the insights gained in the current work, these prior data can be re-examined to quantify the equilibrium constant and fully rationalized without requiring a change in the rate-determining step. As such, an alternative explanation is that at pressures below 10 bar, the carbon dioxide insertion equilibrium influences the rates by reducing the overall concentration of the catalyst, i.e., the carbonate intermediate concentration. Whereas, at pressures above 10 bar, the equilibrium is saturated and the [carbonate] = [catalyst] (Figure S46). To test this notion, the unified rate law methods and the published carbon dioxide solubility data in CHO were used to estimate the carbon dioxide insertion equilibrium constant: *K*_eq_ ∼ 3.7 M^–1^ (see Supporting Information spreadsheet for details, Figure S46). The same team reported another outstanding dizinc catalyst, coordinated by a macrocycle ligand, for CHO/CO_2_ ROCOP (Figure S44).^28^ The kinetic analyses revealed two pressure-dependent rate laws. At carbon dioxide pressures below 25 bar, rates were first order in CO_2_ pressure but zero order in epoxide concentration.^28^ At pressures above 25 bar, rates were zero order in carbon dioxide pressure and first order in CHO concentration. Rates were first order with respect to the catalyst concentration under all pressures. The authors rationalized the data by a change in the rate-limiting step in catalysis from carbon dioxide insertion at low pressures to epoxide ring opening at high pressures. The mechanism was also investigated using DFT with similar activation energy barriers calculated for the putative rate-limiting transition states, i.e., CHO ring opening and CO_2_ insertion.^28^ Our carbon dioxide insertion equilibrium hypothesis also fully rationalizes the observations. At pressures below 25 bar, polymerizations are controlled/limited by the CO_2_ insertion equilibrium, while at higher pressures, the carbonate intermediate reaches the steady state. Applying the methods described earlier to the published data, together with the previously reported CHO solubility data, the equilibrium constant for the dizinc catalyst carbon dioxide insertion is *K*_eq_ ∼ 0.78 M^–1^ (Figure S45).

**Figure 11:**
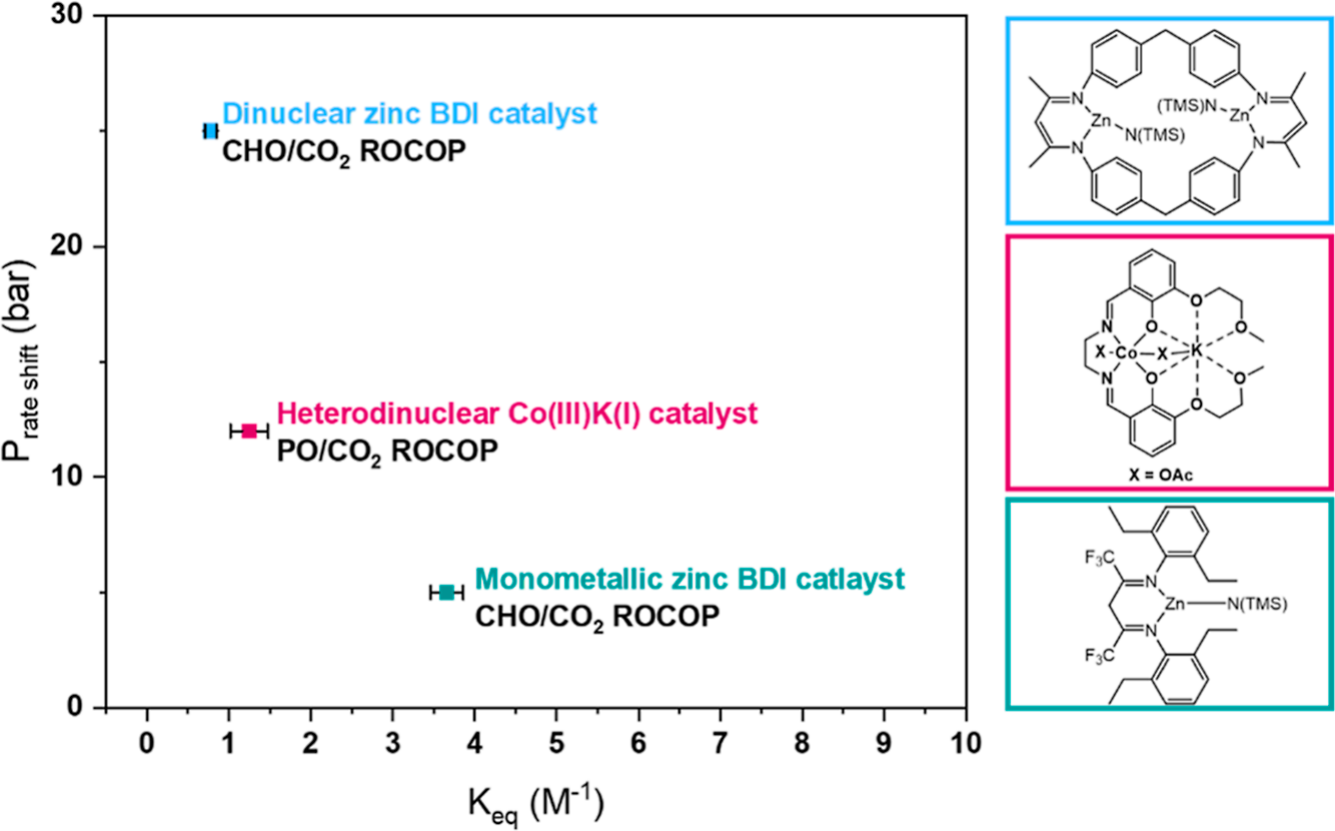
Correlation between the equilibrium constant (*K*_eq_) estimated in this work and the pressure at which a shift in rate was observed for each catalyst.^28,51^

Using the new kinetic methods allows for the estimation of the equilibrium constants for both PO/CO_2_ ROCOP catalyst **1** and two previously reported Zn(II) CHO/CO_2_ ROCOP catalysts (Figure 11). It must be emphasized that CHO/CO_2_ ROCOP is both more widely examined and more easily reanalyzed since its barrier to cyclic carbonate formation is usually substantially higher than that for polymerization (i.e., selectivity for polymer is quantitative, which simplifies the equilibrium constant quantification).^5,12^ Within these three catalysts, the zinc β-diiminate complex has the highest *K*_eq_ (3.7 M^–1^) and the lowest pressure-dependent rate saturation (5–10 bar of CO_2_). In contrast, the equilibrium constants for the Co(III)K(I) catalyst **1** and the di-Zn(II) macrocycle catalyst (for CHO/CO_2_ ROCOP), are lower at 1.27 and 0.78 M^–1^, respectively. These two catalysts also show rate saturation (vs pressure) at higher carbon dioxide pressures of 12 and 25 bar, respectively. The correlation between *K*_eq_ and the saturation rate pressure may suggest that catalysts with larger carbon dioxide insertion equilibrium constants perform more consistently at lower CO_2_ pressures (Figure 11). This hypothesis remains tentative due to the limited data available in the literature but may help guide the approximate magnitude of the equilibrium constant for the best catalytic activity. As such, for PO/CO_2_ ROCOP catalysts to show activity below 5 bar, the *K*_eq_ should be ≥1.27 M^–1^ at 50 °C. For CHO/CO_2_ ROCOP catalysts to show activity below 5 bar, the *K*_eq_ should be ≥3.7 M^–1^.

The quantification of carbon dioxide insertion equilibrium constants and their use in rate laws are recommended in future investigations of new catalysts. Further, such catalysts should be explicitly designed to drive the equilibria toward carbonate intermediates. There are still improvements to be made in this field, and this work reveals that the best catalysts will show high equilibrium constants such that the catalyst concentration equates with the carbonate intermediate, regardless of the applied pressure and temperature. Such catalysts will be suitable for use under conditions that minimize energy input (low gas pressure) and maximize rates (higher temperature).

The importance of similar CO_2_ insertion equilibria has been previously reported for the cycloaddition of CO_2_ to epoxides, forming cyclic carbonates.^52^ More generally, catalyst–substrate equilibria are, of course, very well-known in other fields, perhaps most famously in enzyme kinetics and biochemistry, where Michaelis–Menten kinetic treatments are highly successful.^48,53−55^ We propose that in future, the quantification of carbon dioxide *K*_eq_ and epoxide/CO_2_*k*_cat_ may improve comparisons between different catalysts. These parameters are more informative and robust for comparisons compared with the metrics that have traditionally dominated the field, i.e., turnover frequency (TOF) or pseudo-order rate coefficients (*k*_obs_) which are only relevant under specific conditions. Identifying reliable methods to compare catalysts is especially important in the context of accelerating process development to reduce GHG emissions.

## Conclusions

A series of highly active and selective heterodinuclear Co(III)M(I) carbon dioxide/PO polymerization catalysts were reported. The best catalyst, Co(III)K(I) (**1**), achieved an activity of 1050 h^–1^, >97% polymer selectivity, and operated at 1:10,000 loading (0.001 mol %). It retained its high activity and selectivity at both low pressures (2–12 bar) and temperatures (50–80 °C). The polymerization rates were influenced by catalyst, epoxide, and carbon dioxide concentrations at both low (5 bar) and high (20 bar) pressures. A unified rate law, applicable under all conditions, was presented which accounts for the extent of the carbon dioxide insertion equilibrium. The CO_2_ insertion equilibrium constant was quantified for the first time, and the barriers to both polymer and cyclic carbonate byproduct formation were compared with other leading catalysts. The unified rate law and CO_2_ pre-equilibrium insertion chemistry are expected to be generally applicable, including to many other catalysts and monomers. As a proof of potential, the methods described in this work were retrospectively applied to previously published high-performance catalysts, allowing the quantification of the carbon dioxide insertion equilibrium constants, which were quantified, and the rationalization of the experimental data. In the future, these unified kinetic methods and insights into equilibria should underpin both catalyst and polymerization process design to maximize efficiency and minimize energy inputs.

## Supporting Information Available

Characterization data of catalysts (1D and 2D NMR spectra, infrared spectra, cyclic voltammetry spectra, and X-ray crystallography); polymerization kinetics; COPASI modeling details; and extrapolation of CO_2_ concentration data in PO (PDF)
Spreadsheet detailing the calculation of *K*_eq_ (XLSX)

## Abbreviations

PO: propene oxide
CHO: cyclohexane oxide
PPC: poly(propene carbonate)
PC: propene carbonate
RDS: rate-determining step

## References

[ref1] Agency I. E. Putting CO2 to Use, Paris 2019. https://www.iea.org/reports/putting-co2-to-use (accessed July 30, 2023).

[ref2] Artz J.; Müller T. E.; Thenert K.; Kleinekorte J.; Meys R.; Sternberg A.; Bardow A.; Leitner W. Sustainable Conversion of Carbon Dioxide: An Integrated Review of Catalysis and Life Cycle Assessment. Chem. Rev. 2018, 118 (2), 434–504. 10.1021/acs.chemrev.7b00435. 29220170

[ref3] Burkart M. D.; Hazari N.; Tway C. L.; Zeitler E. L. Opportunities and Challenges for Catalysis in Carbon Dioxide Utilization. ACS Catal. 2019, 9 (9), 7937–7956. 10.1021/acscatal.9b02113.

[ref4] Hepburn C.; Adlen E.; Beddington J.; Carter E. A.; Fuss S.; Mac Dowell N.; Minx J. C.; Smith P.; Williams C. K. The technological and economic prospects for CO2 utilization and removal. Nature 2019, 575 (7781), 87–97. 10.1038/s41586-019-1681-6. 31695213

[ref5] Bhat G. A.; Darensbourg D. J. Coordination complexes as catalysts for the coupling reactions of oxiranes and carbon dioxide. Coord. Chem. Rev. 2023, 492, 215277 10.1016/j.ccr.2023.215277.

[ref6] Liu Y.; Lu X.-B. Current Challenges and Perspectives in CO2-Based Polymers. Macromolecules 2023, 56 (5), 1759–1777. 10.1021/acs.macromol.2c02483.

[ref7] Langanke J.; Wolf A.; Hofmann J.; Böhm K.; Subhani M. A.; Müller T. E.; Leitner W.; Gürtler C. Carbon dioxide (CO_2_) as sustainable feedstock for polyurethane production. Green Chem. 2014, 16 (4), 1865–1870. 10.1039/C3GC41788C.

[ref8] Alagi P.; Ghorpade R.; Choi Y. J.; Patil U.; Kim I.; Baik J. H.; Hong S. C. Carbon Dioxide-Based Polyols as Sustainable Feedstock of Thermoplastic Polyurethane for Corrosion-Resistant Metal Coating. ACS Sustainable Chem. Eng. 2017, 5 (5), 3871–3881. 10.1021/acssuschemeng.6b03046.

[ref9] Kuang Q.; Zhang R.; Zhou Z.; Liao C.; Liu S.; Chen X.; Wang X. A Supported Catalyst that Enables the Synthesis of Colorless CO_2_-Polyols with Ultra-Low Molecular Weight. Angew. Chem., Int. Ed. 2023, 62 (35), e202305186 10.1002/anie.202305186. 37157011

[ref10] Chemanalyst. Propylene Oxide Market Analysis, 2015–2032. 2023. https://www.chemanalyst.com/industry-report/propylene-oxide-po-market-755 (accessed Nov 30, 2023).

[ref11] Deacy A. C.; Phanopoulos A.; Lindeboom W.; Buchard A.; Williams C. K. Insights into the Mechanism of Carbon Dioxide and Propylene Oxide Ring-Opening Copolymerization Using a Co(III)/K(I) Heterodinuclear Catalyst. J. Am. Chem. Soc. 2022, 144 (39), 17929–17938. 10.1021/jacs.2c06921. 36130075 PMC9545154

[ref12] Darensbourg D. J.; Yarbrough J. C.; Ortiz C.; Fang C. C. Comparative Kinetic Studies of the Copolymerization of Cyclohexene Oxide and Propylene Oxide with Carbon Dioxide in the Presence of Chromium Salen Derivatives. In Situ FTIR Measurements of Copolymer vs Cyclic Carbonate Production. J. Am. Chem. Soc. 2003, 125 (25), 7586–7591. 10.1021/ja034863e. 12812499

[ref13] Roznowska A.; Dyduch K.; Lee B. Y.; Michalak A. Theoretical study on preference of open polymer vs. cyclic products in CO_2_/epoxide copolymerization with cobalt(III)-salen bifunctional catalysts. J. Mol. Model. 2020, 26 (6), 113 10.1007/s00894-020-04364-x. 32378131 PMC7203596

[ref14] Lidston C. A. L.; Severson S. M.; Abel B. A.; Coates G. W. Multifunctional Catalysts for Ring-Opening Copolymerizations. ACS Catal. 2022, 12 (18), 11037–11070. 10.1021/acscatal.2c02524.

[ref15] Diment W. T.; Lindeboom W.; Fiorentini F.; Deacy A. C.; Williams C. K. Synergic Heterodinuclear Catalysts for the Ring-Opening Copolymerization (ROCOP) of Epoxides, Carbon Dioxide, and Anhydrides. Acc. Chem. Res. 2022, 55 (15), 1997–2010. 10.1021/acs.accounts.2c00197. 35863044 PMC9350912

[ref16] Grignard B.; Gennen S.; Jérôme C.; Kleij A. W.; Detrembleur C. Advances in the use of CO2 as a renewable feedstock for the synthesis of polymers. Chem. Soc. Rev. 2019, 48 (16), 4466–4514. 10.1039/C9CS00047J. 31276137

[ref17] Yang G.-W.; Xu C.-K.; Xie R.; Zhang Y.-Y.; Lu C.; Qi H.; Yang L.; Wang Y.; Wu G.-P. Precision copolymerization of CO2 and epoxides enabled by organoboron catalysts. Nat. Synth. 2022, 1 (11), 892–901. 10.1038/s44160-022-00137-x.

[ref18] Zhou Z.; Liu S.; Yang L.; Kuang Q.; Zhou H.; Zhuo C.; Wang X. Dynamic Foldamer Catalyst Enables Efficient Copolymerization of CO2 and Epoxides. ACS Catal. 2023, 13 (22), 15116–15125. 10.1021/acscatal.3c04274.

[ref19] Zhang Y.-Y.; Yang G.-W.; Xie R.; Zhu X.-F.; Wu G.-P. Sequence-Reversible Construction of Oxygen-Rich Block Copolymers from Epoxide Mixtures by Organoboron Catalysts. J. Am. Chem. Soc. 2022, 144 (43), 19896–19909. 10.1021/jacs.2c07857. 36256447

[ref20] Wang Y.; Liu Z.; Guo W.; Zhang C.; Zhang X. Phosphine-Borane Frustrated Lewis Pairs for Metal-Free CO2/Epoxide Copolymerization. Macromolecules 2023, 56, 4901–4909. 10.1021/acs.macromol.3c00941.

[ref21] Nagae H.; Matsushiro S.; Okuda J.; Mashima K. Cationic tetranuclear macrocyclic CaCo3 complexes as highly active catalysts for alternating copolymerization of propylene oxide and carbon dioxide. Chem. Sci. 2023, 14, 8262–8268. 10.1039/D3SC00974B. 37564411 PMC10411860

[ref22] Cohen C. T.; Chu T.; Coates G. W. Cobalt Catalysts for the Alternating Copolymerization of Propylene Oxide and Carbon Dioxide: Combining High Activity and Selectivity. J. Am. Chem. Soc. 2005, 127 (31), 10869–10878. 10.1021/ja051744l. 16076192

[ref23] Cyriac A.; Lee S. H.; Varghese J. K.; Park E. S.; Park J. H.; Lee B. Y. Immortal CO2/Propylene Oxide Copolymerization: Precise Control of Molecular Weight and Architecture of Various Block Copolymers. Macromolecules 2010, 43 (18), 7398–7401. 10.1021/ma101259k.

[ref24] Deng J.; Ratanasak M.; Sako Y.; Tokuda H.; Maeda C.; Hasegawa J.-y.; Nozaki K.; Ema T. Aluminum porphyrins with quaternary ammonium halides as catalysts for copolymerization of cyclohexene oxide and CO2: metal-ligand cooperative catalysis. Chem. Sci. 2020, 11 (22), 5669–5675. 10.1039/D0SC01609H. 32864082 PMC7425082

[ref25] Yang G.-W.; Zhang Y.-Y.; Wu G.-P. Modular Organoboron Catalysts Enable Transformations with Unprecedented Reactivity. Acc. Chem. Res. 2021, 54 (23), 4434–4448. 10.1021/acs.accounts.1c00620. 34806374

[ref26] Chen C.; Gnanou Y.; Feng X. Ultra-Productive Upcycling CO2 into Polycarbonate Polyols via Borinane-Based Bifunctional Organocatalysts. Macromolecules 2023, 56 (3), 892–898. 10.1021/acs.macromol.2c02243.

[ref27] Chen C.; Gnanou Y.; Feng X. Borinane-based organoboron catalysts for alternating copolymerization of CO2 with cyclic ethers: improved productivity and facile recovery. Polym. Chem. 2022, 13 (45), 6312–6321. 10.1039/D2PY01161A.

[ref28] Lehenmeier M. W.; Kissling S.; Altenbuchner P. T.; Bruckmeier C.; Deglmann P.; Brym A.-K.; Rieger B. Flexibly Tethered Dinuclear Zinc Complexes: A Solution to the Entropy Problem in CO2/Epoxide Copolymerization Catalysis?. Angew. Chem., Int. Ed. 2013, 52 (37), 9821–9826. 10.1002/anie.201302157. 23873829

[ref29] Darensbourg D. J.; Wildeson J. R.; Yarbrough J. C.; Reibenspies J. H. Bis 2,6-difluorophenoxide Dimeric Complexes of Zinc and Cadmium and Their Phosphine Adducts: Lessons Learned Relative to Carbon Dioxide/Cyclohexene Oxide Alternating Copolymerization Processes Catalyzed by Zinc Phenoxides. J. Am. Chem. Soc. 2000, 122 (50), 12487–12496. 10.1021/ja002855h.

[ref30] Moore D. R.; Cheng M.; Lobkovsky E. B.; Coates G. W. Mechanism of the Alternating Copolymerization of Epoxides and CO_2_ Using β-Diiminate Zinc Catalysts: Evidence for a Bimetallic Epoxide Enchainment. J. Am. Chem. Soc. 2003, 125 (39), 11911–11924. 10.1021/ja030085e. 14505413

[ref31] Deacy A. C.; Moreby E.; Phanopoulos A.; Williams C. K. Co(III)/Alkali-Metal(I) Heterodinuclear Catalysts for the Ring-Opening Copolymerization of CO_2_ and Propylene Oxide. J. Am. Chem. Soc. 2020, 142 (45), 19150–19160. 10.1021/jacs.0c07980. 33108736 PMC7662907

[ref32] Duan R.; Hu C.; Sun Z.; Zhang H.; Pang X.; Chen X. Conjugated tri-nuclear salen-Co complexes for the copolymerization of epoxides/CO_2_: cocatalyst-free catalysis. Green Chem. 2019, 21 (17), 4723–4731. 10.1039/C9GC02045D.

[ref33] Darensbourg D. J.; Holtcamp M. W.; Struck G. E.; Zimmer M. S.; Niezgoda S. A.; Rainey P.; Robertson J. B.; Draper J. D.; Reibenspies J. H. Catalytic Activity of a Series of Zn(II) Phenoxides for the Copolymerization of Epoxides and Carbon Dioxide. J. Am. Chem. Soc. 1999, 121 (1), 107–116. 10.1021/ja9826284.

[ref34] Wu W.; Qin Y.; Wang X.; Wang F. New bifunctional catalyst based on cobalt-porphyrin complex for the copolymerization of propylene oxide and CO_2_. J. Polym. Sci., Part A: Polym. Chem. 2013, 51 (3), 493–498. 10.1002/pola.26434.

[ref35] Chapman A. M.; Keyworth C.; Kember M. R.; Lennox A. J. J.; Williams C. K. Adding Value to Power Station Captured CO_2_: Tolerant Zn and Mg Homogeneous Catalysts for Polycarbonate Polyol Production. ACS Catal. 2015, 5 (3), 1581–1588. 10.1021/cs501798s.

[ref36] von der Assen N.; Bardow A. Life cycle assessment of polyols for polyurethane production using CO_2_ as feedstock: insights from an industrial case study. Green Chem. 2014, 16 (6), 3272–3280. 10.1039/C4GC00513A.

[ref37] Deacy A. C.; Durr C. B.; Kerr R. W. F.; Williams C. K. Heterodinuclear catalysts Zn(ii)/M and Mg(ii)/M, where M = Na(i), Ca(ii) or Cd(ii), for phthalic anhydride/cyclohexene oxide ring opening copolymerisation. Catal. Sci. Technol. 2021, 11 (9), 3109–3118. 10.1039/D1CY00238D.

[ref38] Deacy A. C.; Kilpatrick A. F. R.; Regoutz A.; Williams C. K. Understanding metal synergy in heterodinuclear catalysts for the copolymerization of CO2 and epoxides. Nat. Chem. 2020, 12 (4), 372–380. 10.1038/s41557-020-0450-3. 32221501

[ref39] Yang G.-W.; Xu C.-K.; Xie R.; Zhang Y.-Y.; Zhu X.-F.; Wu G.-P. Pinwheel-Shaped Tetranuclear Organoboron Catalysts for Perfectly Alternating Copolymerization of CO_2_ and Epichlorohydrin. J. Am. Chem. Soc. 2021, 143 (9), 3455–3465. 10.1021/jacs.0c12425. 33591738

[ref40] Lindeboom W.; Deacy A. C.; Phanopoulos A.; Buchard A.; Williams C. K. Correlating Metal Redox Potentials to Co(III)K(I) Catalyst Performances in Carbon Dioxide and Propene Oxide Ring Opening Copolymerization. Angew. Chem., Int. Ed. 2023, 62 (37), e202308378 10.1002/anie.202308378. PMC10952574 37409487

[ref41] Lu X.-B.; Shi L.; Wang Y.-M.; Zhang R.; Zhang Y.-J.; Peng X.-J.; Zhang Z.-C.; Li B. Design of Highly Active Binary Catalyst Systems for CO_2_/Epoxide Copolymerization: Polymer Selectivity, Enantioselectivity, and Stereochemistry Control. J. Am. Chem. Soc. 2006, 128 (5), 1664–1674. 10.1021/ja056383o. 16448140

[ref42] Fiorentini F.; Diment W. T.; Deacy A. C.; Kerr R. W. F.; Faulkner S.; Williams C. K. Understanding catalytic synergy in dinuclear polymerization catalysts for sustainable polymers. Nat. Commun. 2023, 14 (1), 4783 10.1038/s41467-023-40284-z. 37553344 PMC10409799

[ref43] Schall O. F.; Robinson K.; Atwood J. L.; Gokel G. W. Self-assembling nickel clusters form binding sites for alkali metal cations: novel analogs of enolate aggregates. J. Am. Chem. Soc. 1993, 115 (14), 5962–5969. 10.1021/ja00067a010.

[ref44] Trott G.; Garden J. A.; Williams C. K. Heterodinuclear zinc and magnesium catalysts for epoxide/CO2 ring opening copolymerizations. Chem. Sci. 2019, 10 (17), 4618–4627. 10.1039/C9SC00385A. 31123572 PMC6492632

[ref45] Shannon R. D. Revised effective ionic radii and systematic studies of interatomic distances in halides and chalcogenides. Acta Cryst. A 1976, 32 (5), 751–767. 10.1107/S0567739476001551.

[ref46] Foltran S.; Cloutet E.; Cramail H.; Tassaing T. In situ FTIR investigation of the solubility and swelling of model epoxides in supercritical CO_2_. J. Supercrit. Fluids 2012, 63, 52–58. 10.1016/j.supflu.2011.12.015.

[ref47] Hoops S.; Sahle S.; Gauges R.; Lee C.; Pahle J.; Simus N.; Singhal M.; Xu L.; Mendes P.; Kummer U. COPASI—a Complex Pathway Simulator. Bioinformatics 2006, 22 (24), 3067–3074. 10.1093/bioinformatics/btl485. 17032683

[ref48] Atkins P.; De Paula J.; Keeler J. Atkins’ Physical Chemistry; Oxford University Press, 2017.

[ref49] Deacy A. C.; Phanopoulos A.; Lindeboom W.; Buchard A.; Williams C. K. Insights into the Mechanism of Carbon Dioxide and Propylene Oxide Ring-Opening Copolymerization Using a Co(III)/K(I) Heterodinuclear Catalyst. J. Am. Chem. Soc. 2022, 144 (39), 17929–17938. 10.1021/jacs.2c06921. 36130075 PMC9545154

[ref50] Kernbichl S.; Reiter M.; Mock J.; Rieger B. Terpolymerization of β-Butyrolactone, Epoxides, and CO_2_: Chemoselective CO_2_-Switch and Its Impact on Kinetics and Material Properties. Macromolecules 2019, 52 (21), 8476–8483. 10.1021/acs.macromol.9b01777.

[ref51] Reiter M.; Vagin S.; Kronast A.; Jandl C.; Rieger B. A Lewis acid β-diiminato-zinc-complex as all-rounder for co- and terpolymerisation of various epoxides with carbon dioxide. Chem. Sci. 2017, 8 (3), 1876–1882. 10.1039/C6SC04477H. 28567266 PMC5444112

[ref52] D’Elia V.; Ghani A. A.; Monassier A.; Sofack-Kreutzer J.; Pelletier J. D. A.; Drees M.; Vummaleti S. V. C.; Poater A.; Cavallo L.; Cokoja M.; et al. Dynamics of the NbCl5-Catalyzed Cycloaddition of Propylene Oxide and CO2: Assessing the Dual Role of the Nucleophilic Co-Catalysts. Chem.—Eur. J. 2014, 20 (37), 11870–11882. 10.1002/chem.201400324. 25056457

[ref53] Cornish-Bowden A. One hundred years of Michaelis-Menten kinetics. Sci. Perspect. 2015, 4, 3–9. 10.1016/j.pisc.2014.12.002.

[ref54] Michaelis L.; Menten M. L. Die Kinetik der Invertinwirkung. Biochem. Z. 1913, 49 (333–369), 352.

[ref55] Nelson D. L.; Cox M. M.; Hoskins A. A. Lehninger Principles of Biochemistry; Macmillan Learning: New York, NY, 2021.

