# Supplementary material for: Quantifying CO2 Insertion Equilibria for Low-Pressure Propene Oxide and Carbon Dioxide Ring Opening Copolymerization Catalysts
Source: J Am Chem Soc. 2024 Apr 8;146(15):10451–64. doi: 10.1021/jacs.3c13959 (PMC11027146; doi:10.1021/jacs.3c13959)
Supplement: Supplementary file 1 — ja3c13959_si_001.pdf [file ja3c13959_si_001.pdf]

Supporting Information

Quantifying CO_2 Insertion Equilibria For Low Pressure Propene Oxide and Carbon Dioxide Ring Opening Copolymerization Catalysts

Katharina H. S. Eisenhardt, Francesca Fiorentini, Wouter Lindeboom, Charlotte K. Williams*

Department Chemistry, University of Oxford, Chemistry Research Laboratory, 12 Mansfield Road, Oxford, OX1 3TA UK.

Table of Contents

Materials **5**

NMR analysis 5

FT-IR measurements 5

Gel permeation chromatography (GPC) 5

Cyclic Voltammetry (CV) 5

Mass Spectrometry 5

Elemental analysis 5

Methods **6**

General epoxide/CO_2 polymerisation procedure 6

Synthesis of 2-hydroxy-3-(2-methoxyethoxy)benzaldehyde pro-ligand 6

'Open ligand' synthesis (H_2L_1) 6

Co(III)K(I) 'open' complex (1) synthesis 7

Co(III)Na (I) 'open' complex (2) synthesis 7

Co(III)Rb(I) 'open' complex (3) synthesis 7

Additional Information **8**

Fig. S1 ^{1}H NMR spectrum of 2-hydroxy-3-(2-methoxyethoxy)benzaldehyde pro-ligand in $CDCl_3$. 8

Fig. S2 ^{13}C NMR spectrum of the 2-hydroxy-3-(2-methoxyethoxy)benzaldehyde pro-ligand in $CDCl_3$. 9

Fig. S3 COSY NMR spectrum of the 2-hydroxy-3-(2-methoxyethoxy)benzaldehyde pro-ligand in $CDCl_3$. 9

Fig. S4 HSQC NMR spectrum of 2-hydroxy-3-(2-methoxyethoxy)benzaldehyde pro-ligand in $CDCl_3$. 10

Fig. S5 HMBC NMR spectrum of 2-hydroxy-3-(2-methoxyethoxy)benzaldehyde pro-ligand in $CDCl_3$. 10

Fig. S6 ^{1}H NMR spectrum of H_1L_2 in $CDCl_3$. 11

Fig. S7 ^{13}C NMR spectrum of H_1L_2 in $CDCl_3$. 11

Fig. S8 COSY NMR spectrum of H_1L_2 in $CDCl_3$. 12

Fig. S9 HSQC spectrum of H_1L_2 in $CDCl_3$. 12

Fig. S10 HMBC spectrum of H_1L_2 in $CDCl_3$. 13

Fig. S11 FT-IR spectra of H_2L_1, Co(III)K(I) (1), Co(III)Na(I) (2) and Co(III)Rb(I) (3). 13

Fig. S12 ^{1}H NMR spectrum of (1) in $CDCl_3$. 14

Fig. S13 ^{13}C NMR spectrum of (1) in $CDCl_3$. 14

Fig S14 COSY NMR spectrum of (1) in $CDCl_3$. 15

Fig. S15 HSQC NMR spectrum of (1) in $CDCl_3$. 15

Fig. S16 HMBC NMR spectrum of (1) in $CDCl_3$. 16

Fig. S17 ^{1}H NMR spectrum of (2) in $CDCl_3$. 16

Fig. S18 ^{13}C NMR spectrum of (2) in $CDCl_3$. 17

Fig S19 COSY NMR spectrum of (2) in $CDCl_3$. 17
Fig. S20 HSQC NMR spectrum of (2) in $CDCl_3$. 18
Fig. S21 HMBC spectrum of (2) in $CDCl_3$. 18
Fig. S22 ^{1}H NMR spectrum of (3) in $CDCl_3$. 19
Fig. S23 ^{13}C NMR spectrum of (3) in $CDCl_3$. 19
Fig. S24 COSY NMR spectrum of (3) in $CDCl_3$. 20
Fig. S25 HSQC NMR spectrum of (3) in $CDCl_3$. 20
Fig. S26 HMBC NMR spectrum of (3) in $CDCl_3$.] 21
Fig. S27 CV graphs of complexes a) Co(III) Na(I), b) Co(III)K(I), c) Co(III)Rb(I). 21
Fig. S28 Overview of single crystal XRD structures. 22
Tab. S1 Experimental values used to calculated averages in Table 2[a] 23
Fig. S29 Monomodal GPC trace for high weight PPC, synthesised using 1 (Tab. 2, entry 10). 24
Tab. S2 Depolymerisation of PPC using 1[a] 24
Interpolation of [CO_2] (M) from previously reported data 25
Tab. S3 [CO_2] concentration in PO with varying CO_2 pressures at 40 °C, data supplied by Foltran et al.[2] 25
Tab. S4 [CO_2] concentration in PO with varying CO_2 pressures at 70 °C, data supplied by Foltran et al.[2] 25
Tab. S5 [CO_2] concentration in PO with varying CO_2 pressures at 100 °C, data supplied by Foltran et al.[2] 26
Fig. S30 Plots of [CO_2] (M) vs CO_2 pressure (bar) at (a) 40°C, (b) 70 °C and (c) 100 °C, with fitted trendlines. 26
Tab. S6 CO_2 concentration at pressures between 2 and 30 bar at 40°C, 70 °C and 100 °C. The shown data was obtained by extrapolation from graphs shown in Fig. S5. 27
Fig. S31 Plot of [CO_2] (M) vs temperature at P = 2-30 bar. Data taken from Tab. S6, exponential decay trendlines fitted for each pressure. 27
Tab. S7 [CO_2] (M) at pressures between 2 and 30 bar, obtained by extrapolation of data shown in Fig. S6. 28
Fig. S32 Linear increase of [CO_2] with CO_2 pressure at 50°C. Data obtained by interpolation as outlined in Tab. S3-7. 28
Kinetic Data 29
Tab. S8 Dependence of Activity and Selectivity on CO_2 pressure and CO_2 concentration. 29
Fig. S33 a) ln(k_{obs}) vs ln(CO_2) b) ln(k_{obs}) vs ln(P_{CO2}). 30
Tab. S9 Dependence of Activity and Selectivity on catalyst concentration at 5 bar CO_2 pressure.[a] 30
Tab. S10 Dependence of Activity and Selectivity on catalyst concentration at 20 bar CO_2 pressure.[a] 31
Tab. S11 Dependence of Activity and Selectivity on PO concentration at 5 bar. 31
Tab. S12 Dependence of Activity and Selectivity on PO concentration at 20 bar.[a] 32
Fig. S34 a) ln(k_{obs}) vs ln([cat]) at 5 bar b) ln(k_{obs}) vs ln([cat]) at 20 bar 32

Fig. S35 Semilogarithmic plots for ln([PO]/[PO]$_0$) vs time for all tested PO concentrations at 5 bar CO_2 pressure. 33

Fig. S36 Semilogarithmic plots for ln([PO]/[PO]$_0$) vs time for all tested PO concentrations at 20 bar CO_2 pressure. 33

Fig. S37 VTNA plot showing the best fit for an order of (b) 1 at 5 bar, and inferior fits for orders of (a) 0 and (c) 2. 34

Fig. S38 VTNA plot showing the best fit for an order of 1 at 20 bar (b), and inferior fits for an order of (a) 0 (c) 2 34

Fig. S39 Graphs illustrating inferior fits for a COPASI model when a) removing CO_2 dependence for the rate law at 5 bar 34

Table 13 Data showing how varying the pressure and [CO_2] impacts the rate coefficient (k_{obs}), [carbonate], [alkoxide] and K_{eq} according to the unified rate law. 35

Fig. S40 Comparison of the three field-leading PO/CO_2 ROCOP catalysts: cobalt salen[5], tethered cobalt salen[6] and 1. 35

Backbiting mechanism and barrier determination 36

Fig. S41 a) Polymerization mechanism and backbiting mechanism from the alkoxide b) Backbiting barrier (mechanism) determination using hydroxyl-end capped PPC to form cyclic carbonate 36

Fig. S42 Linear correlation of the [alkoxide] : [carbonate] ratio with the PC:PPC product ratio 37

Figure S43 Determination of PPC regiochemistry (with assignments illustrated) using quantitative ^{13}C NMR spectroscopy. 38

Estimation of K_{eq} of previously reported Zinc BDI complexes 39

Fig. S44 Structure and rate laws reported for a) Mononuclear Zinc BDI catalyst for the CHO/CO_2 ROCOP[8] and b) Dinuclear zinc BDI catalyst for the CHO/CO_2 ROCOP[9]. 39

Fig. S45 Proposed mechanisms for the previously reported dinuclear zinc BDI CHO/CO_2 ROCOP catalyst, ... 40

Fig. S46 Proposed mechanisms for the previously reported monometallic zinc BDI CHO/CO_2 ROCOP catalyst, ... 41

COPASI Modelling 42

Fig. S47 Illustration of (a) the Pre-equilibrium approximation and (b) the Steady-State approximation assumed for the PO/CO_2 ROCOP using 1 at low $P < 12$ bar and $P > 12$ bar, respectively. 42

Tab. S14 Parameters and rate equations used for COPASI models. 43

Fig. S48 Concentration vs time data modelled using a rate law without a CO_2 dependence at low pressure and a first order CO_2 dependence at high pressure in COPASI and comparison with experimentally collected concentration vs time data for reactions performed 44

Single X-Ray Crystallography 45

Tab. S15 Selected bond lengths for Co(III)K(I) (1), Co(III)Na(I) (2) and Co(III)Rb(I) (3). 45

Tab. S16 Selected angles for Co(III)K(I) (1), Co(III)Na(I) (2) and Co(III)Rb(I) (3). 46

Tab. S17 Summary of crystallographic refinement data for complexes 1 – 3. 47

Materials

All experimental manipulations were performed using a dual-manifold nitrogen-vacuum Schlenk line or in a nitrogen filled glovebox. All solvents and reagents were obtained from commercial sources and used as received, unless stated otherwise. Acetonitrile, pentane, toluene and THF were obtained from an SPS system, degassed by several freeze-pump-thaw cycles, further dried with 3 Å molecular sieves and stored under N_2. Anhydrous DMSO was stored over molecular sieves for 7 days prior to use. PO was dried over two nights over calcium hydride and purified by fractional distillation, followed by degassing with N_2 and stored under N_2. Research-grade CO_2 (BOC, CP grade, 99.995%) was dried by passing it through two drying columns (VICI Metronics carbon dioxide purifier) in series, at 50 bar pressure, before use at lower pressures in the copolymerizations.

NMR analysis were performed using a Bruker AV 400 MHz spectrometer, at 298 K, unless stated.

FT-IR measurements were performed using a Shimadzu IRSpirit spectrometer (installed inside the glove box) using a single reflection ATR accessory**.**

Gel permeation chromatography (GPC) was performed using a Shimadzu LC-20AD instrument with two mixed bed PSS SDV linear S columns in series at 40 °C. THF was used as eluent at a flow rate of 1 mL/min. Molar mass calibration was performed using a narrow molar mass polystyrene standard. Samples labelled with an asterisk (*) were analysed using a N Agilent LC1260 Infinity II System fitted with a PLgel 5 μm (50 x 7.5 mm) guard column and two PLgel 5 μm MIXED-C (300 x 7.5 mm) analytical columns and equipped with a multi-detector suite (MDS) comprising a dual-angle light scattering detector (LS, 15° & 90°), refractive index detector (RI), and viscometer (VS). THF (FisherScientific, GPC grade stabilized with 0.025% BHT) was used as the eluent with a flow rate of 1 mL/min at 30 ^{o}C. The system was calibrated using a set of narrow polystyrene standards (Agilent EasiVial PS-H 2 mL) for standard GPC calibration and a narrow polystyrene standard (M_p = 29,510 g/mol, $M_{w,LS}$ = 29,810 g/mol, dn/dc = 0.185, M_w/M_n = 1.02, [η] = 0.1777 dL/g) for universal calibration.

Cyclic Voltammetry (CV) was carried out using a PalmSens EmStat Blue potentiostat. Cyclic voltammetry experiments were performed in a N_2 glovebox using a three-electrode configuration, with an Au disc (2.0 mm^2) as the working electrode, a glassy carbon electrode (2.0 mm^2) as the counter electrode and an Ag wire as the pseudo-reference electrode. Experiments were performed using a sample solution, containing 0.1 M of tetrabutylammonium hexafluorophosphate as supporting electrolyte and the analyte (ca. 5mM) in dry, degassed acetonitrile. Experiments were performed using a 100 mV s^{-1} scan rate, unless otherwise state. Ferrocene (ca. 1 mg) was added to the sample solution as at the end of the experiment and the measured redox potential was used as internal standard.

Mass Spectrometry was performed using a Thermo Exactive High-Resolution Orbitrap FTMS without front end LC in direct infusion (loop injection) mode.

Elemental analysis was carried out by the London Metropolitan University (166-220 Holloway Road, London, N7 8DB).

Methods

General epoxide/CO_2 polymerisation procedure: In a nitrogen-filled glovebox, a solution of catalyst, *trans*-1,2-cyclohexene diol and mesitylene (internal standard) in neat epoxide was prepared. The solution was then injected into a 100 mL Parr reactor, fitted with a DiComp sentinel probe, attached to an ATR-IR spectrometer, under a stream of dry CO_2. For reactions performed under static CO_2, the reactor was then pressurised with CO_2 to the target reaction pressure and allowed to reach the required temperature. The reactor was then heated to the target temperature. For all kinetic experiments, pressurisation was controlled using a Bronkhorst EP-Flow select Mass Flow Meter. The reaction was left open to the CO_2 line throughout the experiment and the pressure was kept constant through automated CO_2 injections by the Flow Meter. The reaction was monitored by following increase in polymer IR signal at 1750 cm^{-1}. Upon reaction completion, the reactor vessel was cooled to room temperature and depressurized. The catalyst was quenched by the addition of a 1 M solution of benzoic acid, in $CHCl_3$. A sample of the crude reaction mixture was removed for NMR analysis and GPC analysis.

General depolymerisation procedure: A 100 mL Parr reactor vessel, fitted with a DiComp sentinel probe, attached to an ATR-IR spectrometer was charged with catalyst **1** (11 mg, 0.018 mmol) as a solid, followed by a solution of PPC (0.184 g, 1.8 mmol) in PO (6 mL, 86 mmol) and the reaction mixture was heated to the target temperature. The reaction progression was followed by monitoring the polymer IR signal and the propylene carbonate IR signal, at 1750 cm^{-1} and 1820 cm^{-1}, respectively. Upon full disappearance of the polymer signal, the reactor vessel was cooled to room temperature. The reaction mixture was quenched using a 1 M solution of benzoic acid, in $CHCl_{3.}$ Subsequently, a sample of the crude reaction mixture was removed for NMR analysis.

Synthesis of 2-hydroxy-3-(2-methoxyethoxy)benzaldehyde pro-ligand

The pro-ligand was prepared by a modified literature procedure.[1] A solution of dried 2,3-dihydroxybenzaldehyde (5 g, 35.09 mmol) in DMSO was added dropwise to an ice cooled, stirred suspension of NaH (1.95 g, 2.1 mmol) in DMSO, under a N_2 atmosphere. The solution was left to warm to room temperature. After stirring the solution for 1 h, at room temperature, degassed 2-methoxyethoxy-p-toluensulfonate (7.29 mL, 39.60 mmol) was added in one portion. The red solution was left to stir for 72 hours at room temperature. The reaction was quenched by carefully adding water (150 mL). The aqueous layer was subsequently extracted with $CHCl_3$ (3 x 75 mL) and subsequently acidified to pH 2 using 1 M HCl (25 mL). The colour of the solution changed from dark red to yellow upon acidification. The aqueous layer was then extracted with $CHCl_3$ (3 x 75 mL) and the organic layer washed with HCl (3 x 50 mL) and dried ($MgSO_4$). The solvent was removed in vacuo to afford a dark orange oil. The pro-ligand was purified by silica column chromatography (pentane : ethyl acetate = 4:1) as a pale yellow solid. It was dried for 16 hours under high vacuum (2.11 g, 41 % yield). **R_f** 0.36 (pentane: ethyl acetate = 4:1). **^{1}H NMR** (400 MHz, CDCl3) δ 10.88 (s, 1H, O-$\underline{H}$, e), 9.87 (s, 1H, O=C-$\underline{H}$), 7.13 (dd, J = 15.1, 7.9 Hz, 2H, Ar-$\underline{H}$, b, d), 6.87 (t, J = 7.9 Hz, 1H, Ar-$\underline{H}_{para}$, c), 4.15 (t, J = 4.8 Hz, 2H, O-CH_2-CH_2, f/g), 3.76 – 3.69 (m, 2H, f/g), 3.39 (s, 3H, -C$\underline{H_3}$, h).

'Open ligand' synthesis (H_2L_1)

Ethylenediamine (92.98 µL, 1.28 mmol) was added to a stirred solution of 2-hydroxy-3-(2-methoxyethoxy)benzaldehyde (500 mg, 2.6 mmol) in MeOH (150 mL). The bright yellow solution was left to stir for 16 hours at ambient temperature. The solvent was removed *in vacuo* and H_2L_1 was precipitated from DCM/pentane. The precipitate was dried under high vacuum for 16 hours to afford H_2L_1 as bright yellow, fluffy solid (374 mg, 35% yield). **m/z** $[H_2L_1 + Na]^+$ 439.1827 **^{1}H NMR** (500 MHz, $CDCl_3$) δ 13.56 (s, 2H, O-$\underline{H}$, i), 8.32 (s, 2H, N=C-$\underline{H}$, b), 6.96 (dd, *J* = 7.9, 1.5 Hz, 2H, Ar-$\underline{H}_{m,}$

c/e), 6.87 (dd, J = 7.8, 1.5 Hz, 2H, Ar-$\underline{H}_{m,}$ c/e), 6.76 (t, J = 7.9 Hz, 2H, Ar-$\underline{H}_{p,}$ d), 4.19 (dd, J = 5.8, 4.2 Hz, 4H, CH_3-O-C$\underline{H}_2$, g), 3.94 (s, 4H, C$\underline{H}_2$-O-CH_2, f), 3.79 (dd, J = 5.7, 4.3 Hz, 4H, N-C$\underline{H}_2$, a), 3.45 (s, 6H, C$\underline{H}_3$, h). **^{13}C NMR** (126 MHz, $CDCl_3$) δ 166.66 (N=C, b), 152.08 (Ar-$\underline{C}$-OH, h), 147.39 (Ar-C_{ortho}, c) , 124.07 (Ar-C_{meta}, d/f), 118.84 (Ar-$\underline{C}_{ortho}$-O-CH_2, g) 118.04 (Ar-$\underline{C}_{para}$-H, e), 117.25 (Ar-C_{meta}, d/f), 71.13 (Ar-O-$\underline{C}H_2$, i), 68.84 (CH_2-$\underline{C}H_2$-O, j), 59.56 (N-$\underline{C}H_2$, a), 59.23 ($\underline{C}H_3$, k) **v_{max}/cm^{-1}** 2966 (C(sp^2)-H). Found: C, 63.13; H, 6.43; N, 6.52. Calc. for $C_{26}H_{26}N_2O_6$: C, 63.45; H, 6.78; N, 6.73%.

Co(III)K(I) 'open' complex (1) synthesis

H_2L_1 (300 mg, 0.72 mmol), K(OAc) (71 mg, 0.72 mmol) and $Co(OAc)_2$ (128 mg, 0.72 mmol) were stirred in dry acetonitrile (100 mL) for 16 hours under an inert N_2 atmosphere. The solution was then opened to air and acetic acid (82.4 µL, 1.44 mmol) was added. The solution was stirred for 72 hours in air. The solvent was removed in *vacuo* and six azeotropic washes (toluene (3x 10 mL), pentane (3x 10 mL)) were performed to afford a light brown powder. The complex was reprecipitated from DCM/pentane (75 mL). The resulting brown, fluffy material was dried under high vacuum for 24 hours to afford (**1**) in good yields (225 mg, 75%). **^{1}H NMR** (500 MHz, $CDCl_3$) δ 7.64 (s, 2H, N=C-$\underline{H}$, b), 6.80 (d, J = 7.8 Hz, 2H, Ar-$\underline{H}_{m,}$ c/e), 6.71 (d, J = 7.5 Hz, 2H, Ar-$\underline{H}_{m,}$ c/e), 6.34 (t, J = 7.7 Hz, 1H, Ar-$\underline{H}_{p,}$ d), 4.26 (s, 4H, N-C$\underline{H}_2$, a), 4.14 – 4.09 (m, 4H, C$\underline{H}_2$-O-CH_2, f), 3.80 – 3.75 (m, 4H, CH_3-O-C$\underline{H}_2$, g), 3.41 (s, 6H, C$\underline{H}_3$, h), 1.38 (s, 6H, O-Ac, i). **^{13}C NMR** (126 MHz, $CDCl_3$) δ 164.60 (C=O (OAc), l), 157.82 (N=C, b), 152.07 (Ar-$\underline{C}$-OH, g), 126.83 (Ar-$\underline{C}_{ortho}$-O-CH_2, h), 119.28 (Ar-C_{meta}, d/f), 115.18 (Ar-C_{ortho}, c), 112.28 (Ar-C_{meta}, d/f),, 70.77 (CH_2-$\underline{C}H_2$-O, j), 67.17 (Ar-O-$\underline{C}H_2$, i), 58.93 (N-$\underline{C}H_2$, a), 58.23 ($\underline{C}H_3$, k), , 24.68 ($H_3\underline{C}$-C=O (OAc), l).) **v_{max}/cm^{-1}** 2914 (C(sp^2)-H). Found: C, 49.73; H, 4.91; N, 4.35. Calc. for $C_{26}H_{32}N_2O_{10}CoK$: C, 49.52%; H, 5.12%; N, 4.44%.

Co(III)Na (I) 'open' complex (2) synthesis

H_2L_1 (200 mg, 0.48 mmol), Na(OAc) (39 mg, 0.48 mmol) and $Co(OAc)_2$ (85 mg, 0.48 mmol) were stirred in dry acetonitrile (75 mL) for 16 hours under an inert N_2 atmosphere. The solution was then opened to air and acetic acid (27.45 µL, 0.96 mmol) was added. The solution was stirred for 72 hours in air. The solvent was removed in *vacuo* and six azeotropic washes (toluene (3x 10 mL), pentane (3x 10 mL)) were performed to afford a light brown powder. The complex was reprecipitated from DCM/pentane (75 mL). The resulting light brown powder was dried under high vacuum, for 24 hours to afford (**2**) (147 mg, 50% yield). ^{1}H NMR (500 MHz, $CDCl_3$) δ 7.75 (s, 2H N=C-$\underline{H}$, b), 6.86 (ddd, J = 24.1, 7.8, 1.6 Hz, 4H, Ar-$\underline{H}_{m,}$ c + e), 6.37 (t, J = 7.7 Hz, 2H, Ar-$\underline{H}_{p,}$ d), 4.24 (s, 4H, N-C$\underline{H}_2$, a), 4.20 – 4.12 (m, 4H, C$\underline{H}_2$-O-CH_2, f), 3.76 – 3.69 (m, 4H, CH_3-O-C$\underline{H}_2$, g), 3.52 (s, 6H, C$\underline{H}_3$, h), 1.33 (s, 6H, O-Ac, i). ^{13}C NMR (126 MHz, $CDCl_3$) δ 179.16 (C=O (OAc), l), 164.73 (N=C, b), 158.78 (Ar-$\underline{C}$-OH, g), 152.01(Ar-$\underline{C}_{ortho}$-O-CH_2, h), 127.89 (Ar-C_{meta}, d/f), 120.14(Ar-C_{ortho}, c) , 119.46 (Ar-C_{meta}, d/f), 112.81 (Ar-C_{para}, e), 71.01(CH_2-$\underline{C}H_2$-O, j/ Ar-O-$\underline{C}H_2$, i), 70.90 (CH_2-$\underline{C}H_2$-O, j/ Ar-O-$\underline{C}H_2$, i), 59.23 (N-$\underline{C}H_2$, a / $\underline{C}H_3$, k), 59.14 (N-$\underline{C}H_2$, a / $\underline{C}H_3$, k), 24.86 ($H_3\underline{C}$-C=O (OAc), l). **v_{max}/cm^{-1}** 2906 (C(sp^2)-H). Found: C, 50.87%; H, 5.06%; N, 4.44%. Calc. for $C_{26}H_{32}N_2O_{10}CoNa$: C, 50.82%; H, 5.25%; N, 4.56%

Co(III)Rb(I) 'open' complex (3) synthesis

H_2L_1 (200 mg, 0.48 mmol), Rb(OAc) (70 mg, 0.48 mmol) and $Co(OAc)_2$ (85 mg, 0.48 mmol) were stirred in dry acetonitrile (75 mL), for 16 hours, under an inert N_2 atmosphere. The solution was then opened to air and acetic acid (27.45 µL, 0.96 mmol) was added. The solution was stirred for 72 hours in air. The solvent was removed in *vacuo* and six azeotropic washes (toluene (3x 10 mL), pentane (3x 10 mL)) were performed to afford a light brown powder. The complex was reprecipitated from

DCM/pentane (75 mL). The resulting dark brown soldi was dried under high vacuum for 24 hours to afford (**2**) (44 mg, 13% yield). ^{1}H NMR (500 MHz, $CDCl_3$) δ 7.67 (s, 2H, N=C-H, b), 6.86 (dd, *J* = 7.9, 1.6 Hz, 2H, Ar-$H_{m,}$ c/e), 6.76 (dd, *J* = 7.7, 1.6 Hz, 2H, Ar-$H_{m,}$ c/e), 6.40 (t, *J* = 7.7 Hz, 2H, Ar-$H_{p,}$ d), 4.36 (s, 4H, N-CH_2, a), 4.19 – 4.13 (m, 4H, CH_2-O-CH_2, f), 3.88 – 3.82 (m, 4H, , CH_3-O-CH_2, g), 3.46 (s, 6H, CH_3, h), 1.47 (s, 6H, 6H, O-Ac, i). **^{13}C NMR** (126 MHz, $CDCl_3$) δ 179.26 (C=O (OAc), l), 164.90(N=C, b), 157.54 (Ar-C-OH, g), 152.11 83 (Ar-C_{ortho}-O-CH_2, h), 126.86(Ar-C_{meta}, d/f), 119.36(Ar-C_{ortho}, c), 114.66 (Ar-C_{meta}, d/f), 112.27(Ar-C_{para}, e), 70.87 (CH_2-CH_2-O, j), 66.84 (Ar-O-CH_2, i), 58.94 (N-CH_2, a), 58.12(CH_3, k), 24.67 (H_3C-C=O (OAc), l). **v_{max}/cm^{-1}** 2916 (C(sp^2)-H). Found: C, 47.44%; H, 4.33%; N, 3.79%. Calc. for $C_{26}H_{32}N_2O_{10}CoRb$: C, 46.13%; H, 4.76%; N, 4.14%.

Additional Information

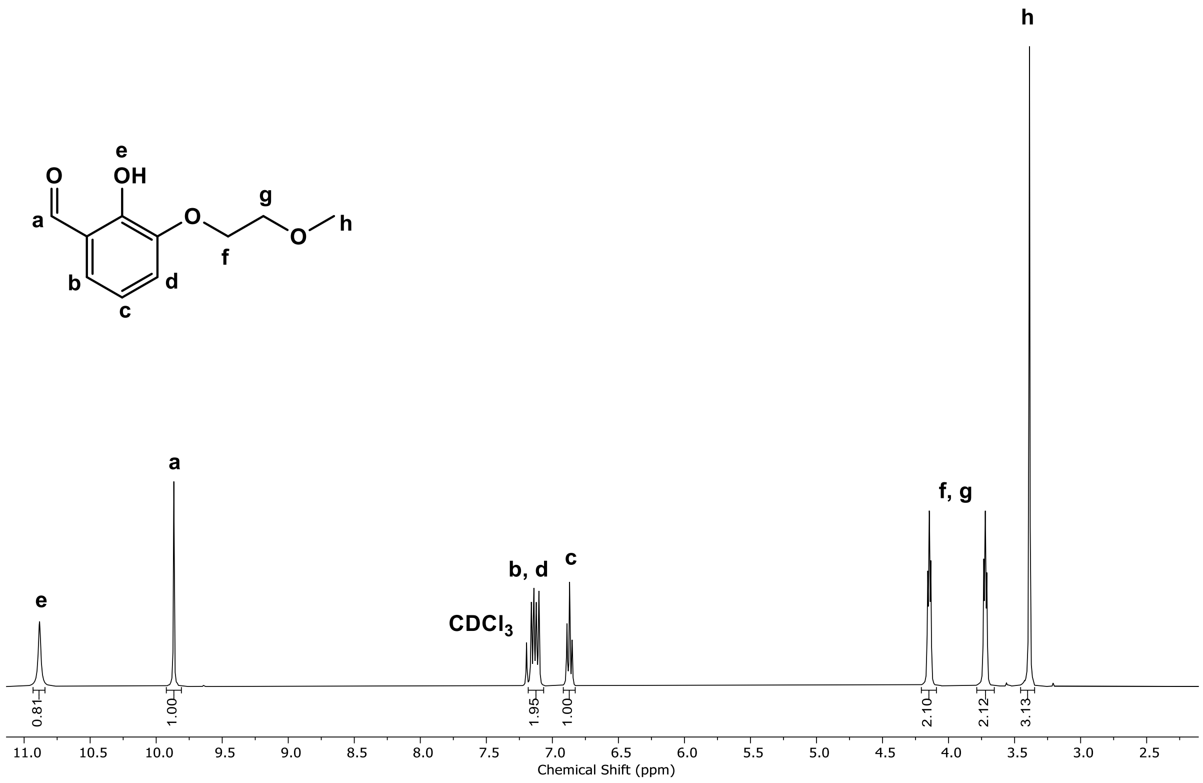

Fig. S1 ^{1}H NMR spectrum of 2-hydroxy-3-(2-methoxyethoxy)benzaldehyde pro-ligand in $CDCl_3$.

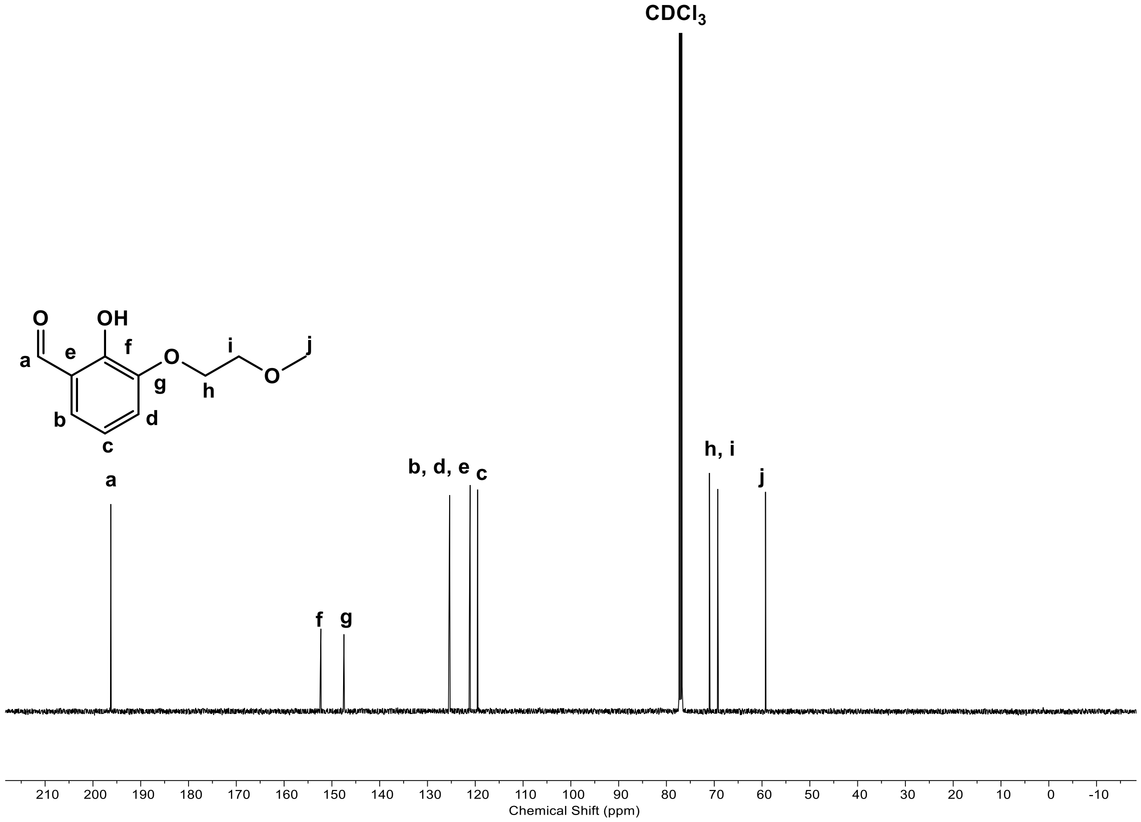

Fig. S2 ^{13}**C NMR spectrum of the 2-hydroxy-3-(2-methoxyethoxy)benzaldehyde pro-ligand in** $CDCl_3$**.**

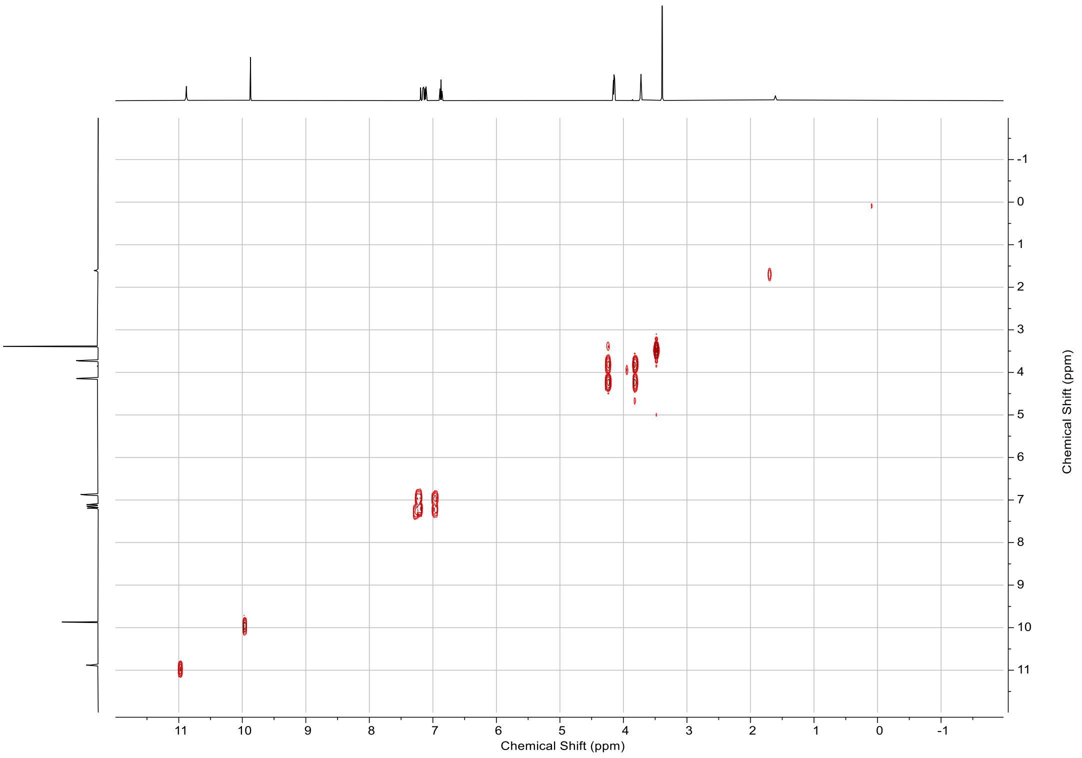

Fig. S3 COSY NMR spectrum of the 2-hydroxy-3-(2-methoxyethoxy)benzaldehyde pro-ligand in $CDCl_3$**.**

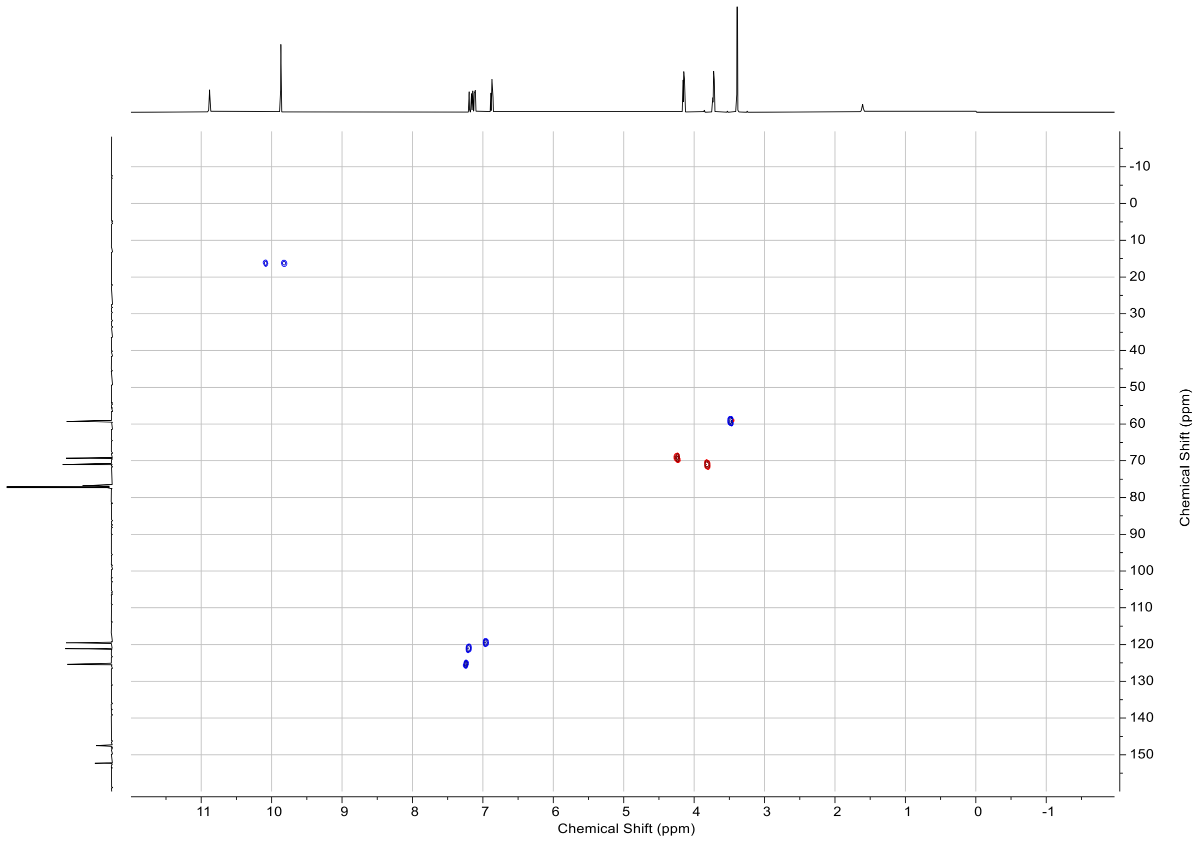

Fig. S4 HSQC NMR spectrum of 2-hydroxy-3-(2-methoxyethoxy)benzaldehyde pro-ligand in $CDCl_3$.

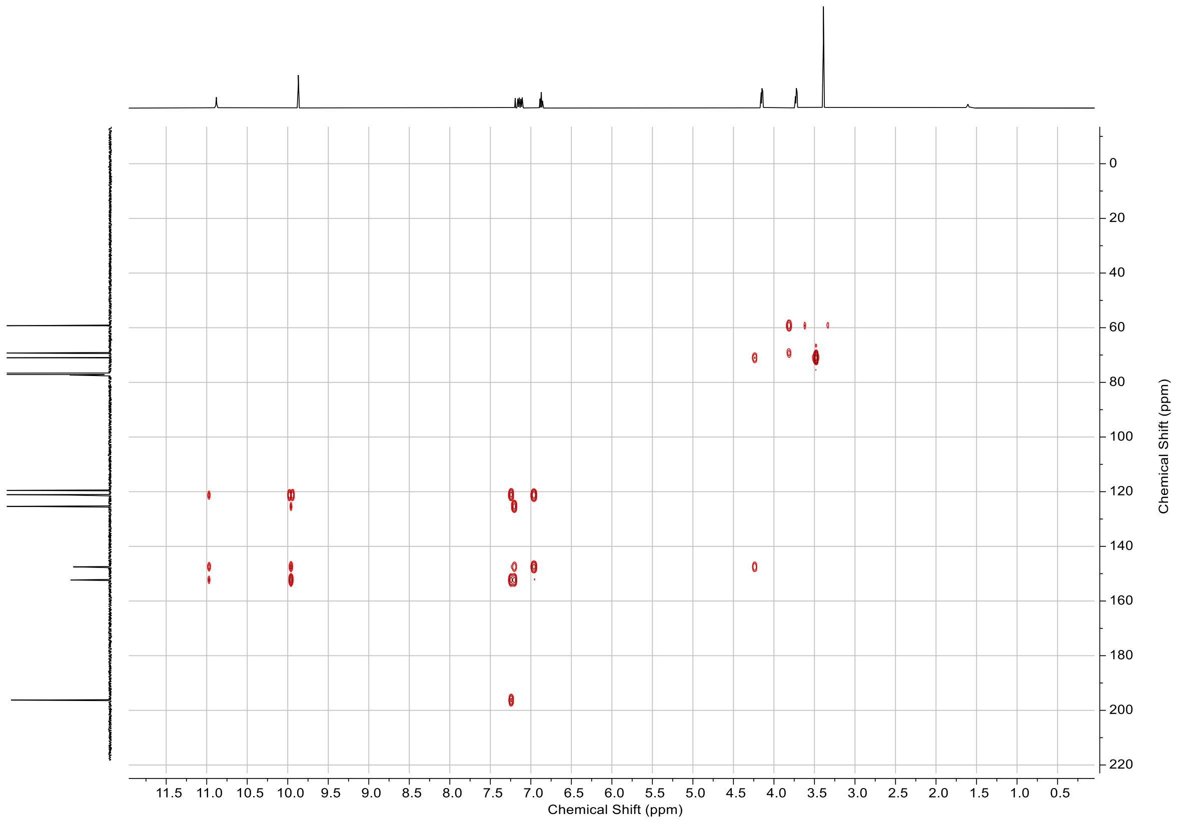

Fig. S5 HMBC NMR spectrum of 2-hydroxy-3-(2-methoxyethoxy)benzaldehyde pro-ligand in $CDCl_3$.

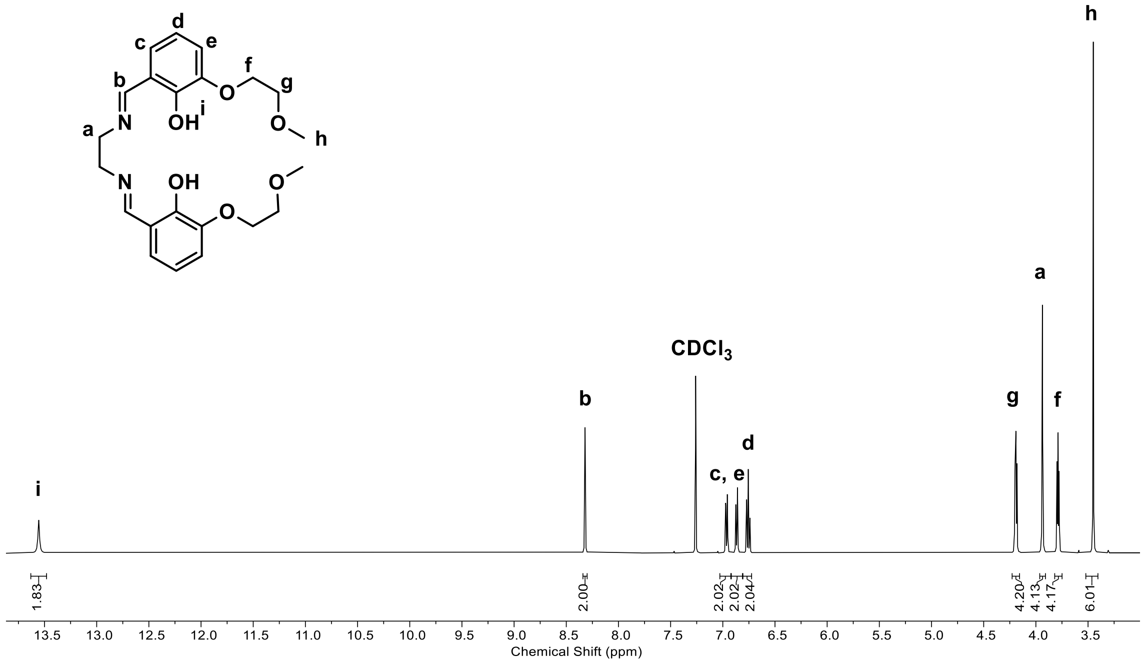

Fig. S6 ^{1}H **NMR spectrum of** H_1L_2 **in** $CDCl_3$**.**

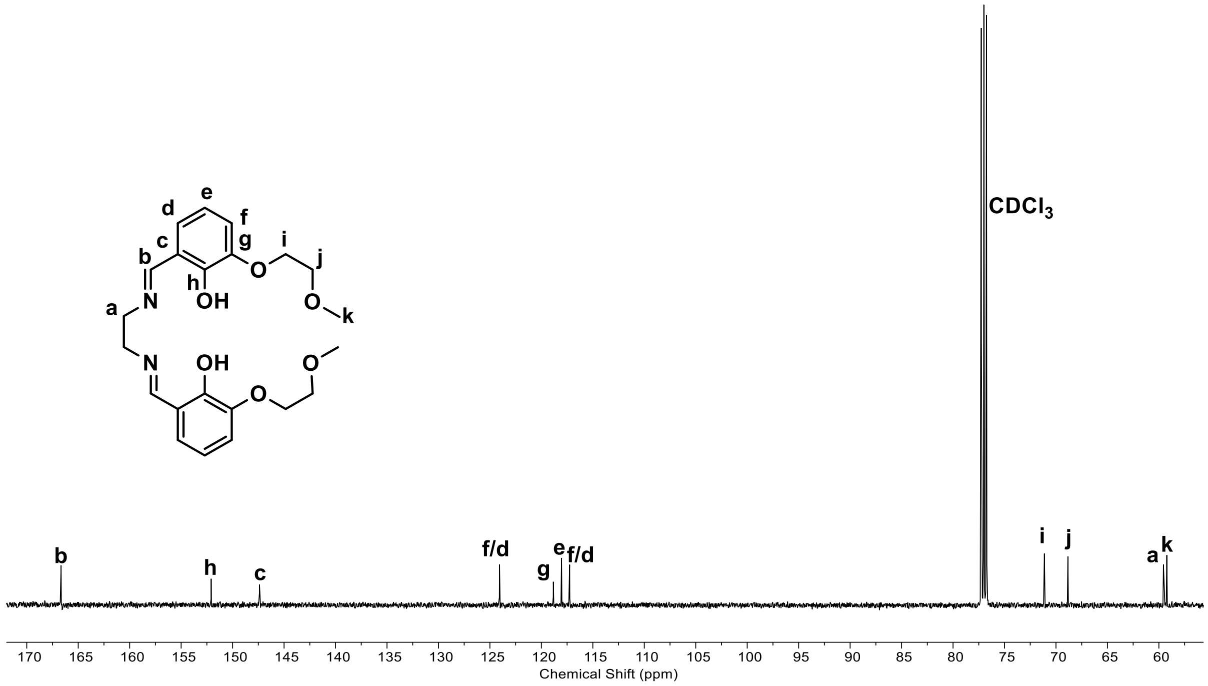

Fig. S7 ^{13}C **NMR spectrum of** H_1L_2 **in** $CDCl_3$**.**

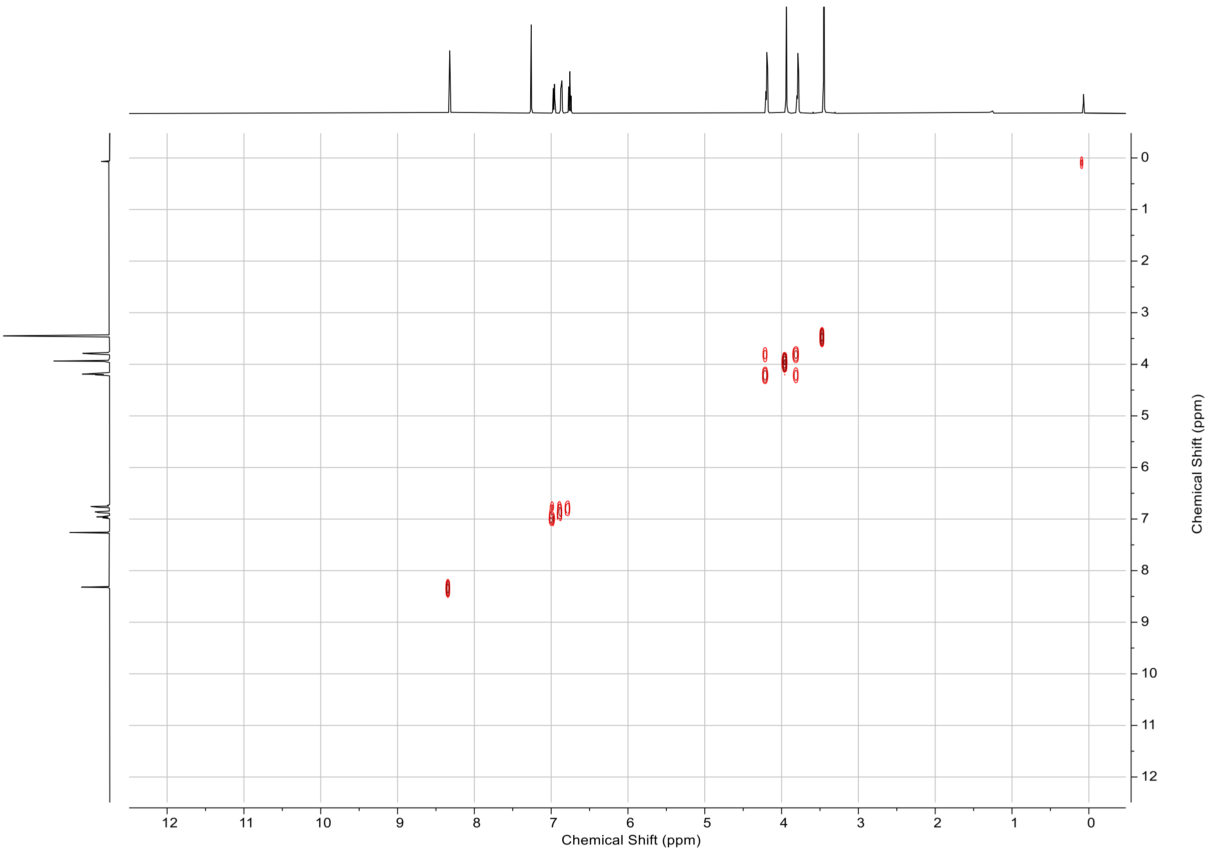

Fig. S8 COSY NMR spectrum of H_1L_2 in $CDCl_3$.

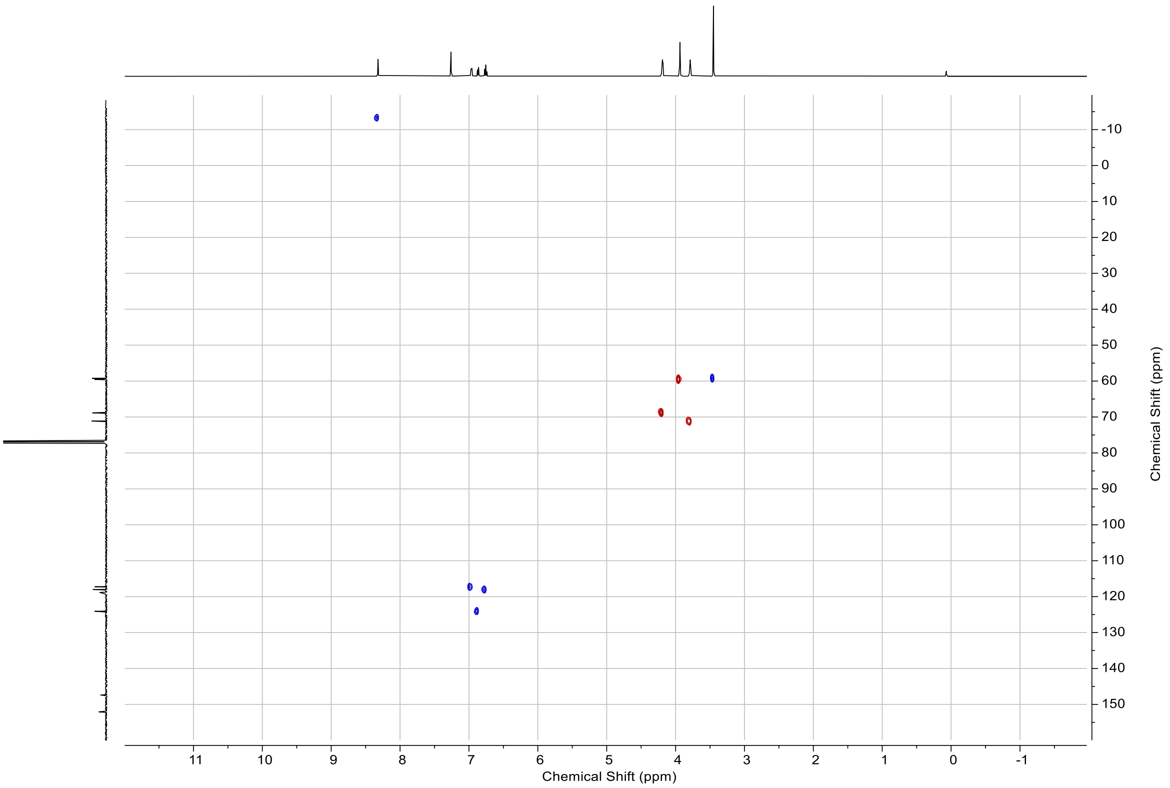

Fig. S9 HSQC spectrum of H_1L_2 in $CDCl_3$.

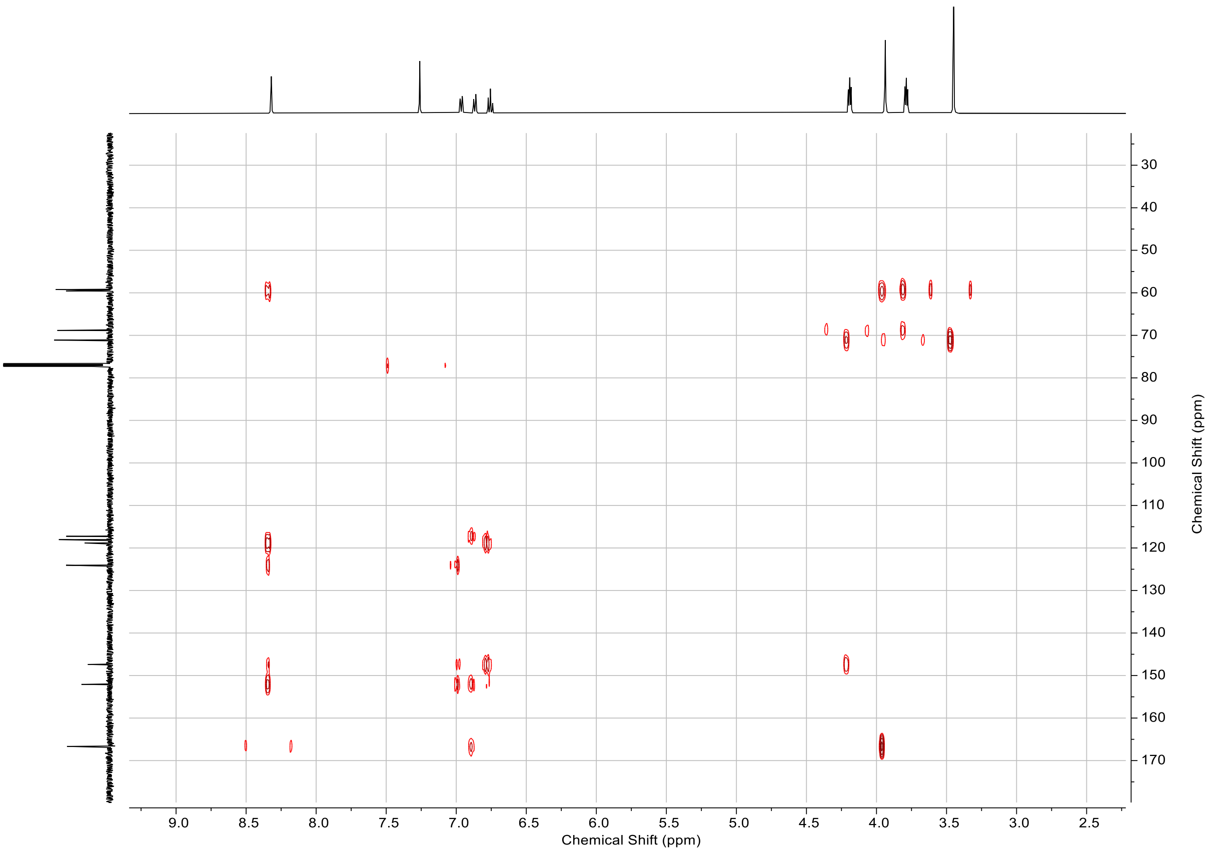

Fig. S10 HMBC spectrum of H_1L_2 in $CDCl_3$.

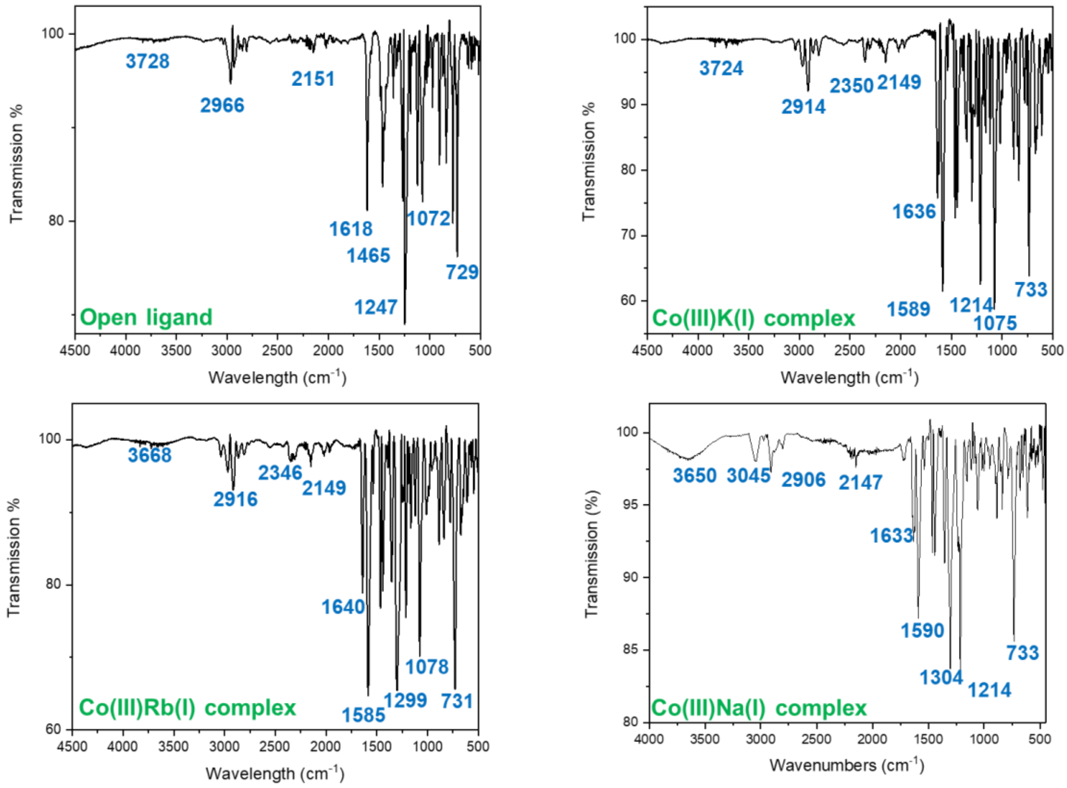

Fig. S11 FT-IR spectra of H_2L_1, Co(III)K(I) (1), Co(III)Na(I) (2) and Co(III)Rb(I) (3).

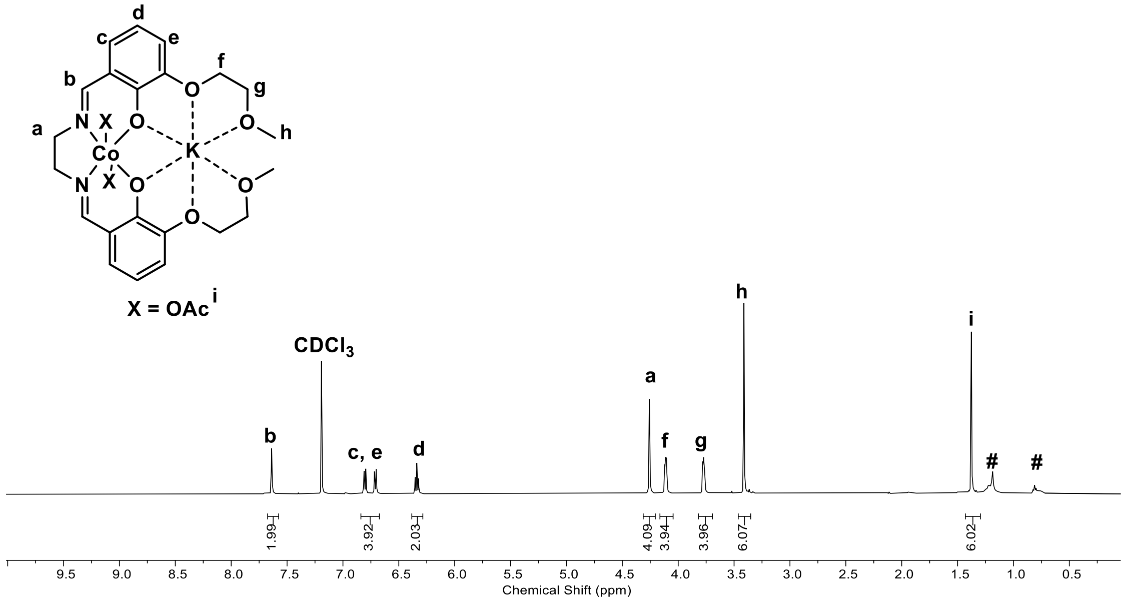

Fig. S12 ¹H NMR spectrum of (1) in CDCl₃.

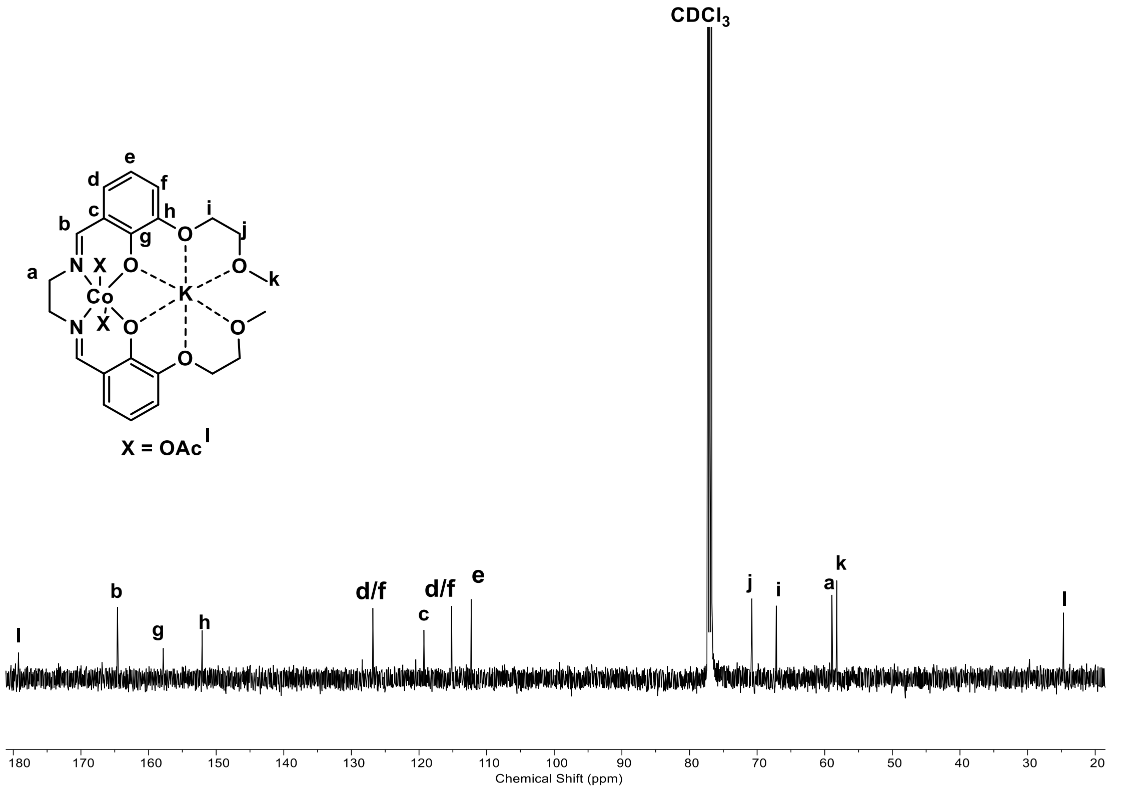

Fig. S13 ¹³C NMR spectrum of (1) in CDCl₃.

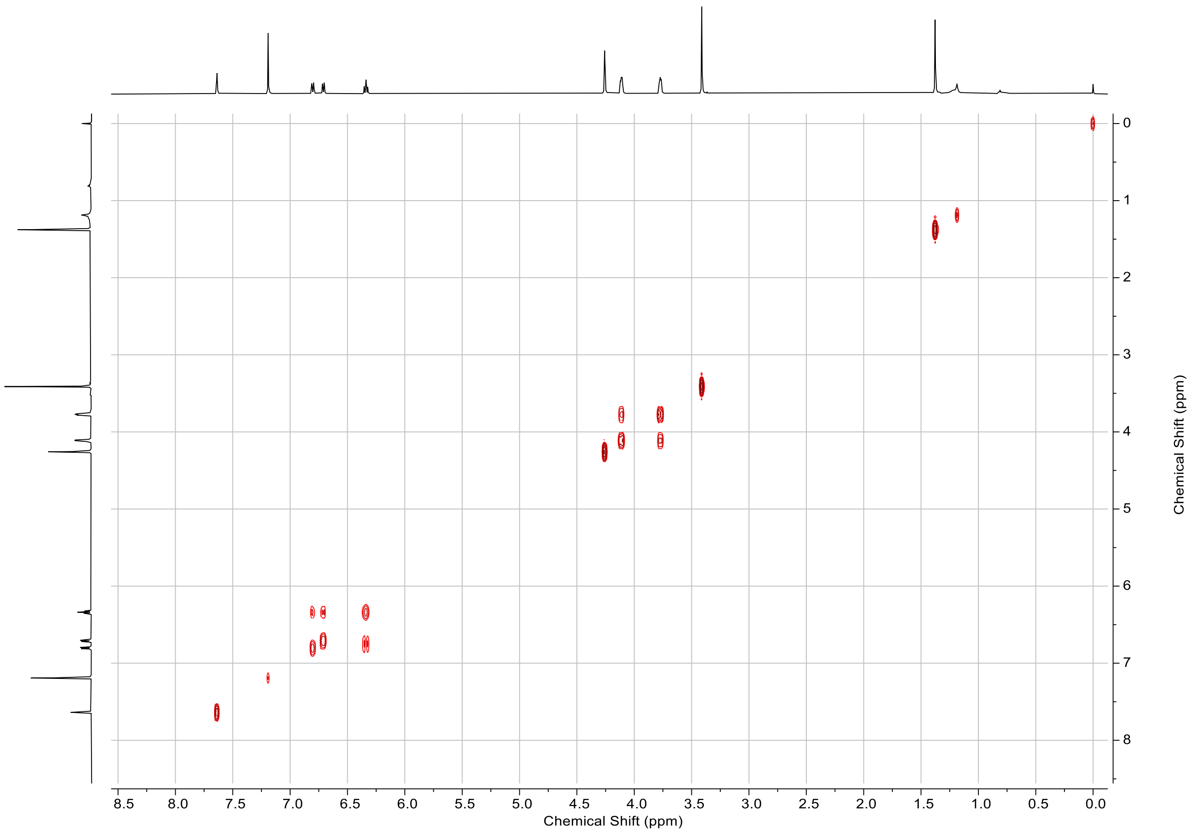

Fig S14 COSY NMR spectrum of (1) in $CDCl_3$.

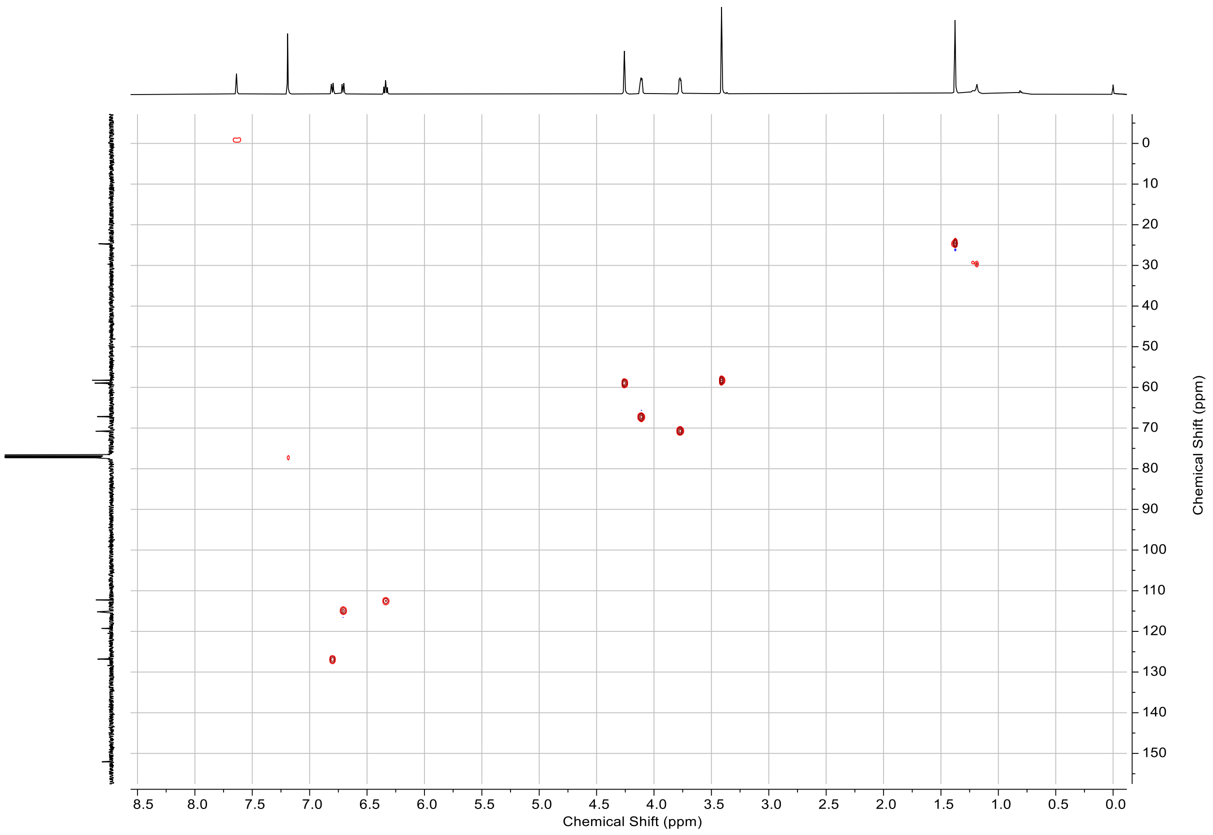

Fig. S15 HSQC NMR spectrum of (1) in $CDCl_3$.

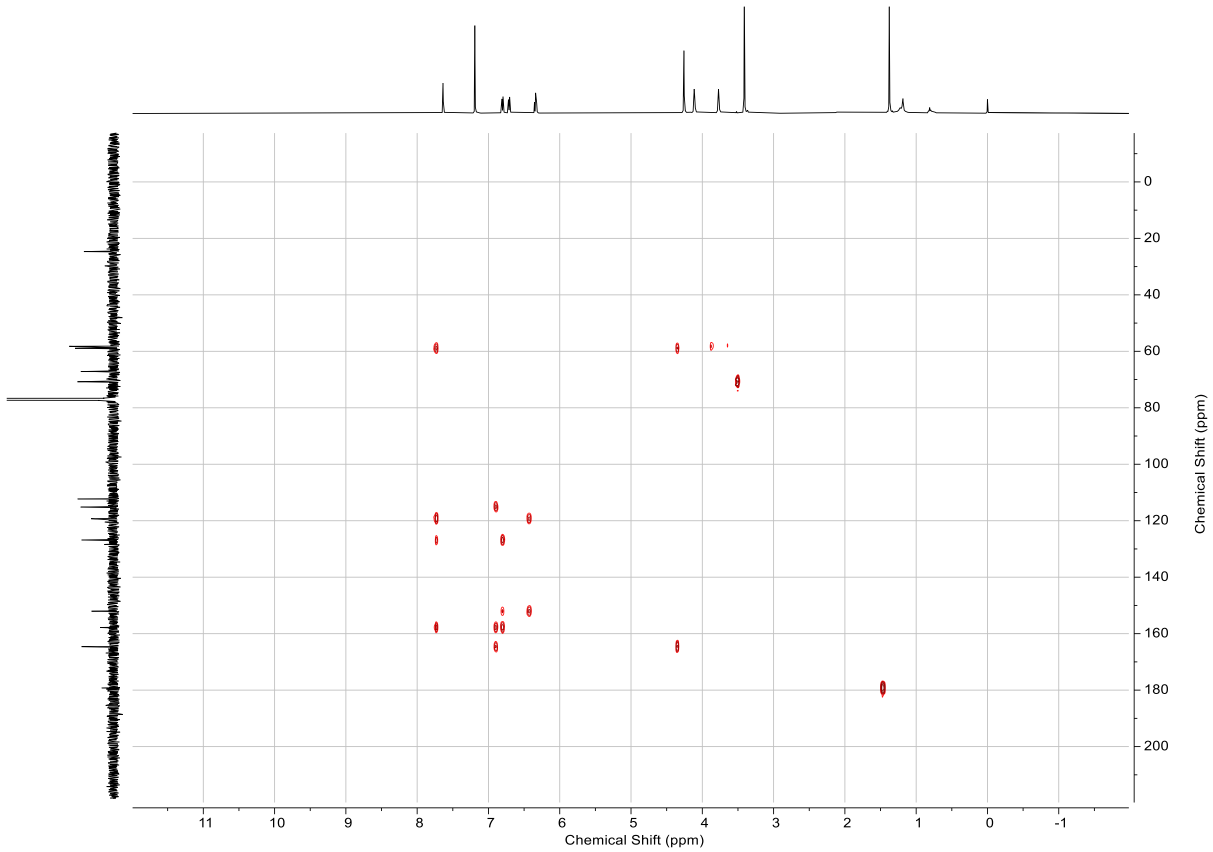

Fig. S16 HMBC NMR spectrum of (1) in CDCl₃.

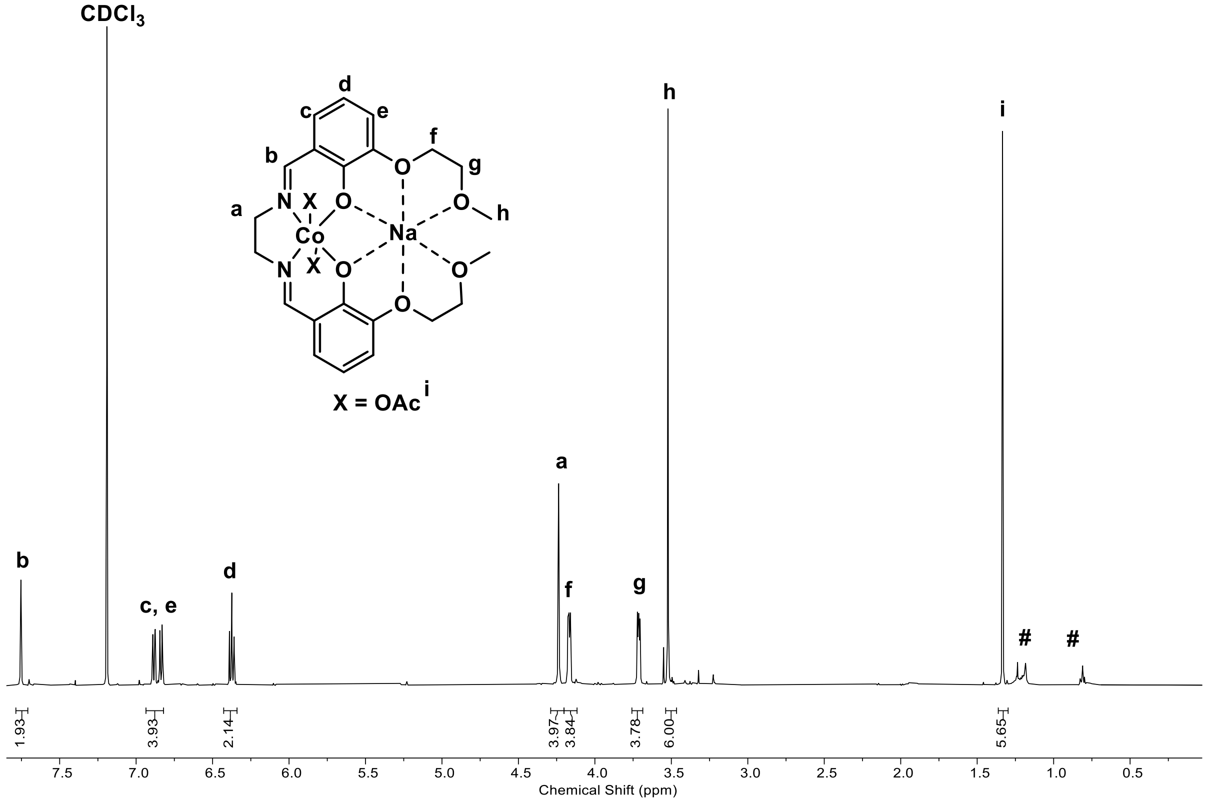

Fig. S17 ^{1}H NMR spectrum of (2) in $CDCl_3$.

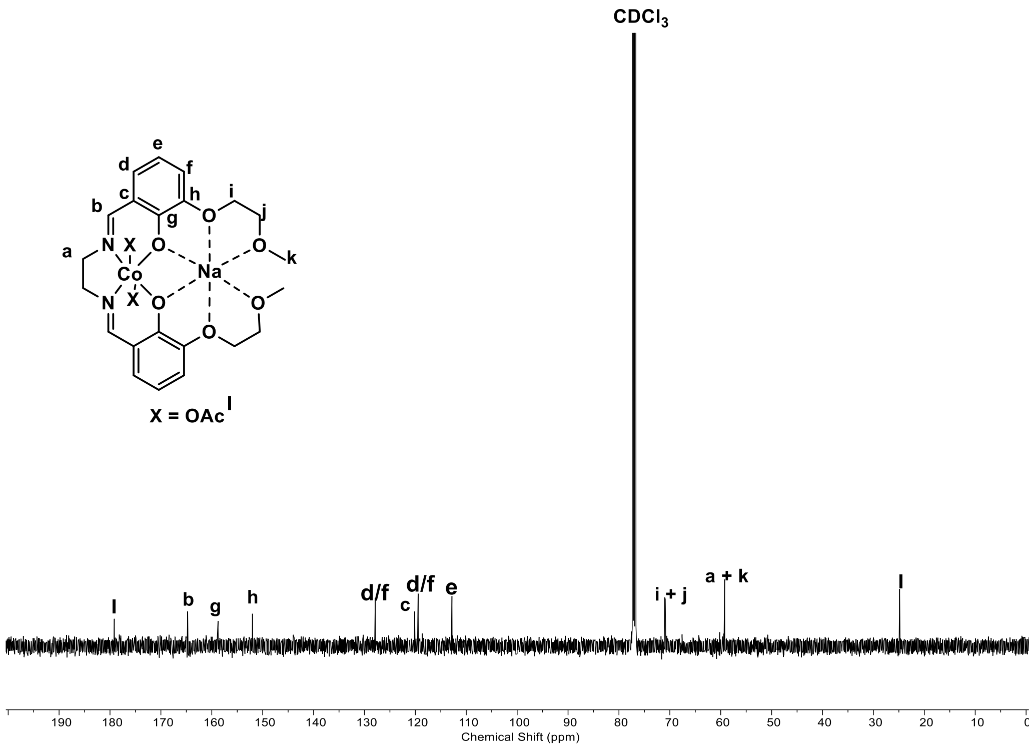

Fig. S18 ^{13}C NMR spectrum of (2) in $CDCl_3$.

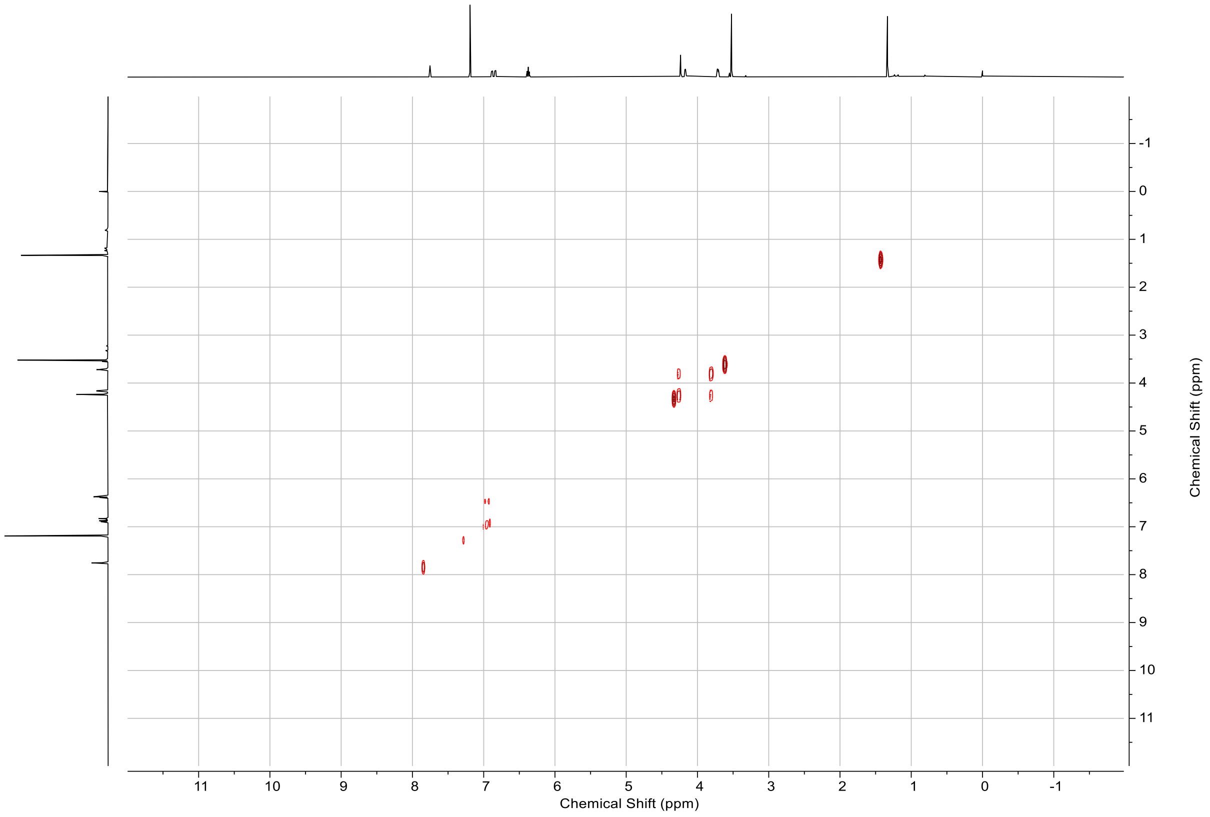

Fig S19 COSY NMR spectrum of (2) in $CDCl_3$.

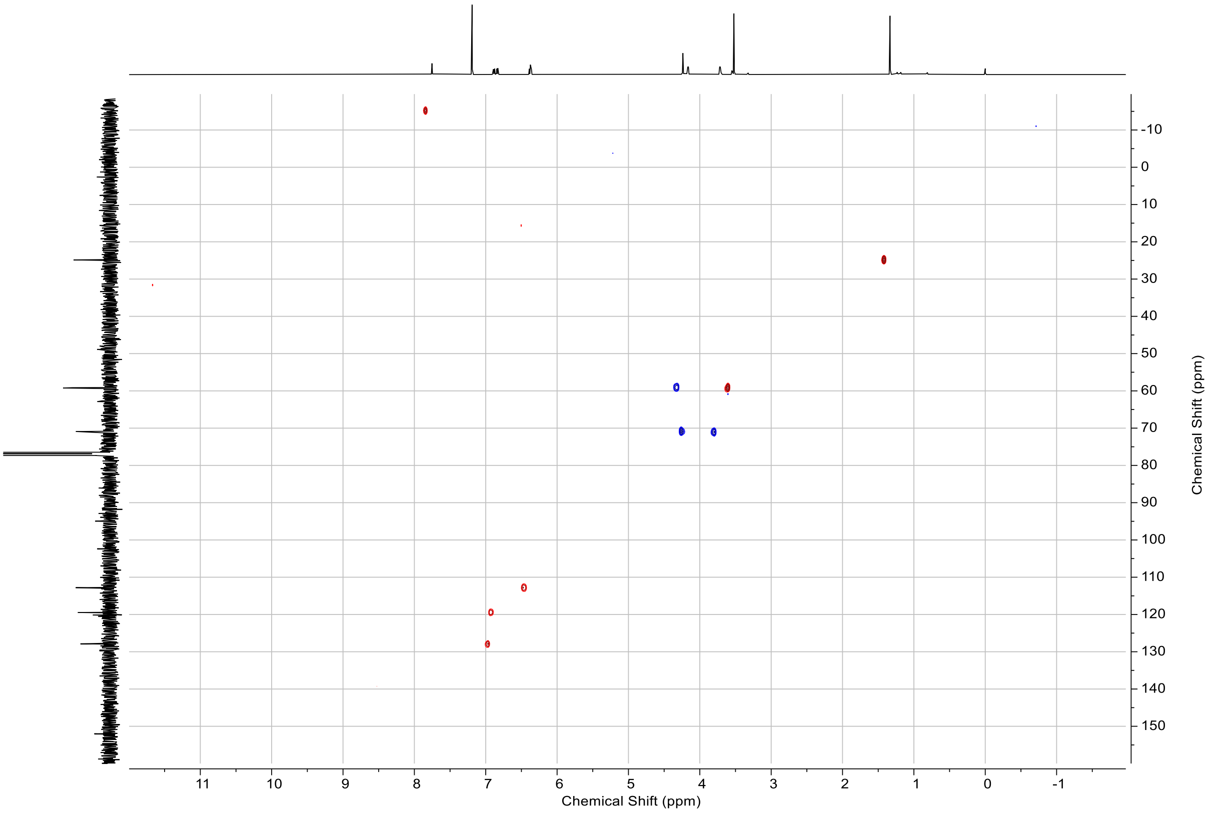

Fig. S20 HSQC NMR spectrum of (2) in $CDCl_3$.

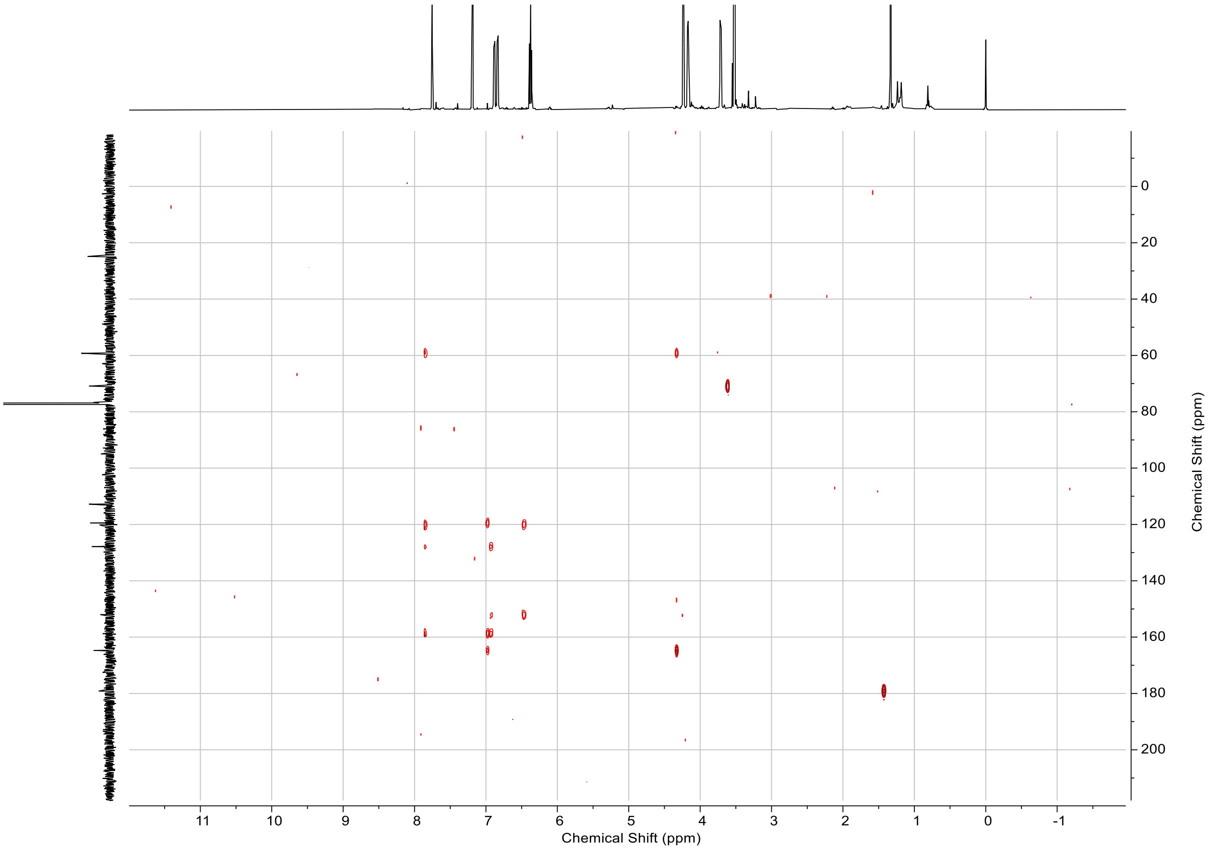

Fig. S21 HMBC spectrum of (2) in $CDCl_3$.

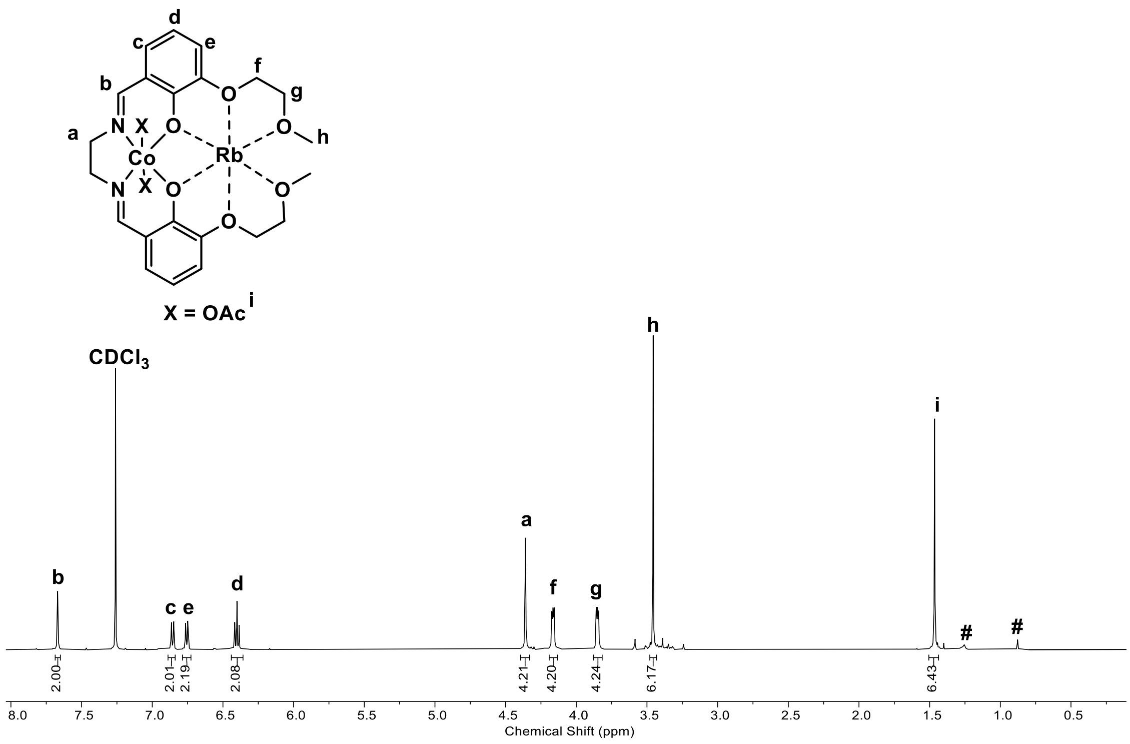

Fig. S22 ^{1}H NMR spectrum of (3) in $CDCl_3$. Residual H-grease (#).

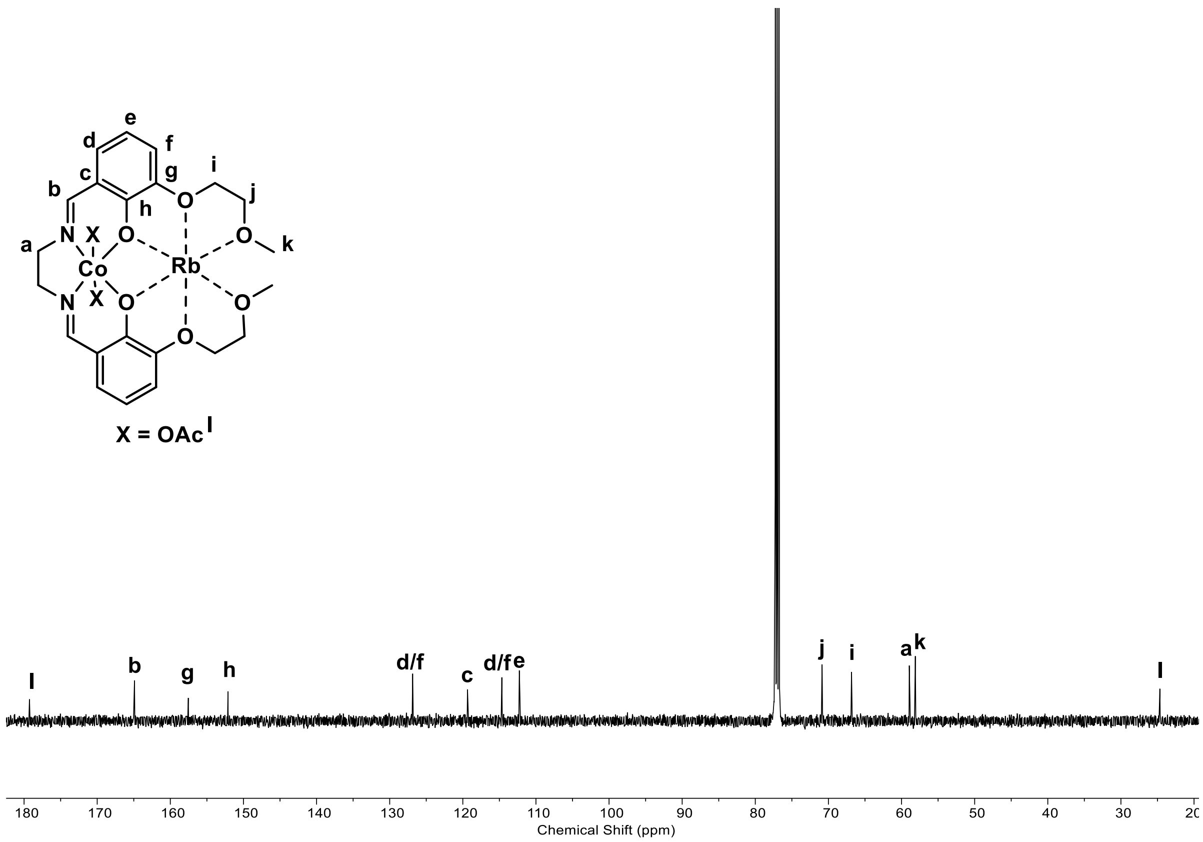

Fig. S23 ^{13}C NMR spectrum of (3) in $CDCl_3$.

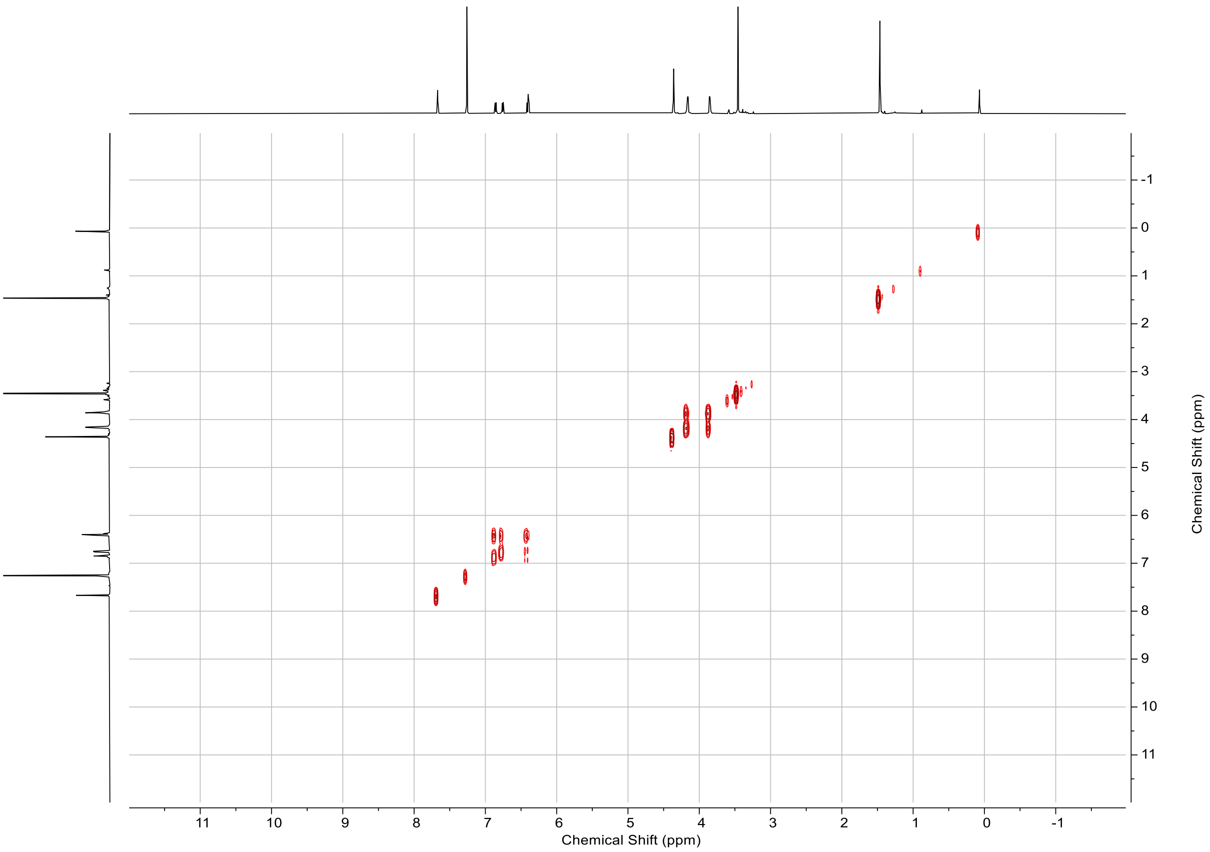

Fig. S24 COSY NMR spectrum of (3) in $CDCl_3$.

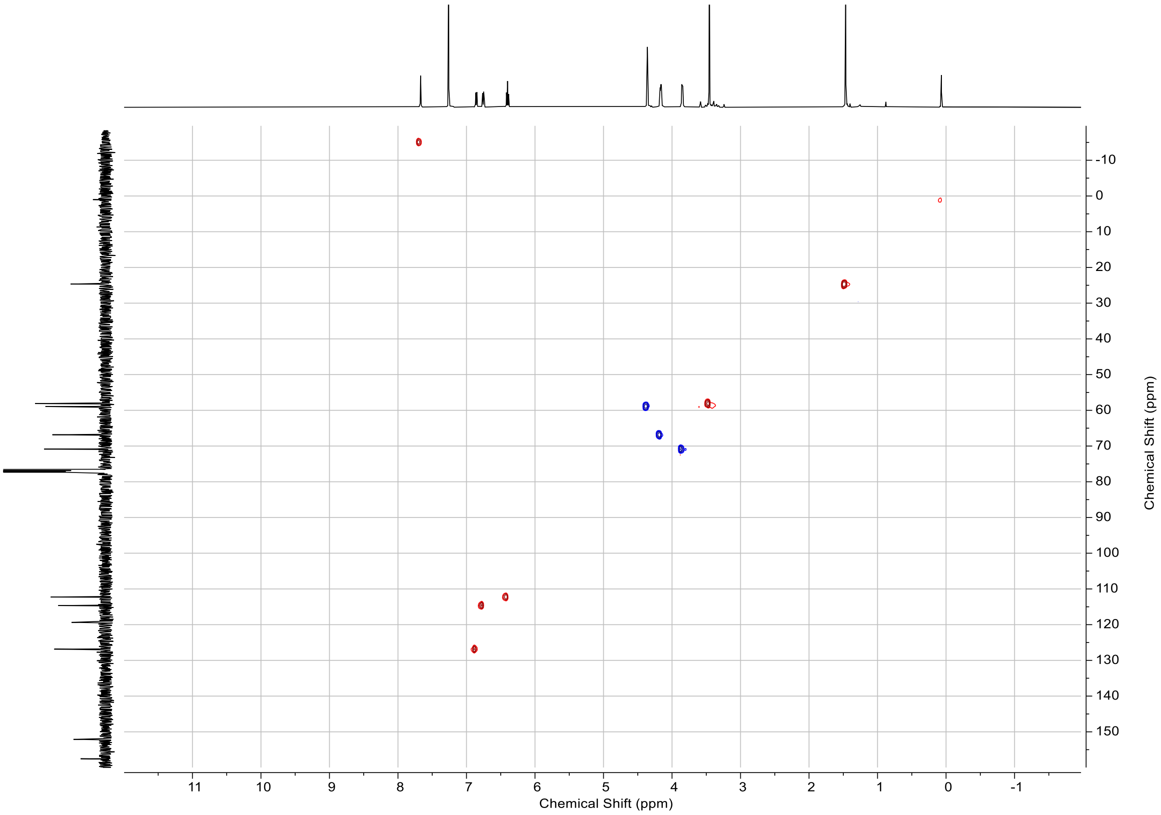

Fig. S25 HSQC NMR spectrum of (3) in $CDCl_3$.

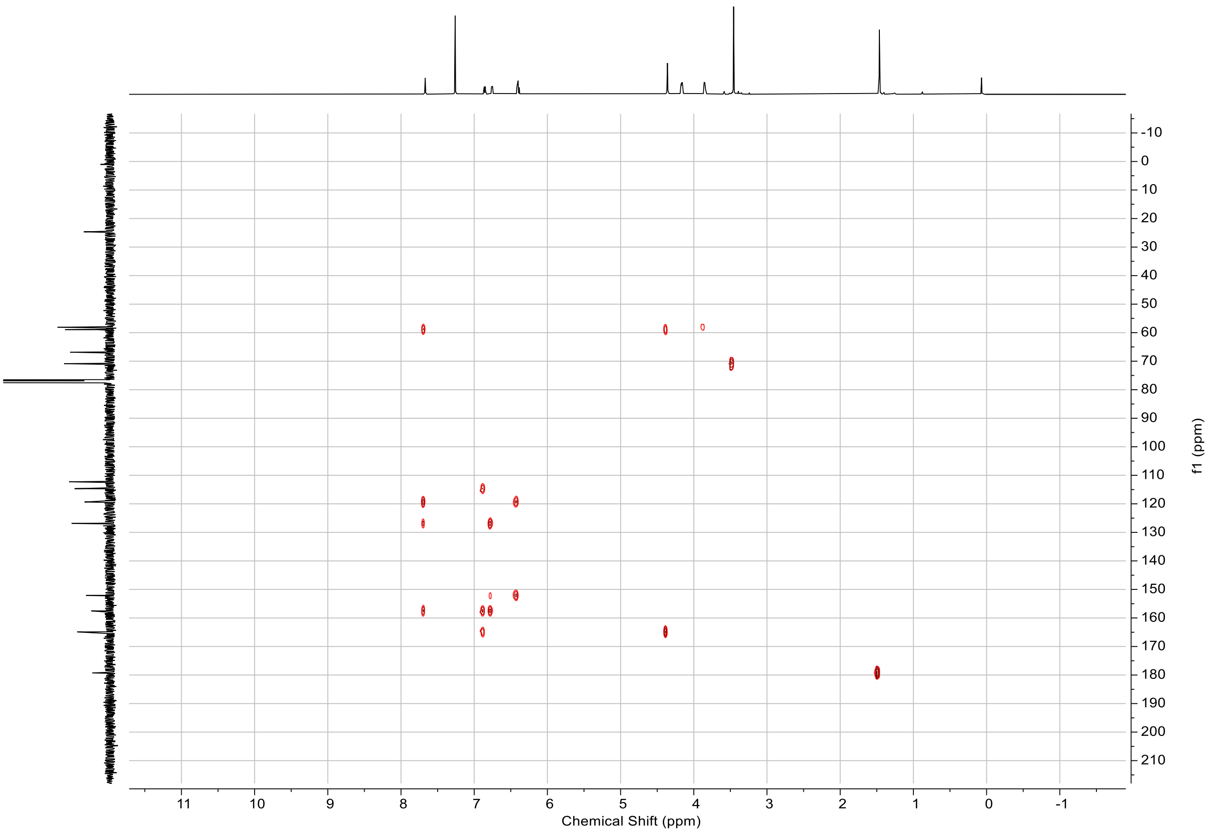

Fig. S26 HMBC NMR spectrum of (3) in $CDCl_3$.]

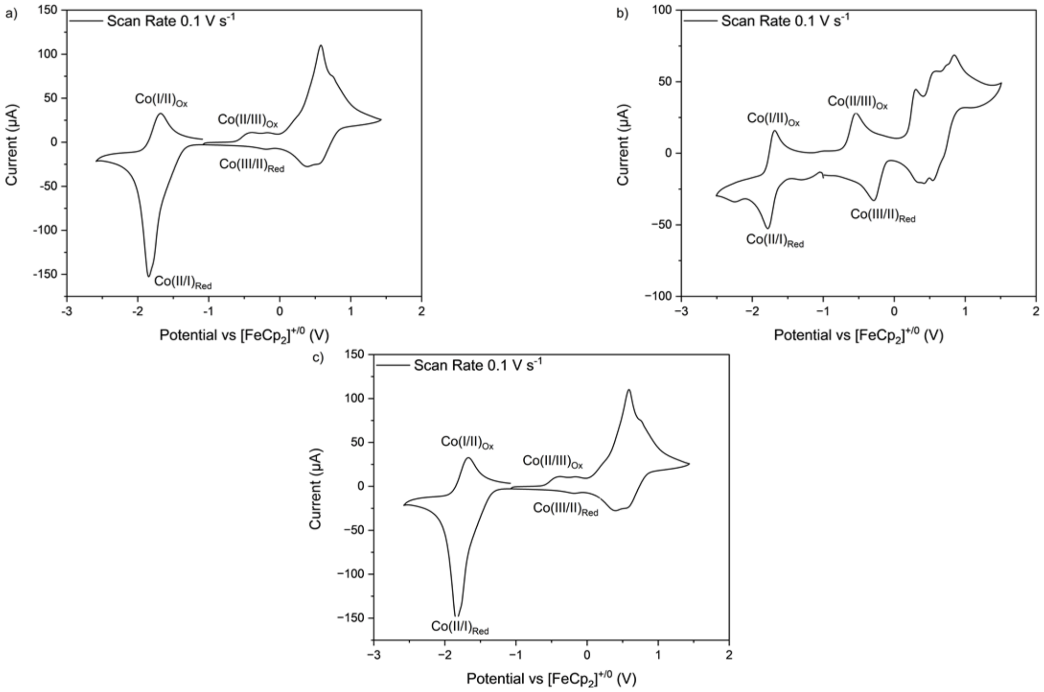

Fig. S27 CV graphs of complexes a) Co(III) Na(I), b) Co(III)K(I), c) Co(III)Rb(I). All complexes show similar Co(II/I) reduction potentials of a) E_{red}Co(II/I) = - 1.85 V b) E_{red}Co(II/I) = - 1.78 V c) E_{red}Co(II/I) = - 1.84 V. All unassigned peaks are associated with ligand reductions and oxidations.

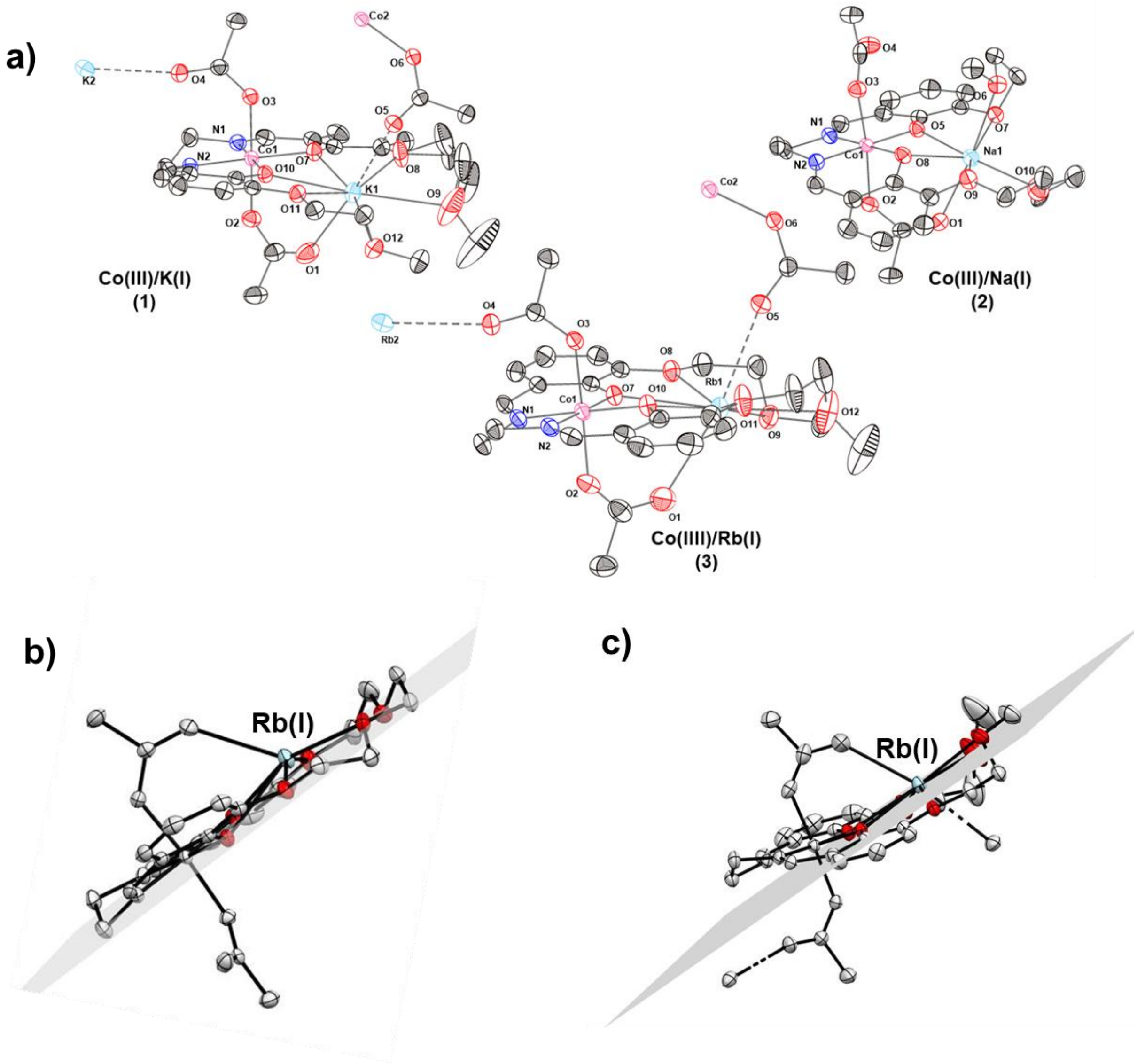

Fig. S28 Overview of single crystal XRD structures. (a) ORTEP representation of the molecular structures of catalysts 1 - 3 with hydrogen atoms and residual solvents omitted for clarity. Co(III)K(I) (1) and Co(III)Rb(I) (3) are polymeric in the solid state, Co(III)Na(I) (**2**) is monomeric. Details for all structures obtained by single XRD are given in Table S15-17. **(b) ORTEP representation of the molecular structures of the previously reported 'closed' Co(III)Rb(I) catalyst and (c)'open' Co(III)Rb(I) (3);** highlighted in red are the ether oxygens that were used to calculated the plane shown in grey. The distance between the ether plane (grey) and the Rb(I) ion (light blue) was determined as 0.523 Å and 0.199 Å, for the 'closed' Co(III)Rb(I) catalyst and the 'open' Co(III)Rb(I) catalyst (**3**), respectively.

Tab. S1 Experimental values used to calculated averages in Table 2[a]

Entry	T / °C	P / bar	Time / h	CO_2 / %[b]	PPC / %[c]	TON[d]	TOF / h^{-1} [e]	k_{obs} 10^{-5}/ s^{-1} [f]	M_n [Đ] / g mol^{-1} [g]
1	50	20	4	>99	>99	1413	339	3.43	8000 [1.03]
2	50	20	4	>99	99	1312	316	3.45	7300 [1.05]
3	60	20	2	>99	98	1650	868	9.42	6900 [1.06]
4	60	20	2	99	>99	878	799	7.25	3800 [1.06]
5	65	20	1.5	>99	94	1351	821	8.79	6600 [1.11]
6	65	20	1.5	>99	93	1538	839	10.0	4600 [1.03]
7	70	20	2	>99	97	2286	994	15.9	11000 [1.03]
8	70	20	2	>99	96	1570	1095	11.9	7500 [1.03]
9	80	20	1.1	>99	90	1730	1549	20.6	7100 [1.04]
10	80	20	0.7	>99	93	1176	1907	22.3	4000 [1.04]
11	50	2	4	>99	86	639	167	0.86	2600 [1.09]
12	50	2	4	99	88	595	198	1.20	2700 [1.06]
13	50	5	4	>99	96	823	204	1.67	-
14	50	5	4	99	95	740	341	2.59	2700 [1.06]
15	50	10	3	>99	98	1023	335	3.00	5900 [1.07]
16	50	10	3	>99	97	1551	370	4.29	6900 [1.04]

[a] Reaction conditions: catalyst (0.025 mol%, 3.6 mM), PO (6 mL, 14.3 M), *trans*-1,2-cyclohexanediol (0.5 mol%, 71 mM) under static CO_2 pressure. [b]CO_2 uptake was calculated by dividing the sum of integrals for polycarbonate and cyclic carbonate against the sum of integrals for polycarbonate, cyclic carbonate and polyether. [c]Polymer selectivity was determined by dividing the sum of integrals for polycarbonate and polyether against the sum of integrals for polycarbonate, cyclic carbonate and polyether. [d]TON was determined by dividing the moles of epoxide consumed (PO: determined by comparison of the sum of integrals by ^{1}H NMR spectroscopy of PPC (4.92 ppm, 1H), PC (4.77 ppm, 1H) and PPO (3.46-3.64 ppm, 3H) against mesitylene (0.25 mol%, 36 mM) as an internal standard;[e]Turnover frequency (TOF) was calculated by dividing the Turnover number (TON) against time.. [f]k_{obs} determined as the gradient of the plot of $\ln[PO]_t/[PO]_0$ *vs.* time . [g]Determined by GPC in THF using narrow dispersity polystyrene standards.

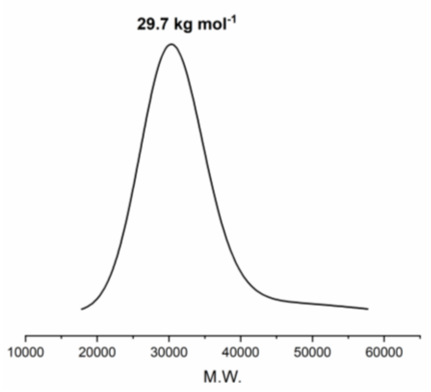

Fig. S29 Monomodal GPC trace for high weight PPC, synthesised using 1 (Tab. 2, entry 10).

Tab. S2 Depolymerisation of PPC using 1[a]

Entry	T (K)	k_p (dm^3 mol^{-1} s^{-1})	Average k_p (dm^3 mol^{-1} s^{-1})[b]	ln(k_p/T)
1	323.15	4.10	4.18 ± 0.06	-4.35 ± 1.44 x 10^{-2}
2	323.15	4.27		
3	328.15	4.53	5.68 ± 0.81	-4.06 ± 1.43 x 10^{-1}
4	328.15	6.82		
5	333.15	8.52	9.97 ± 1.02	-3.51 ± 1.02 x 10^{-1}
6	333.15	11.40		
7	338.15	9.83	8.52 ± 0.93	-3.68 ± 1.09 x 10^{-1}
8	338.15	7.21		

[a]Conditions: [cat] : [PPC] = 1 : 100, where [PPC] = 0.3 M in neat PO. Reactions were monitored by in-situ IR, subsequently conversion of PPC to PC was determined by ^{1}H NMR spectroscopy and no polyether linkages were detected. [b]Errors are reported as x ±Δx = σ/√n.

Interpolation of [CO_2] (M) from previously reported data

Due to the limited studies investigating CO_2 solubility in PO, no data was available for the relevant pressures of 2-30 bar CO_2 at 50 °C. CO_2 concentrations for experimentally applied pressures were therefore obtained by interpolation of the data reported by Tassaing and co-workers in 2012.[2] The experimentally determined CO_2 concentrations in PO at 40 °C, 70 °C and 100 °C were kindly provided by Tassaing and co-workers and are shown in Tab. S3-5.

The first objective of the extrapolation was to estimate CO_2 concentrations from the reported data at each of the three available temperatures. A function was fitted to each set of data and subsequently used to calculate the approximate CO_2 concentrations at the experimentally relevant pressures at 40 °C, 70 °C and 100 °C (Fig. S30, Tab. S3-5). The data points were plotted *vs* temperature and a function fitted to each set of data points, i.e. describing the change in concentration of CO_2 in PO over the range of 40 °C- 100 °C when X bar pressure are applied to the system, where X = 2 – 30 bar CO_2. Using the function describing each set of data point, CO_2 concentrations at 50 °C were estimated. The obtained values are reported in Tab. S7.

Tab. S3 [CO_2] concentration in PO with varying CO_2 pressures at 40 °C, data supplied by Foltran et al.[2]

P (MPa)	[CO_2] (M)
0.5	1.27
1	2.75
1.8	4.77
2	5.48
2.6	6.93
3	7.91
4	9.75
4.8	11.82

Tab. S4 [CO_2] concentration in PO with varying CO_2 pressures at 70 °C, data supplied by Foltran et al.[2]

P (MPa)	[CO_2] (M)
0.6	0.62
1	1.34
2	2.81
3	4.33
4	5.75
5	6.86
6	7.36

Tab. S5 [CO_2] concentration in PO with varying CO_2 pressures at 100 °C, data supplied by Foltran et al.[2]

P (MPa)	[CO_2] (M)
1.3	0.63
2	1.37
4.4	3.21
5	3.76
6	4.54
7	5.32

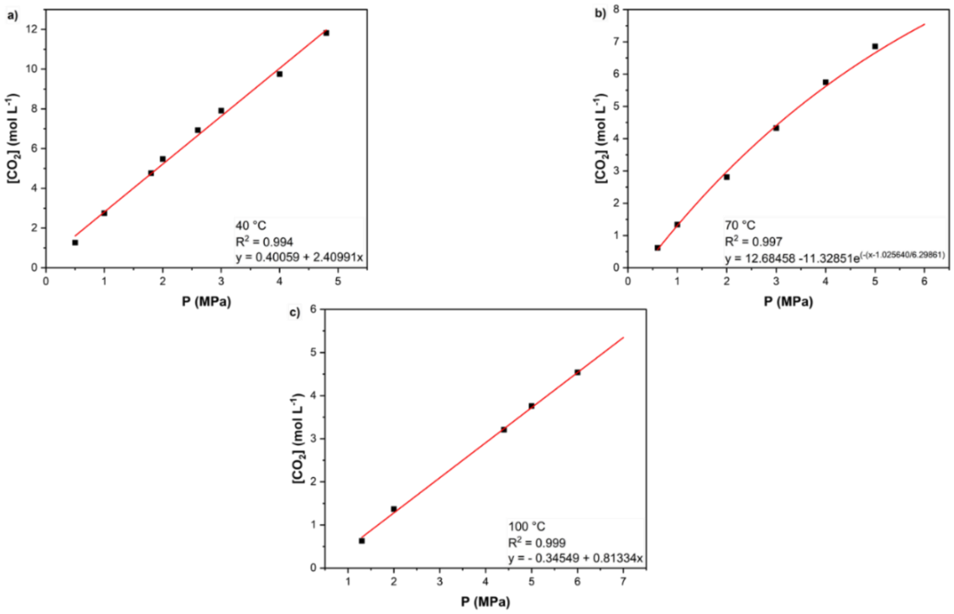

Fig. S30 Plots of [CO_2] (M) vs CO_2 pressure (bar) at (a) 40°C, (b) 70 °C and (c) 100 °C, with fitted trendlines.

Tab. S6 CO_2 concentration at pressures between 2 and 30 bar at 40°C, 70 °C and 100 °C. The shown data was obtained by extrapolation from graphs shown in Fig. S5.

P (bar)	$[CO_2]$ (M) at P = X at 40 °C	$[CO_2]$ (M) at P = X at 70 °C	$[CO_2]$ (M) at P = X at 100 °C
**4**	1.36	0.17	0.15
**5**	1.27	0.37	0.06
**7**	2.09	0.75	0.22
**10**	2.75	1.34	0.47
**12**	3.29	1.67	0.63
**15**	4.02	2.18	0.87
**20**	5.48	2.81	1.37
**25**	6.43	3.7	1.69
**30**	7.91	4.33	2.09

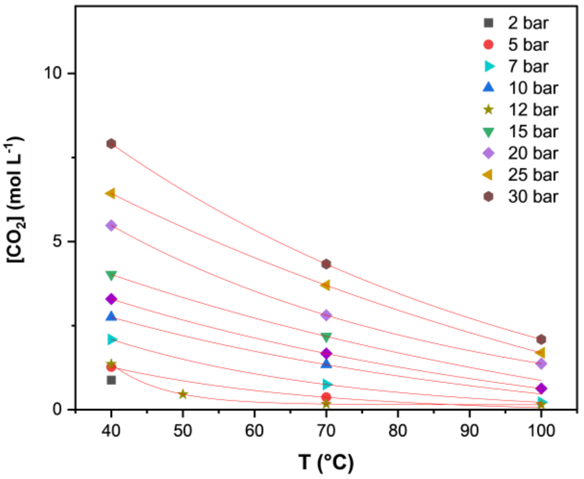

Fig. S31 Plot of $[CO_2]$ (M) vs temperature at P = 2-30 bar. Data taken from Tab. S6, exponential decay trendlines fitted for each pressure.

Tab. S7 [CO_2] (M) at pressures between 2 and 30 bar, obtained by extrapolation of data shown in Fig. S6.

P (bar)	[CO_2] (M)
2*	0.37
4	0.81
5	0.859
7	1.5
9*	1.92
10	2.2
12	2.67
15	3.34
20	4.4
25	5.43
30	6.53

* Values calculated from slope of Graph shown in Fig. S32

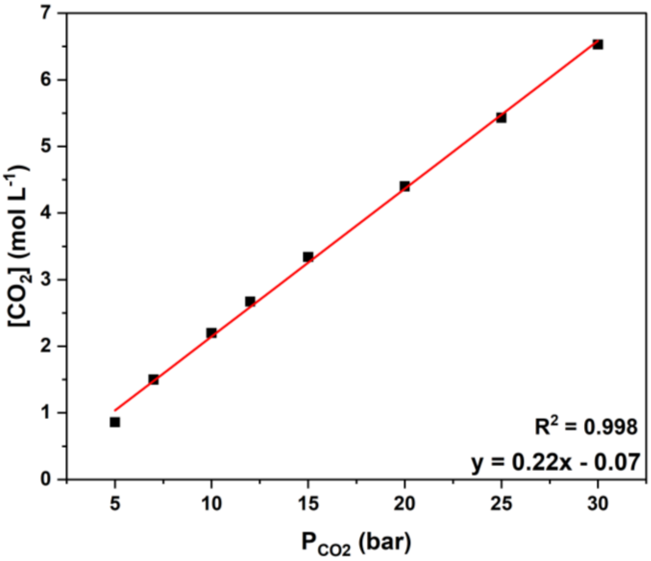

Fig. S32 Linear increase of [CO_2] with CO_2 pressure at 50°C. Data obtained by interpolation as outlined in Tab. S3-7.

Kinetic Data

Tab. S8 Dependence of Activity and Selectivity on CO_2 pressure and CO_2 concentration. All kinetic experiments were performed under a continuous flow of CO_2.[a]

Entry	P_{CO2} / bar	[CO_2] / M	Time / h	Conversion / %	CO_2 / %[b]	PPC / %[c]	TON[d]	TOF / h^{-1} [e]	k_{obs} 10^{-5}/ s^{-1} [f]	M_n [Đ] / g mol^{-1} [g]
1	2	0.37	5.8	16	>99	74	634	109	0.74	2400 [1.12]*
2	2	0.37	4.2	15	98	69	611	144	0.51	2100 [1.09]
3	4	0.81	5	23	>99	88	930	186	1.72	5300 [1.04]
4	4	0.81	3.7	22	>99	91	879	240	1.63	-
5	5	0.86	5	30	99	89	1204	234	1.66	5300 [1.05]*
6	5	0.86	2.5	20	99	95	769	318	2.33	3900 [1.05]
7	7	1.5	3.8	23	>99	94	932	248	1.85	5300 [1.04]
8	7	1.5	3.8	23	>99	95	907	239	1.89	4000 [1.08]
9	9*	1.92	2.8	21	>99	99	851	308	2.35	5000 [1.06]
10	9	1.92	1.7	19	99	96	767	443	3.24	-
11	10	2.2	3.6	27	>99	95	1065	296	2.48	6000 [1.05]*
12	10	2.2	3	24	99	99	960	320	2.27	4900 [1.04]
13	12	2.67	4	33	>99	98	1329	330	3.20	6500 [1.05]*
14	12	2.67	2	21	99	99	838	412	3.31	4400 [1.04]
15	15	3.34	5	45	>99	98	1794	349	3.63	8900 [1.06]*
16	15	3.34	1.5	20	99	> 99	812	497	3.60	2800 [1.06]
17	20	4.4	4	36	>99	99	1457	350	3.43	7100 [1.06]*
18	20	4.4	2	22	>99	98	882	434	3.45	3700 [1.06]
19	25	5.43	4	33	>99	96	1311	326	3.13	5800 [1.05]*
20	25	5.43	2	22	>99	98	882	434	3.05	3700 [1.04]
21	30	6.53	3	34	>99	97	1368	410	3.76	8100 [1.04]*
22	30	6.53	2	23	>99	99	910	404	3.41	3500 [1.09]

[a] Reaction conditions: catalyst (0.025 mol%, 3.6 mM), PO (6 mL, 14.3 M), *trans*-1,2-cyclohexanediol (0.5 mol%, 71 mM) under static CO_2 pressure. [b]CO_2 uptake was calculated by dividing the sum of integrals for polycarbonate and cyclic carbonate against the sum of integrals for polycarbonate, cyclic carbonate and polyether. [c]Polymer selectivity was determined by dividing the sum of integrals for polycarbonate and polyether against the sum of integrals for polycarbonate, cyclic carbonate and polyether. [d]TON was determined by dividing the moles of epoxide consumed (PO: determined by comparison of the sum of integrals by ^{1}H NMR spectroscopy of PPC (4.92 ppm, 1H), PC (4.77 ppm, 1H) and PPO (3.46-3.64 ppm, 3H) against mesitylene (0.25 mol%, 36 mM) as an internal standard;[e]Turnover frequency (TOF) was calculated by dividing the Turnover number (TON) against time.. [f]k_{obs} determined as the gradient of the plot of $\ln[PO]_t/[PO]_0$ *vs.* time . [g]Determined by GPC in THF using narrow dispersity polystyrene standards. Samples labelled with an asterisk (*) were analyzed using a N Agilent LC1260 Infinity II System, see materials for more details.

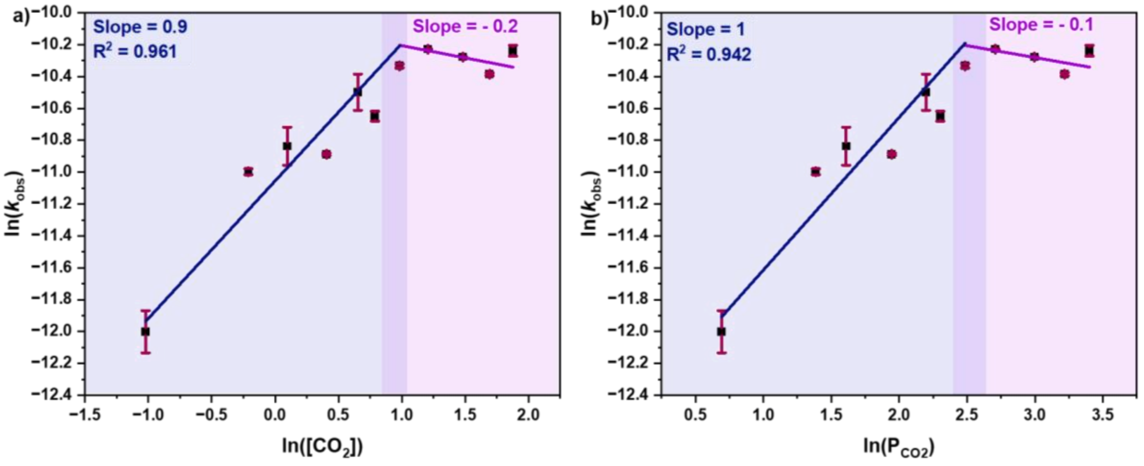

Fig. S33 a) ln(k_{obs}) vs ln(CO_2) b) ln(k_{obs}) vs ln(P_{CO2}).

Tab. S9 Dependence of Activity and Selectivity on catalyst concentration at 5 bar CO_2 pressure.[a]

Entry	Loading cat:PO	[cat] /M	Time / h	Conversion / %	TON[b]	CO_2 / %[c]	PPC / %[d]	TOF / h^{-1} [e]	k_{obs} 10^{-5}/ s^{-1} [f]	M_n [Đ] / g mol^{-1} [g]
1	1:1000	0.143	1	30	301	>99	81	320	6.92	2000 [1.10]
2	1:2000	0.00715	5	36	724	>99	72	147	4.07	3000 [1.07]*
3	1:4000	0.00375	5	30	1204	99	89	234	1.58	5300 [1.05]*
4	1:6000	0.00238	6	15	93	>99	93	887	0.64	5100 [1.04]
5	1:8000	0.00179	8	22	1759	>99	88	228	0.84	8500 [1.05]*

[a] Reaction conditions: PO (6 mL, 14.3 M), *trans*-1,2-cyclohexanediol (20 equiv. vs catalyst), 20 bar CO_2, 50 °C. [b]Turnover number (TON) was determined by dividing the moles of epoxide consumed (PO: determined by comparison of the sum of integrals by ^{1}H NMR spectroscopy of PPC (4.92 ppm, 1H), PC (4.77 ppm, 1H) and PPO (3.46-3.64 ppm, 3H) against mesitylene (0.25 mol%, 36 mM) as an internal standard; [c]CO_2 uptake was calculated by dividing the sum of integrals for polycarbonate and cyclic carbonate against the sum of integrals for polycarbonate, cyclic carbonate and polyether. [d]Polymer selectivity was determined by dividing the sum of integrals for polycarbonate and polyether against the sum of integrals for polycarbonate, cyclic carbonate and polyether. [e]Turnover frequency (TOF) was calculated by dividing the TON against time. [f]k_{obs} determined as the gradient of the plot of ln$[PO]_t/[PO]_0$ vs time, [g]Determined by GPC in THF using narrow dispersity polystyrene standards.

Tab. S10 Dependence of Activity and Selectivity on catalyst concentration at 20 bar CO_2 pressure.[a]

Entry	Loading cat:PO	[cat] /M	Time / h	Conversion / %	TON[b]	CO_2 / %[c]	PPC / %[d]	TOF / h^{-1} [e]	k_{obs} 10^{-5}/ s^{-1} [f]	M_n [Đ] / g mol^{-1} [g]
1	1:2000	0.00715	1.5	29	580	>99	98	348	10.0	3400 [1.06]*
2	1:4000	0.00375	4	36	1457	>99	99	350	3.43	7100 [1.06]*
3	1:6000	0.00238	4	30	1823	>99	> 99	431	2.54	7900 [1.06]*
4	1:8000	0.00179	7	27	2183	>99	98	319	1.40	10300 [1.06]*
5	1:10000	0.00143	14	37	3703	>99	99	273	1.16	19500 [1.05]*

[a] Reaction conditions: PO (6 mL, 14.3 M), *trans*-1,2-cyclohexanediol (20 equiv. vs catalyst), 20 bar CO_2, 50 °C. [b]Turnover number (TON) was determined by dividing the moles of epoxide consumed (PO: determined by comparison of the sum of integrals by ^1H NMR spectroscopy of PPC (4.92 ppm, 1H), PC (4.77 ppm, 1H) and PPO (3.46-3.64 ppm, 3H) against mesitylene (0.25 mol%, 36 mM) as an internal standard; [c]CO_2 uptake was calculated by dividing the sum of integrals for polycarbonate and cyclic carbonate against the sum of integrals for polycarbonate, cyclic carbonate and polyether. [d]Polymer selectivity was determined by dividing the sum of integrals for polycarbonate and polyether against the sum of integrals for polycarbonate, cyclic carbonate and polyether. [e]Turnover frequency (TOF) was calculated by dividing the TON against time. [f]k_{obs} determined as the gradient of the plot of $\ln[PO]_t/[PO]_0$ vs time, [g]Determined by GPC in THF using narrow dispersity polystyrene standards.

Tab. S11 Dependence of Activity and Selectivity on PO concentration at 5 bar. [a]

Entry	Loading cat:PO	[PO] / M	Time / h	Conversion / %	TON[b]	CO_2 / %[c]	PPC / %[d]	TOF / h^{-1} [e]	k_{obs} 10^{-5}/ s^{-1} [f]	M_n [Đ] / g mol^{-1} [g]
1	1:1000	3.6	17	57	571	>99	55	35	1.48	1400 [1.12]*
2	1:2000	7.2	22	75	1505	>99	59	67	1.64	3800 [1.15]*
3	1:2500	8.9	6	33	815	>99	90	136	2.17	3900 [1.05]
4	1:3000	10.7	5.5	30	896	>99	85	163	1.48	2200 [1.06]
5	1:4000	14.3	5	20	740	99	95	341	2.33	5300 [1.05]*

[a] Reaction conditions: PO diluted with diethyl carbonate (6 mL), *trans*-1,2-cyclohexanediol (20 equiv. vs catalyst), 20 bar CO_2, 50 °C. [b]Turnover number (TON) was determined by dividing the moles of epoxide consumed (PO: determined by comparison of the sum of integrals by ^1H NMR spectroscopy of PPC (4.92 ppm, 1H), PC (4.77 ppm, 1H) and PPO (3.46-3.64 ppm, 3H) against mesitylene (0.25 mol%, 36 mM) as an internal standard; [c]CO_2 uptake was calculated by dividing the sum of integrals for polycarbonate and cyclic carbonate against the sum of integrals for polycarbonate, cyclic carbonate and polyether. [d]Polymer selectivity was determined by dividing the sum of integrals for polycarbonate and polyether against the sum of integrals for polycarbonate, cyclic carbonate and polyether. [e]Turnover frequency (TOF) was calculated by dividing the TON against time. [f]k_{obs} determined as the gradient of the plot of $\ln[PO]_t/[PO]_0$ vs time, [g]Determined by GPC in THF using narrow dispersity polystyrene standards.

Tab. S12 Dependence of Activity and Selectivity on PO concentration at 20 bar.[a]

Entry	Loading cat:PO	[PO] / M	Time / h	Conversion / %	TON[b]	CO_2 / %[c]	PPC / %[d]	TOF / h^{-1} [e]	k_{obs} 10^{-5}/ s^{-1} [f]	M_n [Đ] / g mol^{-1} [g]
1	1:1000	3.6	16.5	83	823	>99	90	50	3.14	2800 [1.14]*
2	1:2000	7.2	21	88	1759	>99	96	84	3.06	6500 [1.13]*
3	1:2500	8.9	8	21	1323	>99	96	165	2.73	4900 [1.06]
4	1:3000	10.7	7	43	1281	>99	98	178	2.54	6700 [1.11]
5	1:4000	14.3	4	36	1457	>99	99	350	3.43	7100 [1.06]*

[a] Reaction conditions: PO diluted with diethyl carbonate (6 mL), *trans*-1,2-cyclohexanediol (20 equiv. vs catalyst), 20 bar CO_2, 50 °C. [b]Turnover number (TON) was determined by dividing the moles of epoxide consumed (PO: determined by comparison of the sum of integrals by ^{1}H NMR spectroscopy of PPC (4.92 ppm, 1H), PC (4.77 ppm, 1H) and PPO (3.46-3.64 ppm, 3H) against mesitylene (0.25 mol%, 36 mM) as an internal standard; [c]CO_2 uptake was calculated by dividing the sum of integrals for polycarbonate and cyclic carbonate against the sum of integrals for polycarbonate, cyclic carbonate and polyether. [d]Polymer selectivity was determined by dividing the sum of integrals for polycarbonate and polyether against the sum of integrals for polycarbonate, cyclic carbonate and polyether. [e]Turnover frequency (TOF) was calculated by dividing the TON against time. [f]k_{obs} determined as the gradient of the plot of ln$[PO]_t/[PO]_0$ vs time, [g]Determined by GPC in THF using narrow dispersity polystyrene standards.

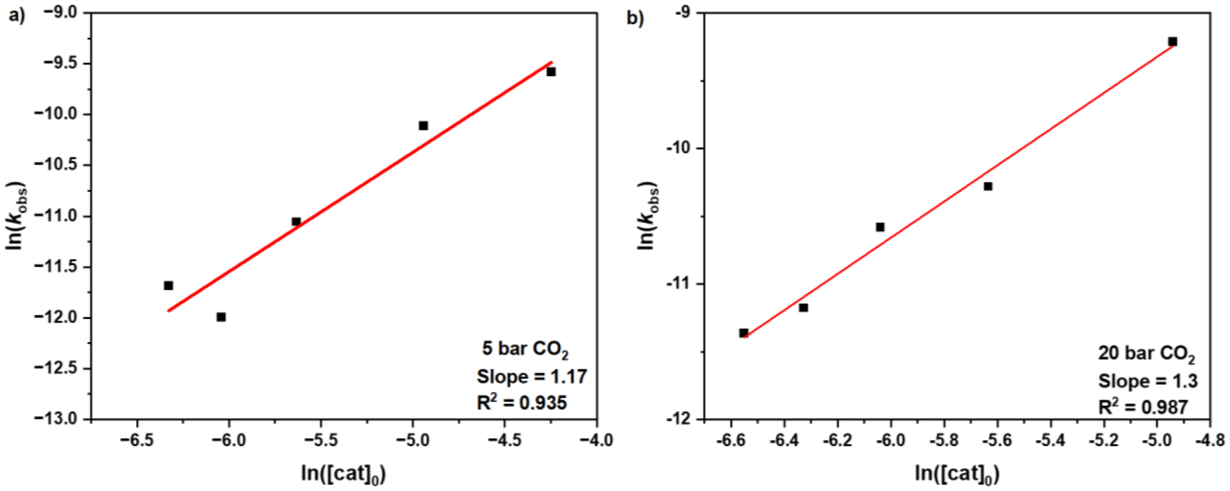

Fig. S34 a) ln(k_{obs}) vs ln([cat]) at 5 bar b) ln(k_{obs}) vs ln([cat]) at 20 bar

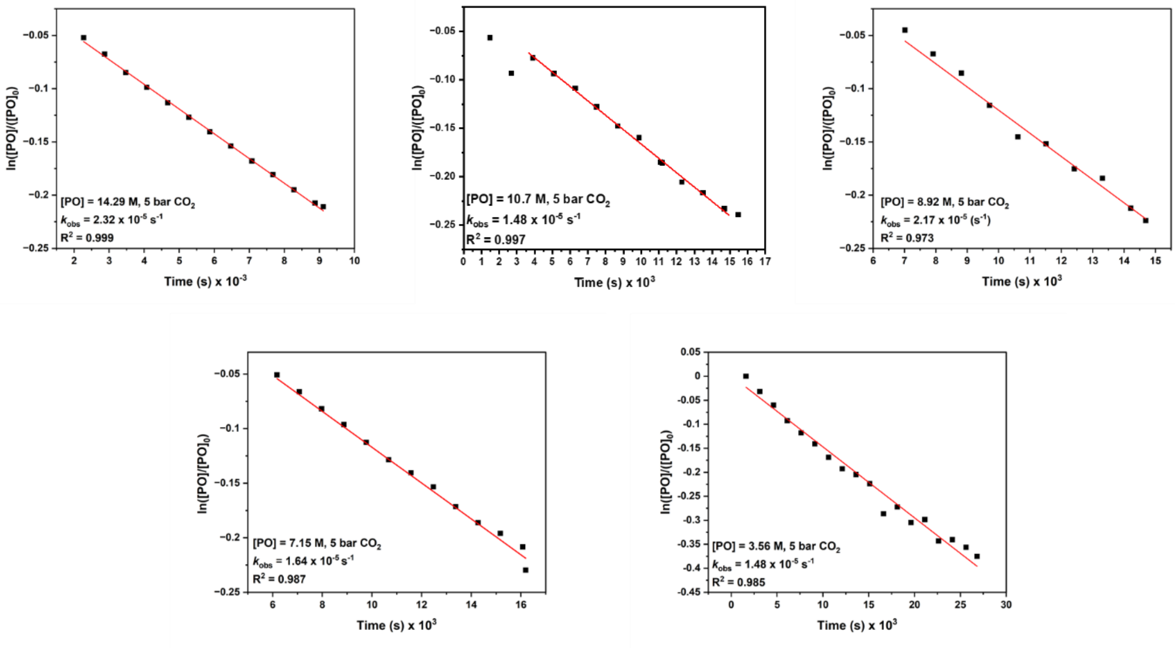

Fig. S35 Semilogarithmic plots for ln([PO]/[PO]$_0$) vs time for all tested PO concentrations at 5 bar CO_2 pressure. A linear dependence was observed at all concentrations indicating a first order dependence on [PO] in all experiments.

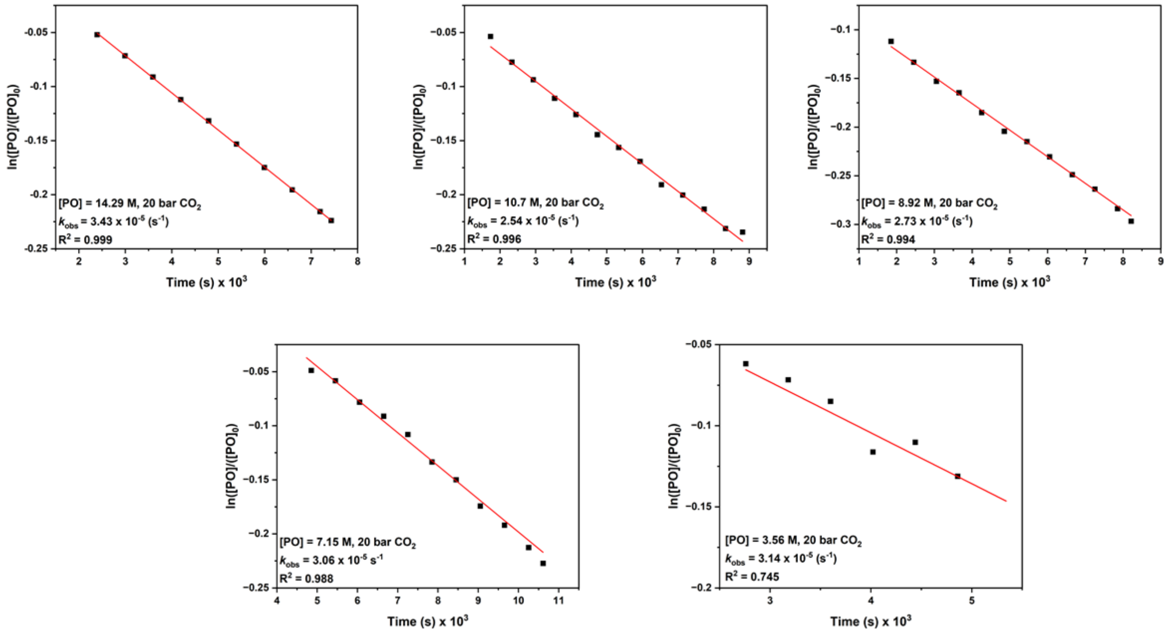

Fig. S36 Semilogarithmic plots for ln([PO]/[PO]$_0$) vs time for all tested PO concentrations at 20 bar CO_2 pressure. A linear dependence was observed at all concentrations indicating a pseudo first order dependence on [PO] in all experiments.

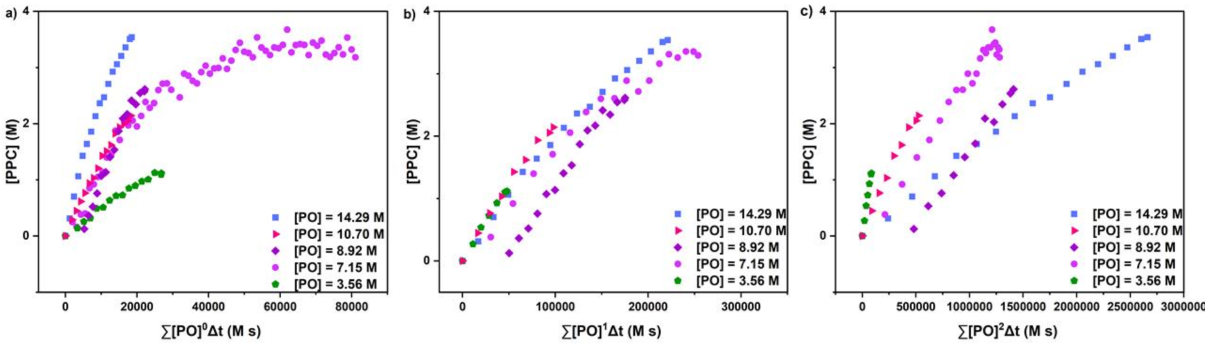

Fig. S37 VTNA plot showing the best fit for an order of (b) 1 at 5 bar, and inferior fits for orders of (a) 0 and (c) 2.[3]

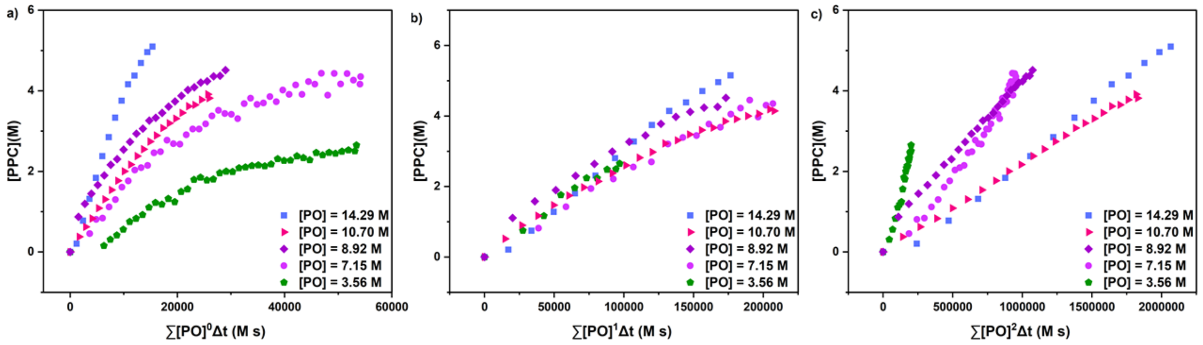

Fig. S38 VTNA plot showing the best fit for an order of 1 at 20 bar (b), and inferior fits for an order of (a) 0 (c) 2. [3]

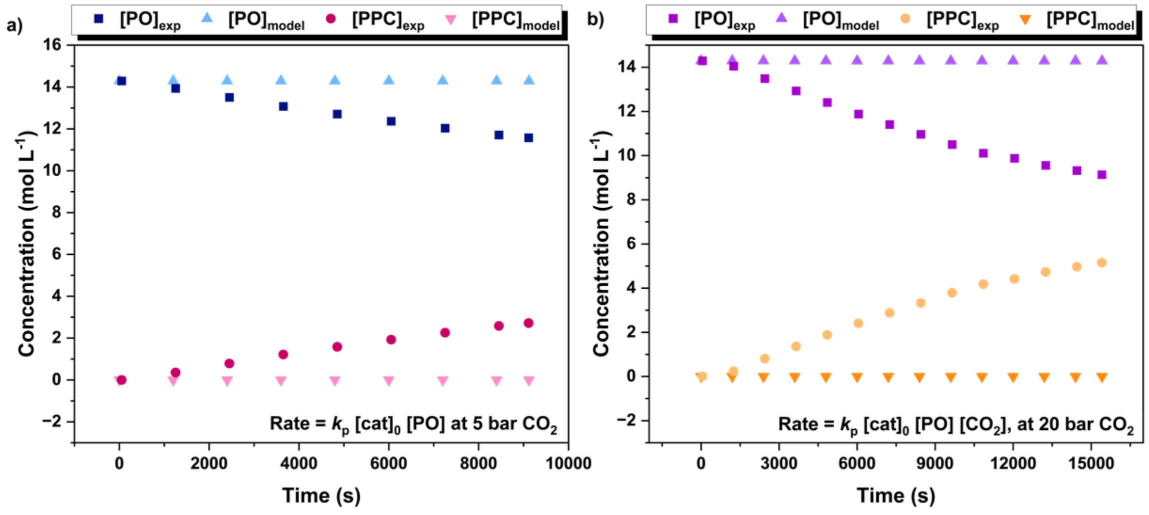

Fig. S39 Graphs illustrating inferior fits for a COPASI model when a) removing CO_2 dependence for the rate law at 5 bar where modelled rate = k_p $[cat]_0$ $[PO]_0$, where k_p = 6.51 × 10^{-3} M^{-3} s^{-1}, $[cat]_0$ = 3.57 mM, $[PO]_0$ = 14.29 M and b) adding a CO_2 dependence for a model at 20 bar, modelled rate = k_p $[cat]_0$ $[PO]_0$ $[CO_2]$, where k_p =0.0022 M^{-2} s^{-1}, $[cat]_0$ = 3.57 mM, $[PO]_0$ = 14.29 M, $[CO_2]$ = 4.4 M.[4]

Table 13 Data showing how varying the pressure and [CO_2] impacts the rate coefficient (k_{obs}), [carbonate], [alkoxide] and K_{eq} according to the unified rate law.

P_{CO2} /bar[a]	[CO_2] /M[b]	k_{obs} / s^{-1} x 10^{-5}[c]	[carbonate] / mM[d]	[alkoxide] / mM[e]	K_{eq} / M^{-1} × 10^{-1}[f]
2	0.37	0.63 ± 8.2 × 10^{-2}	0.65 ± 0.1	2.92 ± 0.1	0.60 ± 1.6
4	0.81	1.68 ± 3.2 × 10^{-2}	1.75 ± 0.1	1.82 ± 0.1	1.18 ± 0.1
5	0.86	1.99 ± 2.6 × 10^{-1}	2.08 ± 0.3	1.49 ± 0.3	1.62 ± 0.4
7	1.50	1.90 ± 1.4 × 10^{-2}	1.98 ± 1.4	1.59 ± 1.4	0.83 ± 0.1
9	1.92	2.80 ± 3.2 × 10^{-1}	2.92 ± 0.4	0.66 ± 0.4	2.32 ± 1.4
10	2.20	2.38 ± 7.42 × 10^{-2}	2.48 ± 0.2	1.09 ± 0.2	1.03 ± 0.2

[a]Experimentally applied CO_2 pressure. [b]Calculated from data supplied by Ref[2], see SI spreadsheet for details. [c]Averages of n = 2 runs, with an error of $\Delta x = \sigma/\sqrt{n}$. [d]Determined using $rate_P/rate_{max} \times rate_{max}$ = $[carbonate]_P$, using average k_{obs} values and $rate_{max}$= 3.43 x 10^{-5} s^{-1} [e] Calculated from $[cat]_0$ – [carbonate] = [alkoxide]. [f] Calculated from $K_{eq} = \frac{[carbonate]_P}{[alkoxide]_P\,[CO2]}$ (SI Spreadsheet).

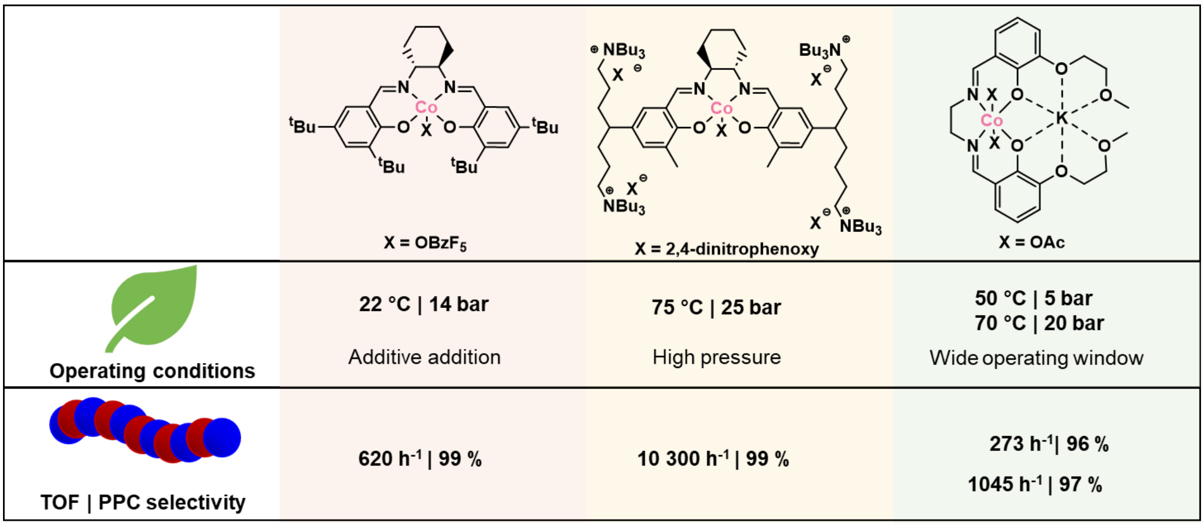

Fig. S40 Comparison of the three field-leading PO/CO_2 ROCOP catalysts: cobalt salen[5], tethered cobalt salen[6] and 1. TOF and PPC selectivity are shown for each catalyst at the conditions reported, closest to operation conditions, which would be expected to minimize GHG emissions associated with the reaction (chosen as highest temperatures, lowest pressure, maximum selectivity).

Backbiting mechanism and barrier determination

Backbiting from the alkoxide intermediate

We propose that the only backbiting reaction occurs from the alkoxide intermediate (Figure S41a). The mechanism proposed is supported both by the current experimental results and a previously reported DFT study using the 'closed' catalyst.[7]

This paper demonstrates that CO_2 insertion is an equilibrium, which saturates above 12 bar CO_2 at 50 °C. In the saturation regime (plateau regime), a steady state is reached and the [alkoxide] is very low. No cyclic carbonate (PC) formation occurs in this regime. In the linear (pre-equilibrium regime), the ratio of [alkoxide]:[carbonate] correlates to the experimentally determined selectivity of PC:PPC (Figure S42). The rate of PC formation directly relates to [alkoxide], thereby supporting that the pathway to PC formation is backbiting from the alkoxide intermediate.

Our prior DFT investigation into the formation of PC using the 'closed' Co(III)K(I) catalyst, showed that the barrier to backbiting from the alkoxide intermediate was significantly lower ($\Delta G^{\ddagger}_{calc}$ (Alkoxide) = 22.4 kcal mol^{-1}) compared to the backbiting barrier from the carbonate intermediate ($\Delta G^{\ddagger}_{calc}$ (Carbonate) = 31.9 kcal mol^{-1}).[7] We expect the equivalent barriers to be lower for the 'open' catalyst but we expect the relative magnitudes of the alkoxide and carbonate barriers to remain very similar. The previously reported DFT investigation further supports the proposed mechanism, in which backbiting occurs from the alkoxide intermediate (Figure S41).

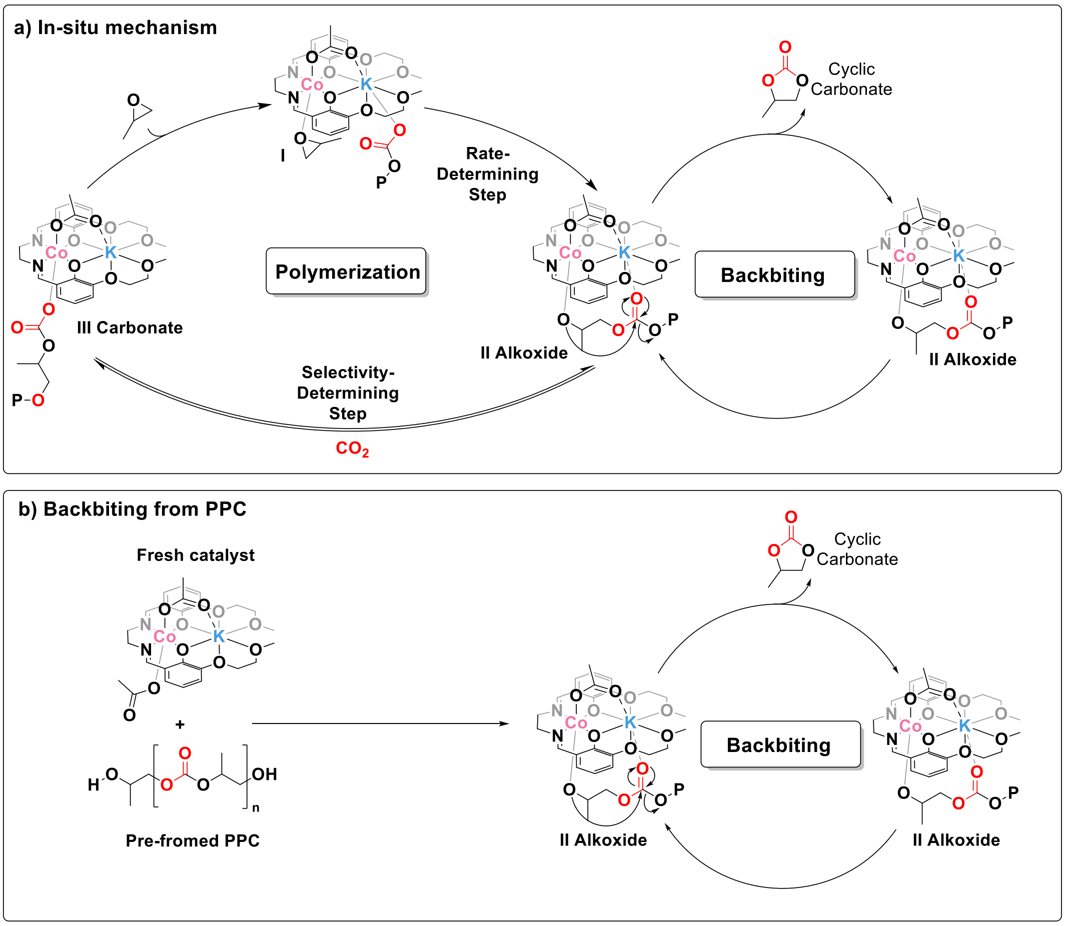

Fig. S41 a) Polymerization mechanism and backbiting mechanism from the alkoxide b) Backbiting barrier (mechanism) determination using hydroxyl-end capped PPC to form cyclic carbonate.

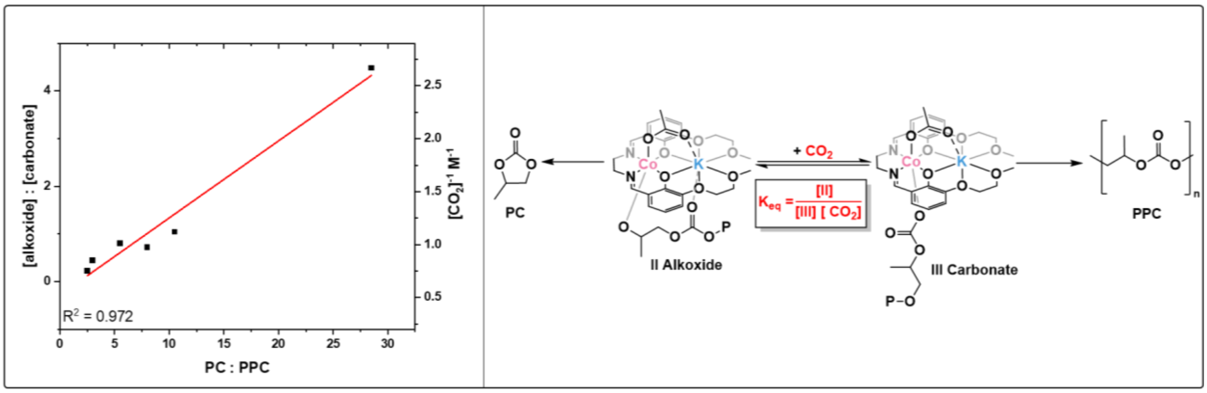

Fig. S42 Linear correlation of the [alkoxide] : [carbonate] ratio with the PC:PPC product ratio, illustrating that the backbiting mechanism proceeds from the alkoxide rather than the carbonate intermediate.

Backbiting from pre-formed PPC as an appropriate model for the backbiting from the alkoxide

In the experiments to determine the experimental alkoxide ‘back-biting’ barrier we do not use the very low percentage of PC formed during polymerization to determine the barrier as there are very large errors associated with using such low percentage conversions. Rather, we use a propylene carbonate polymer with a hydroxyl end-group. This polymer reacts with the catalyst to form the same alkoxide intermediate (Figure S41b) as is present during polymerization (Figure S41a). In contrast to polymerization, there is no carbon dioxide present and this allows the (polymer)-alkoxide intermediate to undergo cyclization to form only propene carbonate (PC). The only product of the polymer reaction is the cyclic carbonate. Therefore, by using the data from these back-biting experiments (temperature dependence of rates), the barrier to back-biting from the alkoxide intermediate can be estimated more accurately. Importantly, during polymerization, the carbon dioxide is present and so there is a very low concentration of alkoxide intermediate under most conditions

A series of experiments to determine that the PC formed from the alkoxide intermediate was considered. If the catalyst was highly regioselective (Head-to-tail enchainment only), then making PPC from a single enantiomer of PO would result in retained stereochemistry for the PC formed from the alkoxide intermediate (whereas the carbonate intermediate would result in inverted stereochemistry). However, when the regioselectivity for the catalyst was determined, it showed a regio-random structure: 20:60:20 (head-head:head-tail:tail-tail) (PPC prepared at 50 °C, 1:20:10,000, cat:CTA:PO at 20 bar CO_2 and regiochemistry measured using quantitative ^{13}C NMR) (Figure S43). The lack of regiocontrol means that stereochemical inversion studies are not going to reveal which intermediate is responsible for the chain back-biting.

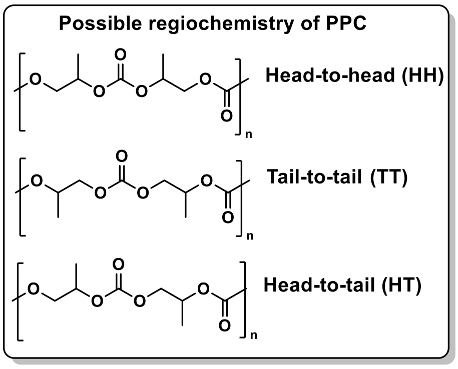

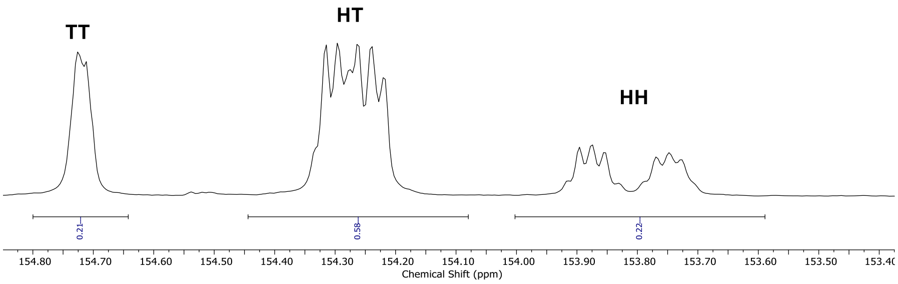

Figure S43 Determination of PPC regiochemistry (with assignments illustrated) using quantitative ^{13}C NMR spectroscopy. PPC synthesised using catalyst 1 at 50 °C, 20 bar CO_2 pressure (Table 2, entry 10)

Estimation of K_{eq} of previously reported Zinc BDI complexes

The equilibrium constant K_{eq} was estimated for two previously reported zinc BDI complexes (Fig. S40), using the same method as outlined for the here reported Co(III)K(I) (1) catalyst. The used experimental values and all calculations can also be found in the supplementary excel file.

Initially, carbonate concentration was calculated from the following expression $\frac{\text{rate}_P}{\text{rate}_{max}} \times \text{carbonate}_{max} = [\text{carbonate}]_P$. The rate at any given pressure, rate_p, was taken directly from the published supplementary information, specifically from Tab. S2 for the monometallic complex[8] shown in Fig. S4 a and from Tab. S2[9] for the dinculear catalyst shown in Fig. S40 b. It is important to note that in both publications the catalyst concentration and temperature at which the reactions were run are not unambiguously identifiable. The here reported carbonate and therefore K_{eq} are therefore estimates only. Alkoxide concentration was calculated by subtracting the calculated carbonate concentration form the assumed, initial catalyst concentration.

The equilibrium constant was then calculated using the calculated concentration for both alkoxide and carbonate, as well as the reported CO_2 concentration (SI spreadsheet details), corresponding the experimentally applied CO_2 pressure, according to: $K_{eq} = \frac{[\text{carbonate}]}{[\text{alkoxide}]\,[\text{CO2}]}$.

a) b)

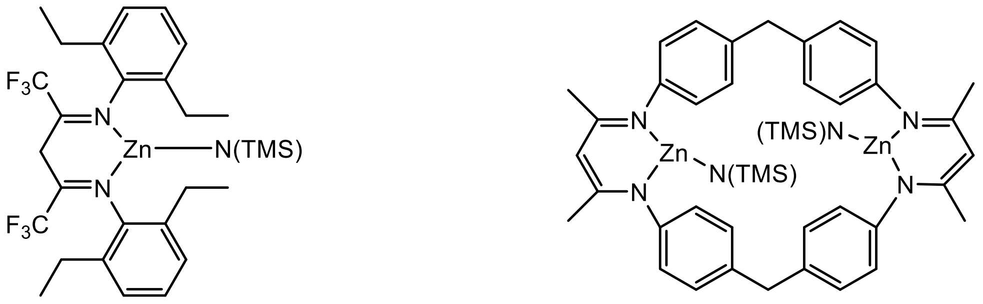

Shift in rate at 5 - 10 bar CO_2 pressure

$\text{Rate}_{3bar} = [\text{cat}]^1\ [\text{CHO}]^1\ [\text{CO}_2]^1$

$\text{Rate}_{30bar} = [\text{cat}]^1\ [\text{CHO}]^1\ [\text{CO}_2]^0$

Shift in rate at 25 bar CO_2 pressure

$\text{Rate}_{5\text{-}25bar} = [\text{cat}]^1\ [\text{CHO}]^0\ [\text{CO}_2]^1$

$\text{Rate}_{25\text{-}45bar} = [\text{cat}]^1\ [\text{CHO}]^1\ [\text{CO}_2]^0$

Fig. S44 Structure and rate laws reported for a) Mononuclear Zinc BDI catalyst for the CHO/CO_2 ROCOP[8] and b) Dinuclear zinc BDI catalyst for the CHO/CO_2 ROCOP[9].

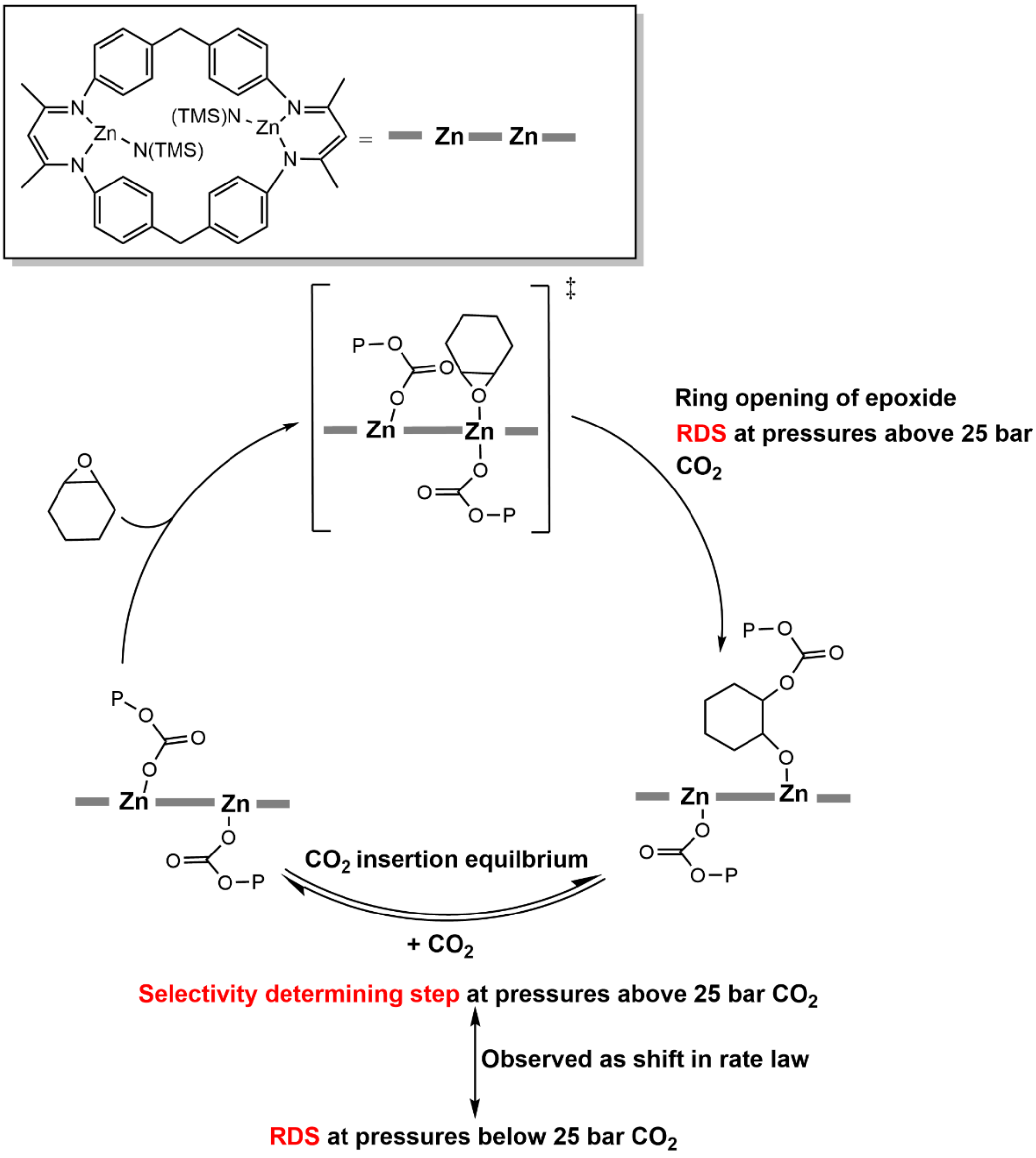

Fig. S45 Proposed mechanisms for the previously reported dinuclear zinc BDI CHO/CO_2 ROCOP catalyst, taking into account the here determined CO_2 insertion equilibrium and the previously reported ret laws (Fig. S40).[9]

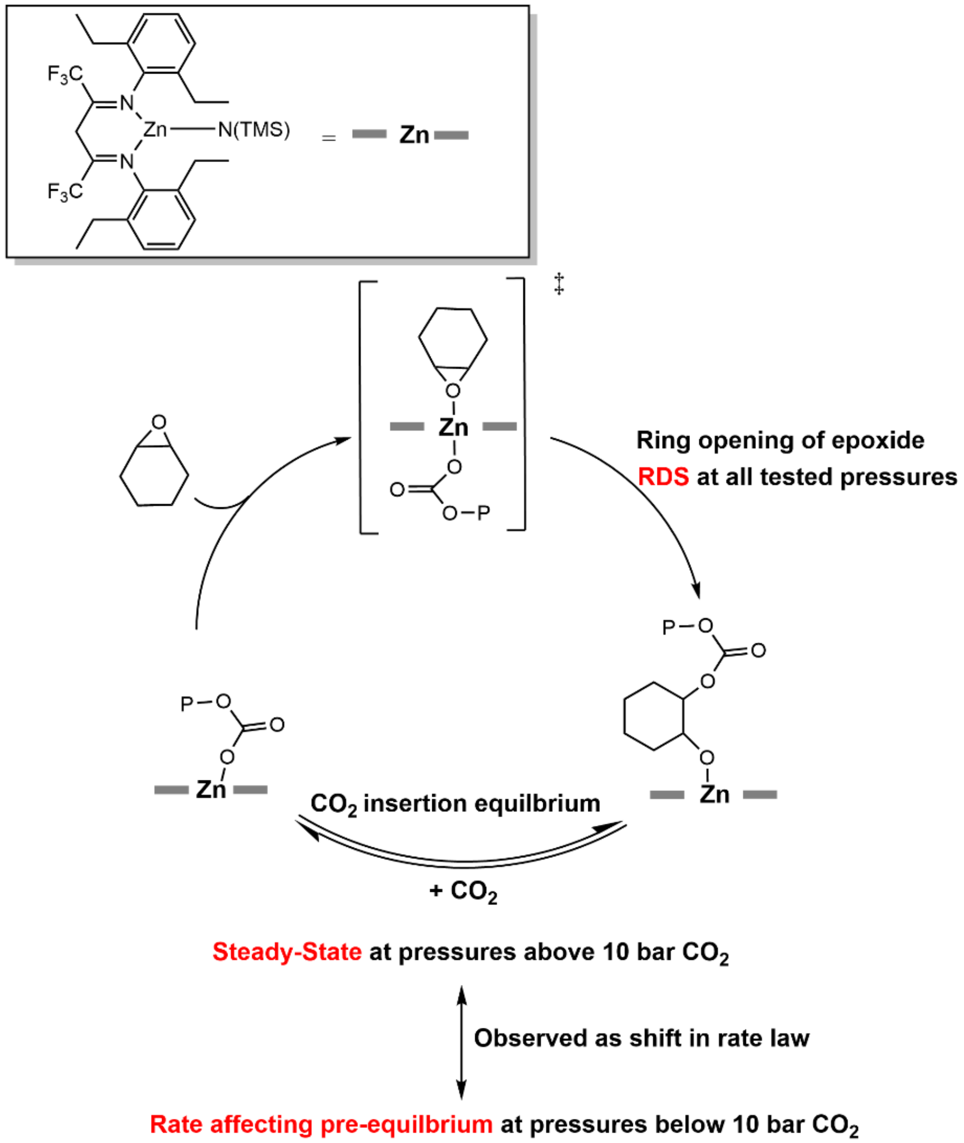

Fig. S46 Proposed mechanisms for the previously reported monometallic zinc BDI CHO/CO_2 ROCOP catalyst, taking into account the here determined CO_2 insertion equilibrium and the previously reported ret laws (Fig. S40).[8]

COPASI Modelling

All kinetic modelling was performed using COPASI v4.40, using the rate equations and parameters outlined below[4]

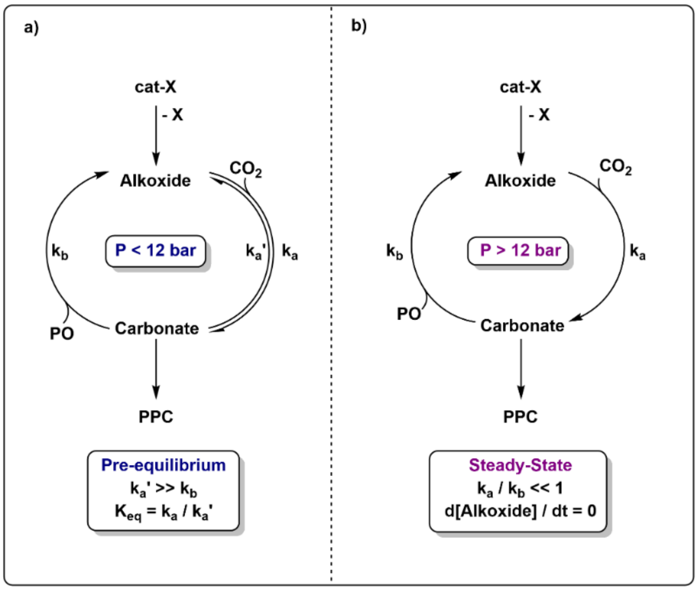

Fig. S47 Illustration of (a) the Pre-equilibrium approximation and (b) the Steady-State approximation assumed for the PO/CO_2 ROCOP using 1 at low P < 12 bar and P > 12 bar, respectively.

Tab. S14 Parameters and rate equations used for COPASI models.

Entry	Model	Reaction(s)	Rate Law(s)	Parameters
1	Experimental Rate Law 5 bar (Fig. 6a)	Catalyst + PO + CO_2 → PPC + Catalyst	k_p*Catalyst*CO_2*PO	Catalyst = 0.00375 mol l^{-1}, fixed;<br>PO =14.29 mol l^{-1}, reactions, CO_2 = 0.86 mol l^{-1}, fixed; PPC = 0 mol l^{-1}, reactions; k_p = 0.00757 l^2 mol^{-2} s^{-1}, fixed
2	Experimental Rate Law 20 bar (Fig. 6b)	Catalyst + PO + CO_2 → PPC + Catalyst	k_p*Catalyst*PO	Catalyst = 0.00375 mol l^{-1}, fixed; PO =14.29 mol l^{-1}, reactions; PPC = 0 mol l^{-1}, reactions; k_p = 0.00961 l^2/ (mol^2*s), fixed
3	Experimental Rate Law 5 bar without CO_2 dependence (Fig. S44a)	Catalyst + PO + CO_2 → PPC + Catalyst	k_p*Catalyst*PO	Catalyst = 0.00375 mol l^{-1}, fixed; PO =14.29 mol l^{-1}, reactions; PPC = 0 mol l^{-1}, reactions; k_p = 0.00651 l^2/ mol^{-2} s^{-1}, fixed
4	Experimental Rate Law 20 bar with CO_2 dependence (Fig. S44b)	Catalyst + PO + CO_2 → Catalyst + PPC	k_p*Catalyst*CO_2*PO	Catalyst = 0.00375 mol l^{-1}, fixed; PO =14.29 mol l^{-1}, reactions, CO_2 = 4.4 mol l^{-1}, fixed; PPC = 0 mol l^{-1}, reactions; k_p = 0.0022 l^2 mol^{-2} s^{-1}, fixed
5	Unified Rate Law 5 bar (Fig. 10)	(1) Carbonate + PO → Alkoxide<br>(2) Alkoxide + CO_2 = Carbonate + PPC	(1) K_{eq}*Alkoxide*CO_2<br>(2) k_p*PO*Carbonate	**K_{eq} = 2.46 l mol^{-1}**, fixed;<br>Alkoxide = 0.00375 mol l^{-1}, reactions;<br>CO_2 = 0.86 mol l^{-1}, fixed; k_p = 0.00961 l mol^{-2} s^{-1} , fixed; PO = 14.29 mol l^{-} Carbonate = 0 mol l^{-1}

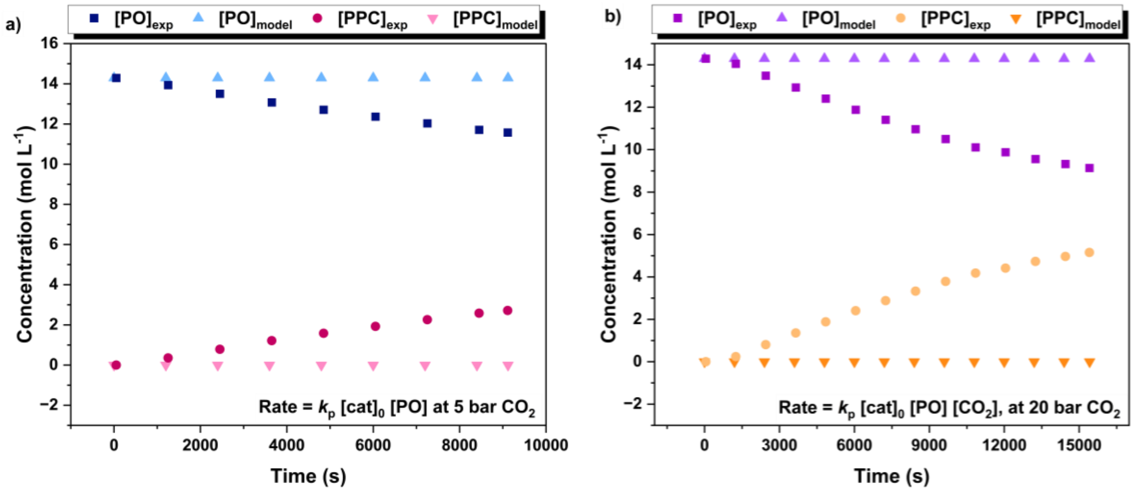

Fig. S48 Concentration vs time data modelled using a rate law without a CO_2 dependence at low pressure and a first order CO_2 dependence at high pressure in COPASI and comparison with experimentally collected concentration vs time data for reactions performed at a) 5 bar CO_2 pressure (Tab. S13, entry 3), where the CO_2 dependence was removed from the modelled rate: rate = k_p $[cat]_0$ $[PO]_0$, b) 20 bar CO_2 pressure (Tab. S13, entry 4), where a CO_2 dependence was added to the modelled rate: rate = k_p $[cat]_0$ $[PO]_0$ $[CO_2]$.[4]

Single X-Ray Crystallography

Tab. S15 Selected bond lengths for Co(III)K(I) (1), Co(III)Na(I) (2) and Co(III)Rb(I) (3).

**Co(III)K(I) (026FF22)**		**Co(III)Na(I) (003FF23)**		**Co(III)Rb(I) (027FF23)**	
**Bond**	**Length (Å)**	**Bond**	**Length (Å)**	**Bond**	**Length (Å)**
Co01—K1	3.7502 (6)	Co01—Na1	3.2816 (8)	Rb1—Co1	3.8749 (4)
Co01—O8	1.9095 (17)	Co01—O8	1.8830 (13)	Rb1—O5	2.8348 (15)
Co01—O5	1.9154 (17)	Co01—O5	1.8910 (14)	Rb1—O6	2.8865 (16)
Co01—O3	1.9297 (17)	Co01—O3	1.9198 (14)	Rb1—O8	2.8720 (16)
Co01—O2	1.9223 (18)	Co01—O2	1.9251 (14)	Rb1—O4^{i}	2.9803 (18)
Co01—N1	1.891 (2)	Co01—N2	1.8832 (17)	Rb1—O7	3.048 (2)
Co01—N2	1.877 (2)	Co01—N1	1.8927 (16)	Rb1—O9	2.8973 (19)
K1—O8	2.7034 (17)	Na1—O8	2.3188 (15)	Rb1—O1	2.844 (2)
K1—O5	2.7569 (17)	Na1—O5	2.3605 (16)	Rb1—O10	3.055 (3)
K1—O9	2.7945 (18)	Na1—O9	2.7651 (16)	Co1—O5	1.9172 (15)
K1—O10	3.019 (2)	Na1—O10	2.4462 (17)	Co1—O8	1.9218 (16)
K1—O6	2.817 (2)	Na1—O6	2.5593 (17)	Co1—O3	1.9249 (16)
K1—O1	2.725 (2)	Na1—O1	2.3079 (17)	Co1—O2	1.9434 (18)
K1—O7	3.025 (3)	Na1—O7	2.474 (2)	Co1—N1	1.8783 (19)
O3—C3	1.291 (3)	O3—C3	1.240 (3)	Co1—N2	1.892 (2)
O2—C1	1.271 (3)	O2—C1	1.282 (3)	O3—C3	1.292 (3)
O4—C3	1.230 (3)	O1—C1	1.234 (3)	O2—C1	1.201 (3)
N1—C7	1.279 (3)	O4—C3	1.239 (3)	O4—C3	1.230 (3)
N1—C5	1.469 (3)	N2—C7	1.279 (3)	N1—C6	1.277 (3)
N2—C19	1.281 (3)	N2—C6	1.474 (2)	N1—C5	1.465 (3)
N2—C6	1.468 (3)	N1—C5	1.466 (3)	N2—C17	1.280 (3)
O1—C1	1.223 (4)	N1—C16	1.279 (3)	N2—C16	1.468 (3)
C3—C4	1.526 (3)	C3—C4	1.541 (3)	C3—C4	1.522 (3)
C1—C2	1.527 (4)	C1—C2	1.521 (3)	C1—C2	1.539 (4)

Tab. S16 Selected angles for Co(III)K(I) (1), Co(III)Na(I) (2) and Co(III)Rb(I) (3).

**Co(III)K(I) (026FF22)**		**Co(III)Na(I) (003FF23)**		**Co(III)Rb(I) (027FF23)**	
**Bond**	**Angle (°)**	**Bond**	**Angle (°)**	**Bond**	**Angle (°)**
O8—Co01—K1	43.42 (5)	O8—Co01—Na1	43.68 (4)	O5—Rb1—Co1	28.12 (3)
O8—Co01—O5	86.54 (7)	O8—Co01—N2	94.66 (7)	O8—Rb1—Co1	28.45 (3)
O8—Co01—O3	89.82 (7)	O8—Co01—N1	179.40 (6)	O1—Rb1—O4^{i}	169.52 (6)
O8—Co01—O2	93.86 (8)	O5—Co01—Na1	45.02 (4)	O3—Co1—O2	176.73 (7)
N1—Co01—K1	136.85 (7)	O5—Co01—O3	85.62 (6)	N1—Co1—Rb1	135.06 (6)
N1—Co01—O8	179.73 (9)	O5—Co01—O2	93.43 (6)	N1—Co1—O5	93.42 (7)
N1—Co01—O5	93.71 (8)	N2—Co01—Na1	135.78 (5)	N1—Co1—O8	178.60 (8)
N2—Co01—K1	134.88 (6)	N2—Co01—O5	179.13 (6)	N1—Co1—N2	85.64 (8)
N2—Co01—O5	178.73 (8)	N2—Co01—O3	94.41 (7)	N2—Co1—Rb1	136.80 (6)
N2—Co01—O3	94.27 (8)	O8—Na1—Co01	34.11 (3)	N2—Co1—O5	179.02 (8)
N2—Co01—O2	85.35 (8)	O5—Na1—Co01	34.52 (4)	N2—Co1—O8	93.57 (8)

Tab. S17 Summary of crystallographic refinement data for complexes 1 – 3.

**Complex**	**Co(III)K(I) (1)**	**Co(III)Na(I) (2)**	**Co(III)Rb(I) (3)**
**Local Code**	026ff22_autored-ff	003ff23_autored-ff	027ff22_autored-ff
**CCDC Deposition Number**	2311958	2311959	2311960
**Crystal Data**			
Chemical formula	$C_{26}H_{32}CoKN_2O_{10}\cdot C_{3.7}H_{7.1}O$	$C_{25.99}H_{31.958}CoN_2NaO_{10}\cdot H_2O$	$C_{26}H_{32}CoN_2O_{10}Rb\cdot$1[C4H10O]
M_r	704.68	632.31	751.05
Crystal system, space group	Monoclinic, $P2_1/c$	Triclinic, $P\bar{1}$	Monoclinic, $P2_1/c$
Temperature (K)	150	150	150
a, *b*, *c* (Å)	11.6891 (1), 11.7621 (1), 23.7980 (1)	8.5451 (3), 10.7932 (3), 15.7576 (5)	11.6141 (1), 11.8573 (1), 23.7892 (1)
a, b, g (°)	91.851 (1)	82.866 (3), 85.918 (3), 80.361 (3)	91.924 (1)
V (Å^3)	3270.24 (4)	1419.81 (8)	3274.21 (4)
Z	4	2	4
Radiation type	Cu *K*a	Cu *K*a	Cu *K*a
m (mm^{-1})	5.78	5.44	6.43
Crystal size (mm)	0.25 × 0.14 × 0.10	0.2 × 0.1 × 0.1	0.50 × 0.13 × 0.08
**Data collection**			
Diffractometer	SuperNova, Dual, Cu at home/near, Atlas	SuperNova, Dual, Cu at home/near, Atlas	SuperNova, Dual, Cu at home/near, Atlas
Absorption correction	Multi-scan *CrysAlis PRO* 1.171.41.117a (Rigaku Oxford Diffraction, 2021) Empirical absorption correction using spherical harmonics, implemented in SCALE3 ABSPACK scaling algorithm.	Multi-scan *CrysAlis PRO* 1.171.42.72a (Rigaku Oxford Diffraction, 2022) Empirical absorption correction using spherical harmonics, implemented in SCALE3 ABSPACK scaling algorithm.	Multi-scan *CrysAlis PRO* 1.171.42.72a (Rigaku Oxford Diffraction, 2022) Empirical absorption correction using spherical harmonics, implemented in SCALE3 ABSPACK scaling algorithm.
T_{min}, T_{max}	0.680, 1.000	0.776, 1.000	0.360, 1.000
No. of measured, independent and observed [I > 2s(I)] reflections	83929, 6811, 6524	14361, 5859, 5638	82791, 6822, 6442
R_{int}	0.038	0.019	0.059
(sin q/l)$_{max}$ (Å^{-1})	0.630	0.630	0.630

Refinement			
$R[F^2 > 2s(F^2)]$, $wR(F^2)$, S	0.048, 0.151, 1.06	0.038, 0.109, 1.04	0.036, 0.094, 1.06
No. of reflections	6811	5859	6822
No. of parameters	436	390	370
No. of restraints	2	1	-
H-atom treatment	H atoms treated by a mixture of independent and constrained refinement	H-atom parameters constrained	H atoms treated by a mixture of independent and constrained refinement
$D\rho_{max}$, $D\rho_{min}$ (e Å^{-3})	2.37, -0.50	1.46, -0.50	1.16, -0.44

Computer programs: *CrysAlis PRO* 1.171.42.72a (Rigaku OD, 2022), olex2.solve 1.5 (Bourhis *et al.*, 2015), *SHELXL* 2018/3 (Sheldrick, 2015), Olex2 1.5 (Dolomanov *et al.*, 2009).

References

(1) Schall, O. F.; Robinson, K.; Atwood, J. L.; Gokel, G. W. Self-assembling nickel clusters form binding sites for alkali metal cations: novel analogs of enolate aggregates. *J. Am. Chem. Soc.* **1993**, *115* (14), 5962-5969. DOI: 10.1021/ja00067a010.

(2) Foltran, S.; Cloutet, E.; Cramail, H.; Tassaing, T. In situ FTIR investigation of the solubility and swelling of model epoxides in supercritical CO_2. *J. Supercrit. Fluids* **2012**, *63*, 52-58. DOI: https://doi.org/10.1016/j.supflu.2011.12.015.

(3) Nielsen, C. D. T.; Burés, J. Visual kinetic analysis. *Chem. Sci.* **2019**, *10* (2), 348-353, 10.1039/C8SC04698K. DOI: 10.1039/C8SC04698K.

(4) Hoops, S.; Sahle, S.; Gauges, R.; Lee, C.; Pahle, J.; Simus, N.; Singhal, M.; Xu, L.; Mendes, P.; Kummer, U. COPASI—a Complex Pathway Simulator. *Bioinformatics* **2006**, *22* (24), 3067-3074. DOI: 10.1093/bioinformatics/btl485.

(5) Cohen, C. T.; Chu, T.; Coates, G. W. Cobalt Catalysts for the Alternating Copolymerization of Propylene Oxide and Carbon Dioxide: Combining High Activity and Selectivity. *Journal of the American Chemical Society* **2005**, *127* (31), 10869-10878. DOI: 10.1021/ja051744l.

(6) Cyriac, A.; Lee, S. H.; Varghese, J. K.; Park, E. S.; Park, J. H.; Lee, B. Y. Immortal CO2/Propylene Oxide Copolymerization: Precise Control of Molecular Weight and Architecture of Various Block Copolymers. *Macromolecules* **2010**, *43* (18), 7398-7401. DOI: 10.1021/ma101259k.

(7) Deacy, A. C.; Phanopoulos, A.; Lindeboom, W.; Buchard, A.; Williams, C. K. Insights into the Mechanism of Carbon Dioxide and Propylene Oxide Ring-Opening Copolymerization Using a Co(III)/K(I) Heterodinuclear Catalyst. *J. Am. Chem. Soc.* **2022**, *144* (39), 17929-17938. DOI: 10.1021/jacs.2c06921.

(8) Kernbichl, S.; Reiter, M.; Mock, J.; Rieger, B. Terpolymerization of β-Butyrolactone, Epoxides, and CO_2: Chemoselective CO_2-Switch and Its Impact on Kinetics and Material Properties. *Macromolecules* **2019**, *52* (21), 8476-8483. DOI: 10.1021/acs.macromol.9b01777.

(9) Lehenmeier, M. W.; Kissling, S.; Altenbuchner, P. T.; Bruckmeier, C.; Deglmann, P.; Brym, A.-K.; Rieger, B. Flexibly Tethered Dinuclear Zinc Complexes: A Solution to the Entropy Problem in CO_2/Epoxide Copolymerization Catalysis? *Angew. Chem., Int. Ed.* **2013**, *52* (37), 9821-9826. DOI: https://doi.org/10.1002/anie.201302157.
